# Comparison of Alkyl-Bridged Bis(N-Heterocyclic Carbene) Nickel Precatalysts: Structure and Catalytic Activity in the Reductive Cleavage and Suzuki–Miyaura Reactions

**DOI:** 10.1021/acsomega.5c07647

**Published:** 2025-11-17

**Authors:** Claudia S. Zhang, Eleanor C. Beams, Abigail L. Moffett, Chuyi Luo, Colin D. McMillen, Anthony R. Chianese, Kerry-Ann Green

**Affiliations:** † Department of Chemistry, 8609 Williams College, Williamstown, Massachusetts 01267, United States; ‡ Department of Chemistry, 2545 Clemson University, Clemson, South Carolina 29634, United States; § Department of Chemistry, 3719 Colgate University, Hamilton, New York 13346, United States

## Abstract

Four new nickel­(II) precatalysts featuring bidentate chelating ethylene-bridged bisbenzimidazolin-2-ylidenes (**3e–3h**) were synthesized and structurally characterized by single crystal X-ray analysis. The catalytic activity of the ethylene-bridged complexes (**3e–3h**) and the previously reported propylene-bridged analogs (**3a–3d**) are compared for the reductive cleavage and Suzuki–Miyaura coupling (SMC) of aryl sulfamates. The propylene-bridged chelate complexes (**3a–3d**) generally exhibit higher catalytic activity relative to their ethylene counterparts. Experimental findings reveal a notable impact of the alkyl bridge length on the synthesis outcomes and catalytic performance of the Ni­(II) complexes. The ligand parameters, percent buried volume (% *V*_Bur_), bite angle and estimated σ-donor properties based on ^1^H NMR measurements of the precursor benzimidazolium salts are reported. The X-ray crystal structure of a rare well-defined bis­(NHC) nickel­(I) chelate complex (**5**) is reported and its catalytic activity in the reductive cleavage and SMC reactions demonstrated.

## Graphical abstract

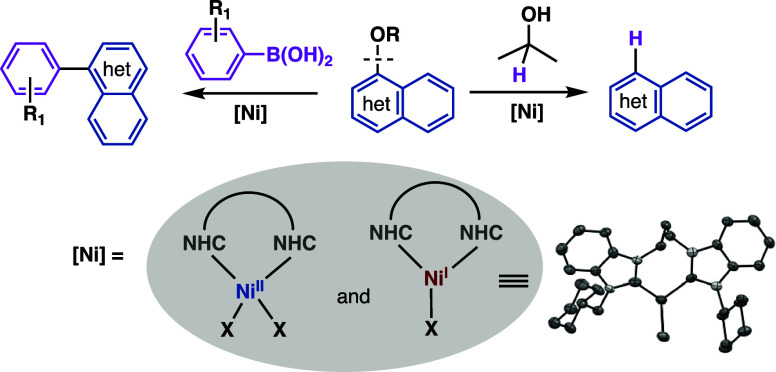

## Introduction

N-heterocyclic carbene (NHC) ligands impart unique properties to their organometallic complexes and have found increasing importance in homogeneous nickel catalysis. NHC ligands are modular, thermally stable and strong electron-donors.^1–8^ Additionally, chelating NHCs offer increased stability to their metal complexes from the chelate effect, thereby minimizing catalyst decomposition pathways such as reductive elimination of the NHC.^9–11^

Reductive cleavage reactions including hydrodehalogenation and hydrodeoxygenation are an important class of reactions for organic synthesis and industrial applications.^1,12^ They are commonly mediated by Ni, Pd and Rh catalysts in combination with hydrogen sources such as formates, borohydrides, silanes, hydrogen gas and alkoxides bearing a beta-hydrogen.^12–17^ Notably, Ni–NHC catalytic systems are increasingly being investigated in reductive cleavage reactions and are often generated in situ from NHC precursors and bench-stable Ni­(II) salts or the air-sensitive Ni­(COD)_2_.^18,19^ These systems have achieved selective C–O bond activations of phenol-derived substrates and the NHC ligand properties are deemed critical to the high activities.^17,20–22^ However, bench-stable, well-defined nickel precatalysts of chelating bidentate bis­(NHCs) have not been explored in reductive cleavage transformations.

In our efforts to understand the structural and catalytic properties of well-defined Ni­(II) complexes featuring chelating bidentate bis­(NHCs), we reported the synthesis and reactivity of propylene-bridged bis­(NHC)­Ni­(II) bromides (**3a–3d**) in the Suzuki–Miyaura coupling (SMC) of aryl sulfamates in a previous study.^23^ In that prior work, we also demonstrated the relevance of Ni­(I) species in the SMC of aryl sulfamates and that bidentate chelating bis­(NHCs) are promising ligand candidates for investigating nickel catalysis.

In this study, we describe the synthesis and structural characterization of four new air-stable Ni­(II) complexes of bidentate ethylene-bridged bis­(NHC) ligands (**3e–3h**). Steric properties of the chelating bis­(NHCs) of complexes **3a–3h** were analyzed using the percent buried volume (% *V*_Bur_) and bite angle parameters, while σ-donor properties were estimated from ^1^H NMR measurements of the precursor bisbenzimidazolium salts.^24,25^ The bis­(NHC) ligands all feature N-wingtip groups with a methylene spacer for flexibility and reduction of steric crowding at the nickel center.^3,26^ The catalytic activity of the bis­(NHC)­Ni­(II) complexes (**3a–3h**) are compared for the reductive cleavage and SMC reactions of phenol-derived electrophiles. The Ni­(II) complexes of the propylene-bridged series (**3a–3d**) are more effective than their corresponding ethylene-bridged counterparts (**3e–3h**), particularly for the SMC reactions. Mechanistic insights for the reductive cleavage of aryl sulfamates with isopropanol (*i*PrOH) as reducing agent are also discussed.

To date, very few well-defined (NHC)­Ni­(I) complexes have been crystallographically characterized,^5,18,27–29^ and there are no literature reports of parameters such as % *V*_Bur_ values for bidentate bis­(NHCs) in Ni­(I) complexes. We also report the crystal structure of the well-defined bis­(NHC)­Ni^I^Br complex (**5**) and demonstrate its activity in the reductive cleavage and SMC reactions.

## Results and Discussion

### Synthesis and Characterization

The ethylene-bridged bisbenzimidazolium bromides (**2e–2h**) were synthesized from the *N*-alkyl benzimidazoles and 1,2-dibromoethane, and were obtained as white powders in yields from 73 to 94% (Scheme 1).^4,30,31^ ^,^ Bisbenzimidazolium bromides (**2f–2h**) have not been previously reported, and all compounds were characterized by ^1^H, ^13^C NMR, and CHN analyses. The Ni­(II) complexes (**3e–3h**) were prepared from the corresponding bisbenzimidazolium bromides and dehydrated Ni­(OAc)_2_. Compounds **3e–3h** were isolated as air-stable, burnt orange crystalline solids in yields of 18–49%. The syntheses for the previously reported propylene-bridged counterparts (**3a–3d**) (Chart 1),^23^ furnished higher yields compared with their ethylene-bridged analogs. The generally lower yields for the ethylene-bridged complexes (**3e–3h**), could be attributed to the formation of several unidentified side-products, which in turn impacted the purification by column chromatography. Notably, **3a–3d** displayed shorter retention times on silica gel, and had higher solubilities in common organic solvents compared to **3e–3h**.

**Scheme 1:**
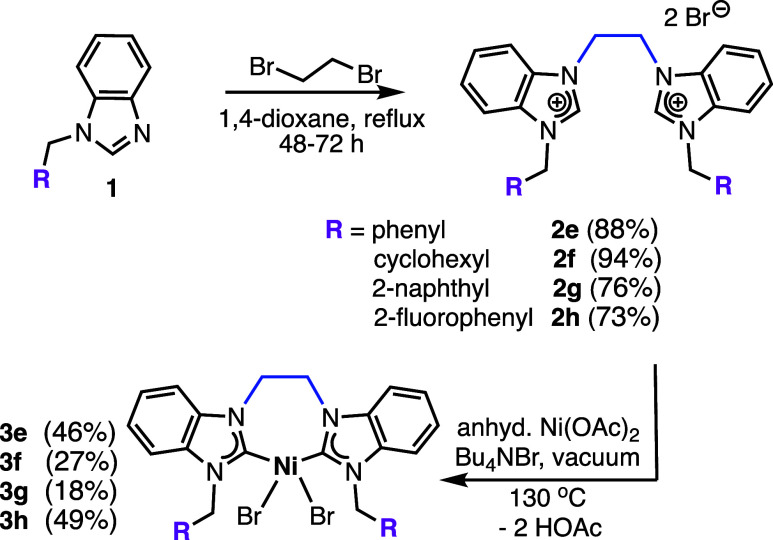
Synthesis of bis­(NHC)­NiBr_2_ Complexes **3e–3h**

**Chart 1:**
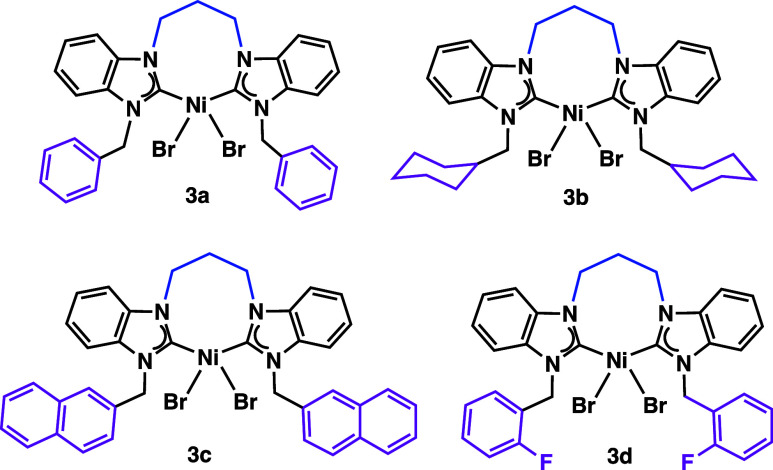
Bis­(NHC)­NiBr_2_ (**3a–3b**) from our prior study^23^

The ^1^H NMR spectra for the bisbenzimidazolium bromides (**2e–2h**), show single resonance signals for the highly deshielded benzimidazole C2 proton (NC**H**N) in the range δ 9.83–10.00 in DMSO-*d*_6_. In the spectra for the corresponding Ni­(II) complexes (**3e–3h**) distinct multiplets for the ethylene bridge protons support the rigidity of the complexes. The ^13^C_carbene_ resonances were not observed for **3e–3h**, despite extended acquisition times, and may be attributed to their lower solubility. In contrast, the ^13^C_carbene_ resonances were observed for all propylene-bridged complexes (**3a–3d**) in the range δ 182.7–183.9 as described in our previous report.^23^ Single crystals of **3e–3h** suitable for X-ray crystallographic analysis were obtained from vapor diffusion of diethyl ether into an acetonitrile solution (**3e–3g**) and layering diethyl ether over a chloroform solution (**3h**). Complexes **3e–3h** are confirmed as *cis*-chelating bis­(NHC)­Ni­(II) mononuclear species that are seven-membered metallacycles (Figures 1, S31 and S32). Similar to their propylene-bridged analogs, the structures for **3e–3h** adopt a slightly distorted square planar geometry about the nickel center (Table S7).

**Figure 1:**
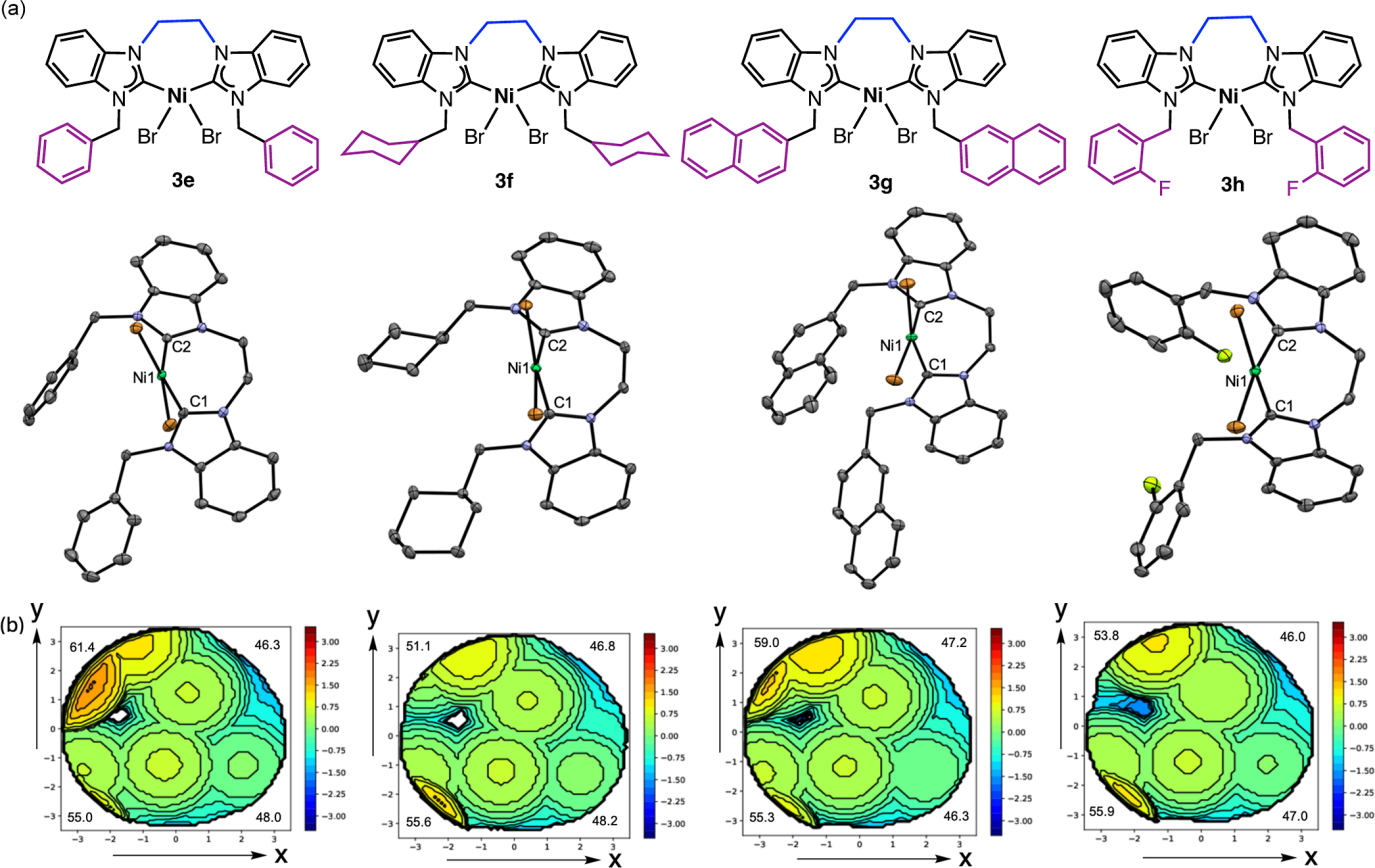
(a) Molecular and X-ray crystal structures of **3e–3h**. Thermal ellipsoids depicted at 50% probability. H atoms and solvent molecules are omitted for clarity. (b) Topographic steric maps of **3e–3h** showing % *V*_Bur_ values per quadrant generated using the SambVca 2.1 web application.

### Bis­(NHC) Ligand Parameters

The influence of the alkyl bridge length on structural parameters of the bis­(NHC) ligands was evaluated from the bite angle (C_1_–Ni–C_2_) and % *V*_Bur_ calculated using the SambVca 2.1 web application^25,32^ (Table 1). The bite angles for **3a–3h** span the range 84.92–88.78° and the % *V*_Bur_ 50.4–53.8%, such that both parameters are only slightly larger for the propylene-bridged ligands compared to their direct ethylene-bridged counterparts with the exception of **3a/3e** (Table 1). The bite angles obtained for **3a–3h** are comparable to those reported for related alkyl-bridged bis­(NHC) complexes of Ni and Pd.^33–36^ The small change in bite angle between the ethylene- and propylene-bridged bis­(NHCs) arises from the angles at which the benzimidazole rings twist relative to the [NiC_2_Br_2_] coordination plane.^34,37^ These (dihedral) angles are significantly larger for the propylene-bridged ligands (Table S5) owing to their greater flexibility, which serves to accommodate the longer bridge and minimize energetically unfavorable conformations.^33,34,38^ Topographic steric maps also generated using the SambVca 2.1 web application^25,32^ reveal similar steric profiles for **3e–3h** with slight crowding in the NW and SW quadrants (Figure 1).

**Table 1:** Ligand Parameters for the Bis­(NHCs) of **3a–3h**

entry	catalyst	bite angle (C–Ni–C, °)	% *V*_Bur_	^1^ *J*_CH_ (Hz)^a^ ^,^ ^b^
1	**3a**	85.89(3)	51.8	219.85
2	**3b**	88.78(9)	53.8	218.45
3	**3c**	87.04(10)	52.3	220.25
4	**3d**	87.06(8)	51.8	219.85
5	**3e**	87.07(9)	52.6	221.20
6	**3f**	86.08(9)	50.4	220.30
7	**3g**	85.61(11)	52.0	221.82
8	**3h**	84.92(14)	50.7	221.35

a NMR spectra for the benzimidazolium salts recorded in DMSO-*d*_6_.

b Determined from ^13^C satellites in the ^1^H NMR spectrum of the benzimidazolium bromides.^24^ SambVca 2.1 parameters: Ni–C distance is crystallographically determined; sphere radius, 3.5 Å; bond radii, 1.17 Å; mesh spacing, 0.1 Å. H atoms excluded.^25^ % *V*_Bur_ and bite angles for **3a–3d** were reported in our prior study.^23^

The σ-donating properties of the bis­(NHCs) were estimated from the ^13^C satellites of the ^1^H NMR measurements from the NC**H**N signal of the precursor benzimidazolium salts (**2a–2h**) in DMSO-*d*_6_. This determination was based on the empirical relationship between the one-bond C–H *J* coupling (^1^ *J*_CH_) and the hybridization (s-character) at the carbon atom involved.^24,39,40^

Calculated ^1^ *J*_CH_ (Hz) values for **3a–3h** spanned the narrow range 218.45–221.82 Hz, and the generally lower values for the propylene-bridged bis­(NHCs) are consistent with stronger σ-donation relative to their corresponding ethylene-bridged counterparts. Based on all three parameters, the propylene-bridged bis­(NHC) of **3b**, featuring the cyclohexylmethyl N-wingtip group is the strongest σ-donor (lowest ^1^ *J*_CH_ value) and is the most sterically demanding with the largest bite angle and % *V*_Bur_.

### Reductive Cleavage

Phenol-derived compounds are attractive electrophilic coupling partners owing to their ease of preparation and synthetic utility in directing arene functionalization.^41–43^ To investigate the scope of the bis­(NHC)­NiBr_2_ precatalysts (**3a–3h**), we began by exploring the C–N coupling of aryl sulfamates and amines, mediated by tertbutoxide bases. In those attempts, only trace quantities of the C–N coupled product were obtained along with the reductive cleavage side product (see Supporting Information). While the bis­(NHC)­NiBr_2_ precatalysts were not effective for C–N coupling under the conditions explored, the results prompted us to investigate the precatalysts in the reductive cleavage of aryl sulfamates. Control experiments performed in the absence of the amine, resulted in the quantitative recovery of the aryl sulfamate pointing to the role of this substrate under reaction conditions. Based on preliminary work, precatalyst **3b** and 1-naphthyl dimethylsulfamate were selected as the model catalyst and substrate, respectively, for exploring the reductive cleavage reaction (Table 2, entry 1). Isopropanol was selected as a more practical and greener hydrogen source to replace the amine.

**Table 2:**
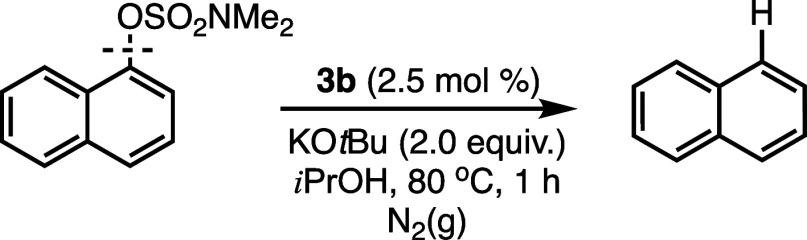
Optimization of the Reductive Cleavage Reaction With **3b**

entry	deviation from standard conditions	yield (%)^a^
1	none	>99
2	50 °C	87
3	room temp (25 °C)	0
4	without Ni precatalyst	0
5	NiBr_2_ (2.5 mol %) instead of **3b**	0
6	**3b** (5.0 mol %), *i*PrOH/toluene (1:10), 24 h, 80 °C	90^b^
7	KO*t*Bu (1.0 equiv)	89
8	without KO*t*Bu	0
9	in air	0

a Standard conditions: **3b** (2.5 mol %), 1-naphthyl dimethylsulfamate (1 equiv), KO*t*Bu (2.0 equiv), *i*PrOH (2.5 mL) under N_2(g)_ at 80 °C for 1 h. Yields were determined by ^1^H NMR analysis and are the average of two independent trials.

b Yield determined from a single trial.

Initially, with **3b** (5 mol %), KO*t*Bu (2 equiv) in a mixture of *i*PrOH: toluene (1:10) at 80 °C for 24 h, naphthalene was obtained in 90% yield (Table 2, entry 6). Transitioning from the toluene cosolvent to neat *i*PrOH and 2.5 mol % of **3b** resulted in the quantitative generation of naphthalene after 1 h (Table 2, entry 1). Two equivalents of the base were required to ensure a high conversion. Control reactions carried out in the absence of **3b** resulted in quantitative recovery of 1-naphthyl dimethylsulfamate, which suggests that a nucleophilic aromatic substitution does not occur under these conditions (Table 2, entry 4).

Furthermore, when **3b** was replaced by commercially available NiBr_2_, 1-naphthyl dimethylsulfamate was recovered quantitatively (Table 2, entry 5). It is worth noting that the model catalyst **3b** is catalytically active at 50 °C, generating naphthalene in 87% yield, but is, however, inactive at room temperature (Table 2, entries 2 and 3). Additionally, the base and inert atmosphere are critical to this reaction, as when separate reactions were performed in the absence of base or in the presence of air, unreacted 1-naphthyl dimethylsulfamate was fully recovered (Table 2, entries 8 and 9). The sensitivity of the reductive cleavage reaction to air is consistent with the involvement of low oxidation state Ni-species as the active catalyst.^3^ A plausible activation pathway for the precatalyst is the reduction of Ni­(II) to Ni(0) promoted by the in situ generated isopropoxide ion (see Supporting Information). The isopropoxide ion has been found to act as a reducing agent for well-defined Ni­(II) and Pd­(II) precatalysts bearing phosphine or NHC ancillary ligands along with halide coligands.^44,45^

The standard reaction conditions were established as 2.5 mol % Ni catalyst, KO*t*Bu (2 equiv), and *i*PrOH at 80 °C for 1 h under a nitrogen atmosphere. The remaining precatalysts (**3a**, **3c–3h**) were investigated with the model substrate 1-naphthyl dimethylsulfamate (Table 3). Both the ethylene- and propylene-bridged systems are catalytically active in the reductive cleavage of 1-naphthyl dimethylsulfamate under standard conditions. However, complex **3b** was the most catalytically active system, furnishing the desired naphthalene product quantitatively (Table 3).

**Table 3:**
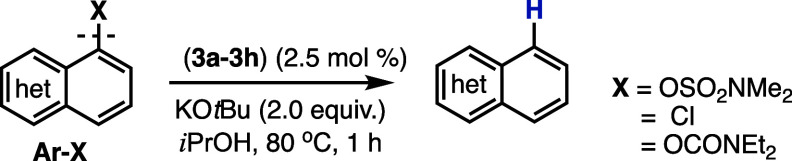

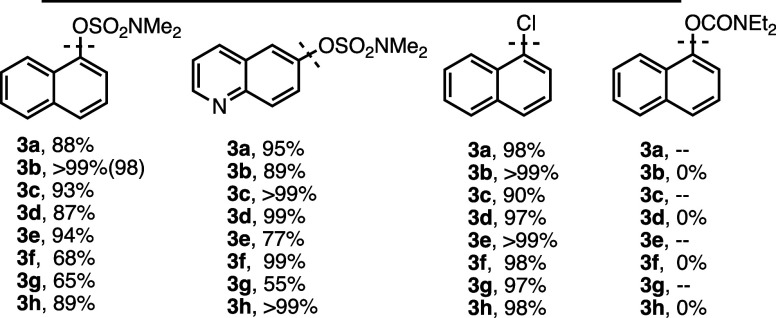
Investigation of Ni-Catalyzed Reductive Cleavage of Phenol-Derived Substrates and 1-Chloronaphthalene with **3a–3h** ^a^

a Reaction conditions: (**3a–3h**) (2.5 mol %), **Ar-X** (1 equiv), KO*t*Bu (2.0 equiv), *i*PrOH (2.5 mL), under *N*_2(g)_ at 80 °C for 1 h. Yields listed below each starting material were determined by ^1^H NMR analysis with 1,3,5-trimethoxybenzene as an internal standard and are the average of two independent trials. For **X** = OCONEt_2_, naphthalene was not detected for **3b**, **3d**, **3f**, and **3h** and the 1-naphthol side product was observed in 0–36% yield. For **X** = OCONEt_2_, table entries are shown as -- for **3a**, **3c**, **3e**, and **3g** as the reductive cleavage was not investigated for these precatalysts. Isolated yield in parentheses.

Precatalysts **3a–3h** were further evaluated for the reductive cleavage of quinolin-6-yl dimethylsulfamate and 1-naphthyl *N*,*N*-diethylcarbamate. All precatalysts were effective in the reductive cleavage of the heteroaromatic substrate, quinolin-6-yl dimethylsulfamate to generate quinoline, with yields ranging from 55 to >99% (Table 3). Precatalysts **3b**, **3d**, **3f** and **3h** were further evaluated for the reductive cleavage of 1-naphthyl *N*,*N*-diethylcarbamate and in all cases the desired naphthalene product was not observed (Table 3). The 1-naphthyl *N*,*N*-diethylcarbamate starting material was, however, recovered in all instances along with varying quantities of the 1-naphthol side product from hydrolysis (see Supporting Information). The lower reactivity of the aryl carbamate relative to the aryl sulfamate in nickel catalysis is documented in the literature. In the Ni-catalyzed SMC reported by Garg et al., the higher reactivity of the aryl sulfamates in oxidative addition relative to the aryl carbamates was attributed to the weaker aryl C–O bond in the sulfamate compared to the aryl C–O bond in the carbamate group based on DFT calculations.^43,46,47^

Although our primary interest was in the reductive cleavage of phenol-derived electrophiles, we also investigated the commonly used 1-chloronaphthalene as a benchmark substrate for comparison. All precatalysts were effective in the hydrodechlorination of 1-chloronaphthalene with yields exceeding 90% (Table 3), and consequently there are no significant differences in catalyst performance across the two series of precatalysts for this substrate. With the facile conversion of the aryl chloride, but inertness of the aryl carbamate under standard conditions of reductive cleavage, the chemoselective reductive cleavage of 4-chloro-1-naphthyl *N*,*N*-diethylcarbamate was successfully achieved with **3b** to furnish 1-naphthyl *N*,*N*-diethylcarbamate (89% yield), with only a minor quantity of 1-naphthol (6% yield) (Scheme S1). This demonstrated selectivity presents opportunities for site selective reduction of chlorophenol derivatives in orthogonal catalysis.

In the case of the 4-chloro-1-naphthyl dimethylsulfamate, the conversion using **3b** was unselective, with the following products and yields: naphthalene (75%), 1-naphthyl dimethylsulfamate (6%), and trace quantities of 1-chloronaphthalene detected by GC–MS (Scheme S1). This result is consistent with the demonstrated higher reactivity of the aryl sulfamate and aryl chloride substrates toward reductive cleavage relative to the aryl carbamate. All eight precatalysts (**3a–3h**) are competent in the reductive cleavage reactions of the aryl sulfamate and 1-chloronaphthalene substrates, however, the propylene-bridged precatalysts are slightly more effective for the aryl sulfamates.

### Mechanistic Insights into the Ni-Catalyzed Reductive Cleavage

For insight into the hydrogen source in the reductive cleavage reactions, we conducted separate reactions of 1-naphthyl dimethylsulfamate in deuterium-labeled isopropanol (*i*PrOH-2*d*_1_), both neat and with toluene as cosolvent (Table 4).

**Table 4:**
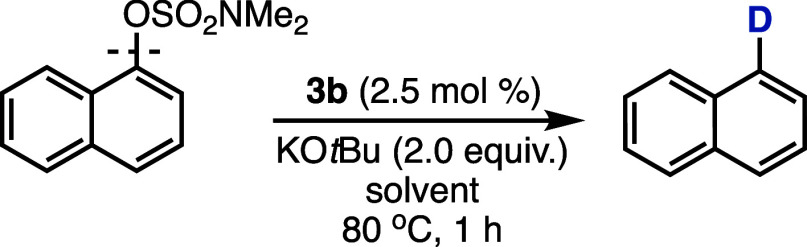
Deuterium Labeling Experiments of the Ni-Catalyzed Reductive Cleavage of Aryl Sulfamate with **3b**

solvent	yield (%)	aryl sulfamate recovery (%)
*i*PrOH-2*d*_1_	56	33
*i*PrOH-2*d*_1_/toluene (1:10)	17	82

Characterization of the naphthalene product from these experiments confirms the substitution of the OSO_2_NMe_2_ group by deuterium, and demonstrates that hydrogen transfer occurs from the hydrogen adjacent to the hydroxyl group in *i*PrOH. The %deuterium incorporation was calculated to be >99% at position-1 by ^1^H NMR analysis. This observed C–O cleavage and deuterium incorporation is consistent with reduction occurring by β-hydride elimination from an isopropoxide ion.^22^ In the case of neat *i*PrOH-2*d*_1_, the deuterated naphthalene was observed in 56% yield with recovered 1-naphthyl dimethylsulfamate (33%, Table 4). When the reaction was performed in the *i*PrOH-2*d*_1_: toluene (1:10) mixture, the yield of deuterated naphthalene was 17% with recovered 1-naphthyl dimethylsulfamate (82%, Table 4). These findings demonstrate the impact of the concentration of the hydrogen source and deuteration on the reaction.

To investigate the possible participation of single electron species in the reductive cleavage reaction, separate experiments were conducted in the presence of the radical trapping agents (2,2,6,6-tetramethylpiperidin-1-yl)­oxyl (TEMPO), butylated hydroxytoluene (BHT) and the galvinoxyl free radical (see Supporting Information). The addition of each radical scavenger (1.0 equiv) under the otherwise standard conditions did not result in the formation of the naphthalene product from reductive cleavage (see Supporting Information). The complete inhibition of reductive cleavage with quantitative recovery of 1-naphthyl dimethylsulfamate is consistent with the participation of the nickel catalyst in single electron transfer processes. In the case of 1-chloronaphthalene, only a trace amount of naphthalene (5% yield) was obtained, with 94% recovered starting material when TEMPO was used as a radical scavenger (see Supporting Information). Based on these findings and the absence of reductive cleavage when the reaction was conducted in air, we thought it necessary to investigate the possible involvement of Ni­(I) species in the reaction pathway. Our hypothesis was that Ni­(I) species could be generated during precatalyst activation from comproportionation events of Ni­(II) and Ni(0) species in the mixture. The feasibility of such an off-cycle process is well-documented in the literature on related Ni­(II) complexes.^48,49^ Heating a mixture of **3b**, KO*t*Bu (76 equiv) and *i*PrOH-*d*_8_ (0.6 mL) in a J-Young NMR tube under a nitrogen atmosphere resulted in a rapid color change from yellow to intense brown upon heating, with broadening of the ^1^H NMR signals and a reduced signal-to-noise ratio (see Figure S1) which could be indicative of the generation of paramagnetic material such as Ni­(I) species. Consequently, we accessed the well-defined bis­(NHC)­Ni^I^Br complex and investigated its reactivity in reductive cleavage.

### Structure and Reactivity of Bis­(NHC)­Ni^I^Br (**5**)

The bis­(NHC)­Ni^I^Br monochelate (**5**) was synthesized by treating **3b** with Zn powder in THF at ambient temperature for 24 h in a nitrogen-filled glovebox. Following recrystallization, complex **5** was obtained as an orange solid in 25% yield. Notably, **5** was isolated without any associated ZnBr_2_. Complex **5** is stable at room temperature inside the glovebox in the solid state, and in solution even after 11 days in C_6_D_6_. Crystals suitable for single crystal X-ray analysis were obtained by layering diethyl ether over a THF solution at room temperature. From the X-ray crystallographic data, **5** exhibits a distorted trigonal-planar geometry about the Ni center (Figure 2).

**Figure 2:**
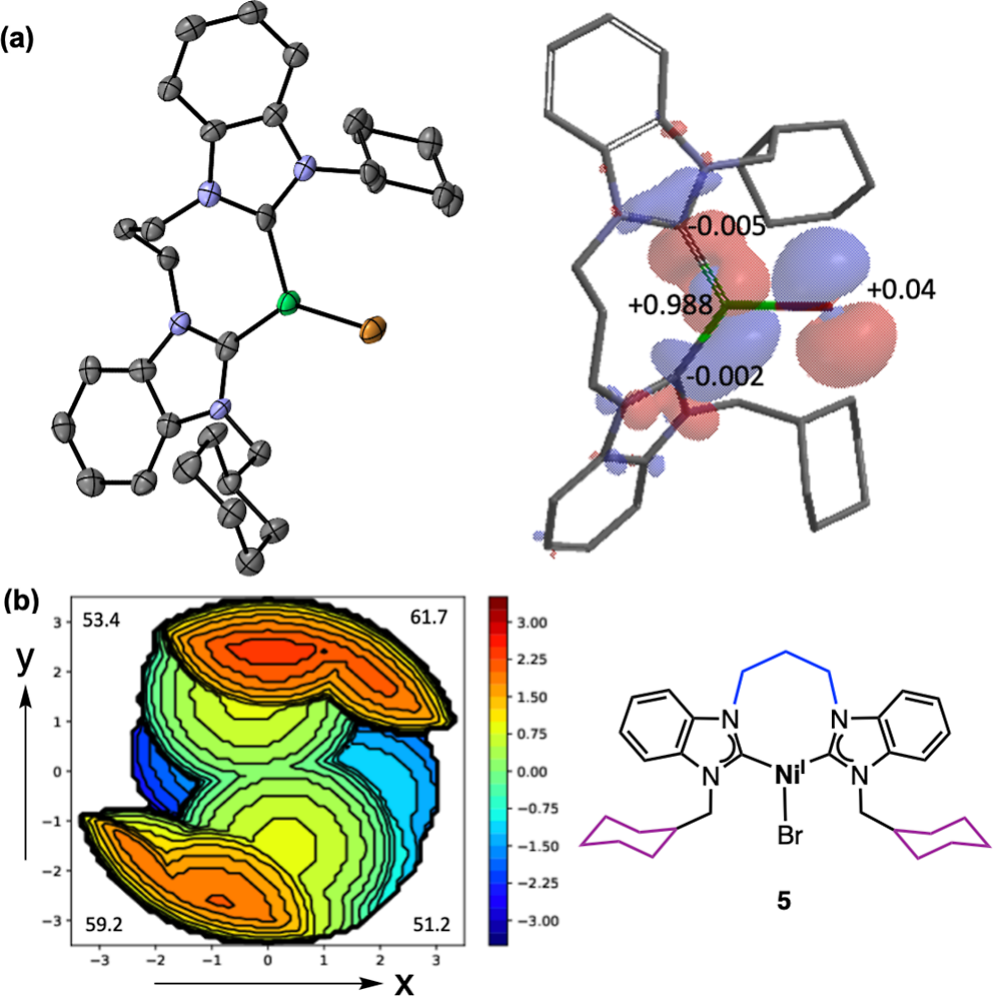
(a) X-ray structure of bis­(NHC)­Ni^I^Br (**5**) at 50% probability thermal ellipsoids and spin density plot determined by DFT analysis. Mulliken spin density population indicated for selected atoms. Hydrogen atoms and one molecule in the asymmetric unit have been omitted for clarity. (b) Topographic steric map of **5** generated using the SambVca 2.1 web application with ORTEP diagram viewed down the Ni–Br bond (*z*-axis) and molecular structure of **5**. Selected bond lengths (Å): C–Ni = 1.924(7) and Ni–Br = 2.3600(12). Selected bond angles (°): C–Ni–C = 109.8(3) and C–Ni–Br = 124.7(2). Averaged values of the two molecules in the asymmetric unit are reported.

There are only limited examples of well-defined Ni­(I) complexes of NHCs, and as a result, their relevance or role in catalysis is not well-understood. Furthermore, well-defined Ni­(I) complexes of bidentate bis­(NHC) ligands are largely unknown.^5,18,29^ To the best of our knowledege this is the first reported crystal structure of a well-defined *cis*-chelating alkyl-bridged bis­(NHC)­Ni­(I)­X complex (where X = Br).

In comparing ligand parameters for the bis­(NHC) in **5** with its precursor **3b**, the % *V*_Bur_ values are 53.8 (**3b**) and 56.4 (**5**), with corresponding bite angles 88.78(9)° (**3b**) and 109.8(3)° (**5**). Based on these parameters, there is a notable change in the steric demand of the bis­(NHC) ligand upon reduction of Ni­(II) to Ni­(I), where the effective steric size of the bis­(NHC) ligand increases. From the topographic steric map of **5** (Figure 2), the NE and SW quadrants are more sterically crowded owing to the cyclohexylmethyl N-wingtip groups, while the remaining quadrants are significantly less crowded. Geometry optimizations and single-point energy calculations were carried out at a fixed geometry of **5** given by the crystallographic coordinates at the B3LYP level of theory with the basis set 6-31G* using the Spartan 24 program. The computed Mulliken spin density reveals that the unpaired electron in **5** is primarily localized on the Ni center (0.99) with a small portion distributed to the bromine (0.04). This is consistent with reports of trigonal planar Ni­(I) complexes which commonly feature metal-centered radical character.^50^

To verify the catalytic relevance of a bis­(NHC)­Ni­(I) species in the reductive cleavage of aryl sulfamates, we examined the reactivity of **5** with the model substrate, 1-naphthyl dimethylsulfamate (Scheme 2). With precatalyst **5** (2.0 mol %) under otherwise standard conditions, 1-naphthyl dimethylsulfamate was successfully converted to naphthalene with an average yield of 89%, after three independent trials (see Supporting Information). Clearly, the Ni­(I) precatalyst effectively catalyzes the reductive cleavage reaction and supports the relevance of Ni­(I) species to the reductive cleavage transformation. This result also implies that Ni­(I) species generated via a comproportionation pathway under reaction conditions could lead to productive catalysis.

**Scheme 2:**
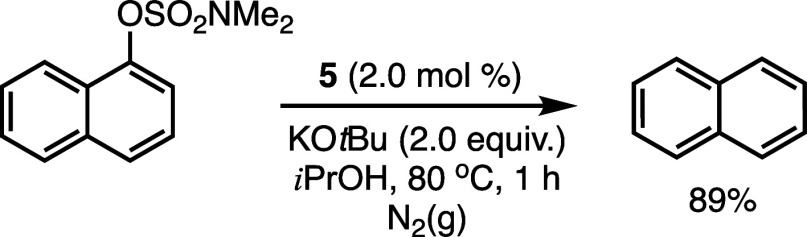
Catalytic Activity of **5** in the Ni-Catalyzed Reductive Cleavage of 1-Naphthyl Dimethylsulfamate

### Suzuki–Miyaura Coupling

With the demonstrated catalytic activity of the propylene-bridged precatalysts (**3a–3d**) for the SMC in our previous study,^23^ we investigated the ethylene-bridged counterparts and the possible impact of the alkyl bridge length on catalytic performance. The model reaction involved 1-naphthyl dimethylsulfamate and the electron-rich 4-methoxyphenylboronic acid, and a catalyst loading of 2.5 mol % [Ni]. Under the model conditions, the yield of product **4a** was markedly lower for all ethylene-bridged precatalysts (26–85%), compared to the propylene-bridged counterparts (72–93%) (Table 5). Notably, higher yields of **4a** (61–99%) were obtained for **3e–3h** when the catalyst loading was increased to 5 mol % (Table 5). While all eight precatalysts (**3a–3h**) are competent in the SMC for the electron-rich 4-methoxyphenylboronic acid, all precatalysts exhibited lower activity when evaluated with the less activated substrates. Notably, the reaction of phenyl dimethyl sulfamate (a more challenging substrate lacking a fused ring) with 4-methoxyphenylboronic acid to generate the coupled product **4e**, was conducted under more driving conditions of 80 °C for 16 h. The catalytic performance for the ethylene-bridged complexes (**3e–3h**) was especially poor in generating products **4b**, **4c** and **4e**, with yields in the range of 0–39%, compared to 31–84% for the propylene-bridged systems (**3a–3d**). The propylene-bridged systems (**3a–3d**) are generally more effective catalysts than their ethylene-bridged counterparts (**3e–3h**) for all substrates explored.^23^ For the heteroaromatic substrate quinolin-6-yl dimethylsulfamate, at 80 °C for 24 h, high catalytic activities were observed for precatalysts **3a–3d**, **3f** and **3h** where the product, **4d**, was generated almost quantitatively. Interestingly, **3a–3d** and **3h** also furnished this heteroaromatic coupled product (**4d**) under milder conditions of 60 °C for 1 h in yields of 46–89%. Despite the lower activity of the ethylene-bridged series, precatalysts **3f** and **3h** consistently generated coupled products for all reactions, while precatalysts **3e** and **3g** were ineffective in furnishing products **4b** and **4c**.

**Table 5:**
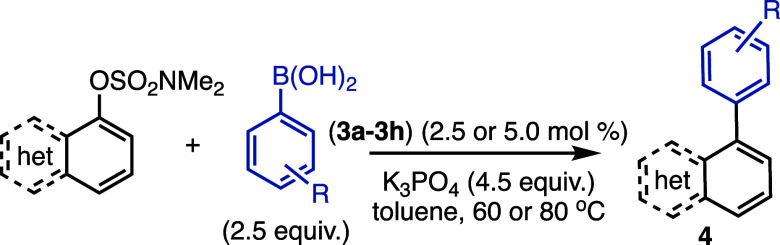

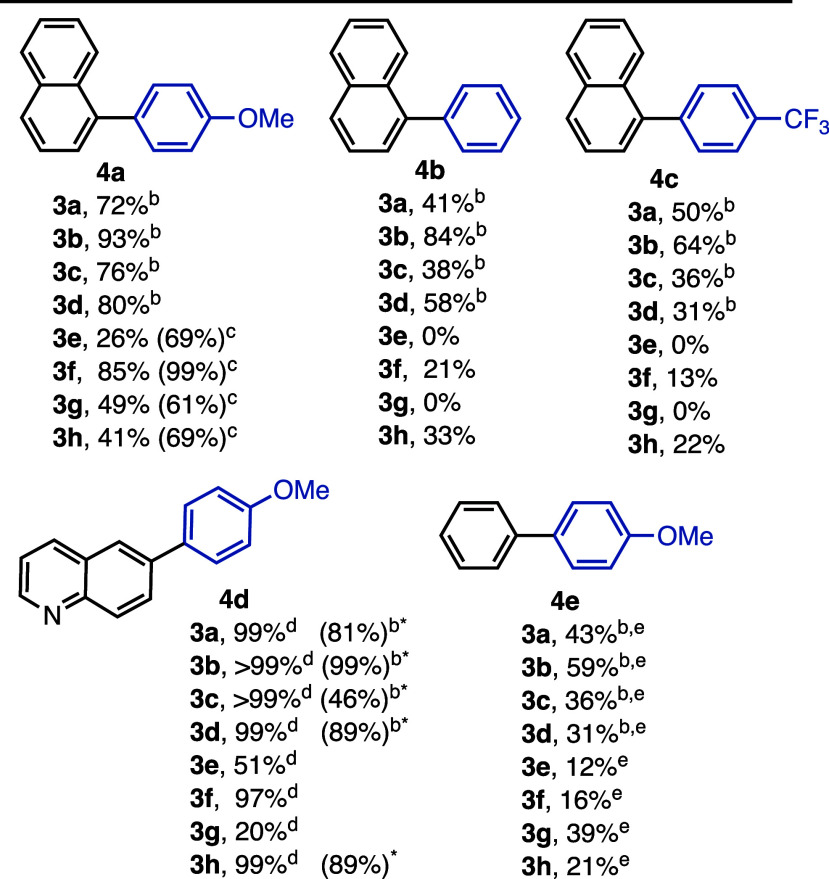
Investigation of the SMC of Aryl Sulfamates and Aryl Boronic Acids with **3a–3h** ^a^

a Reaction conditions: (**3a–3h**) (2.5 mol %), aryl sulfamate (1 equiv), aryl boronic acid (2.5 equiv), K_3_PO_4_ (4.5 equiv), toluene (2.5 mL), 4 h at 60 °C. Yields listed below each starting material were determined by ^1^H NMR analysis with 1,3,5-trimethoxybenzene as an internal standard and are the average of two independent trials.

b Yields for **3a–3d** were reported in a prior study.^23^ *time = 1 h.

c **3e–3h** (5.0 mol %).

d 80 °C, 24 h.

e 80 °C, 16 h.

As in the case of **3a–3d**, indirect evidence for the generation of active Ni(0) species was obtained from the observation of the biphenyl products from homocoupling of the arylboronic acids in all experiments involving **3e–3h**, thereby supporting a similar catalyst activation pathway via boron to nickel transmetalation.^23^ However, the generally slower activation of the ethylene-bridged precatalysts (**3e–3h**) relative to their propylene-bridged counterparts could play a role in their lower overall catalytic performance in the SMC.

It is worth noting that of the eight precatalysts investigated, the propylene-bridged system **3b** was the most active (i.e., higher yields), significantly outperforming its ethylene-bridged analog (**3f**), which also features the cyclohexylmethyl N-wingtip groups. It is apparent that the stronger σ-donating properties and generally greater steric demand of the propylene-bridged bis­(NHCs) play a role in the observed higher catalytic performance of their corresponding nickel complexes (**3a–3d**). However, additional considerations such as the dynamic behavior of the alkyl bridges in combination with the flexible N-wingtip groups are needed to fully rationalize the observed reactivity of the two classes of precatalysts.^51–54^

Finally, the well-defined bis­(NHC)­Ni^I^Br monochelate complex **5** was also catalytically active in the SMC generating compound **4a** in 94% yield in the model reaction at 2.5 mol % (Scheme 3). The observation of 4,4′-dimethoxy-1,1′-biphenyl by GCMS is consistent with a similar activation pathway for **5** and its Ni­(II) counterpart **3b** as reported in our previous study.^23^ Overall this finding supports the relevance of (bisNHC)­Ni­(I) species in the SMC of aryl sulfamates.

**Scheme 3:**
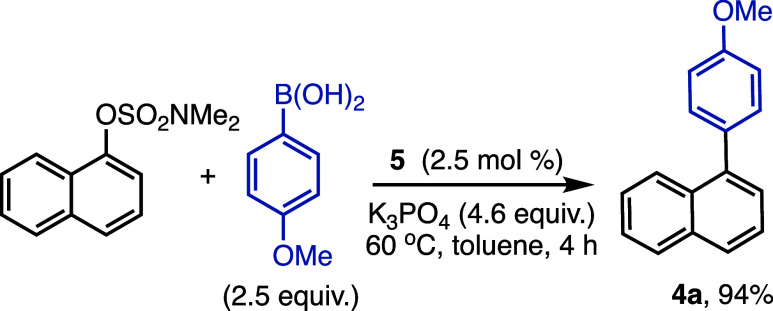
Catalytic Activity of **5** in the Ni-Catalyzed SMC of 1-Naphthyl Dimethylsulfamate and 4-Methoxyphenylboronic Acid

## Conclusions

In summary, the synthesis and characterization of four well-defined ethylene-bridged bis­(NHC)­NiBr_2_ complexes (**3e–3h**) are described. The structural and catalytic properties of the ethylene- and previously reported propylene-bridged complexes are compared for the reductive cleavage and SMC reactions of aryl sulfamates. All precatalysts are catalytically active in the reductive cleavage of aryl sulfamates and 1-chloronaphthalene using *i*PrOH as the reducing agent, with the propylene-bridged analogs being generally more effective. Deuterium labeling experiments identify the hydrogen adjacent to the hydroxyl group in *i*PrOH as the hydrogen source in the reductive cleavage and the %D incorporation was determined to be >99%. Radical scavengers completely inhibit the reductive cleavage reaction.

The propylene-bridged series of Ni­(II) complexes (**3a–3d**) are superior precatalysts for the SMC reactions of aryl sulfamates under reasonably mild conditions and the precatalyst featuring cyclohexylmethyl N-wingtip groups (**3b**) has demonstrated the highest catalytic activity across both reaction types. Our findings demonstrate that the alkyl bridge length of the bis­(NHCs) impacts the synthesis outcomes and catalytic activity of the Ni­(II) complexes. Both series of precatalysts generally show higher catalytic activity for the reductive cleavage than for the SMC of aryl sulfamates and precatalysts **3a–3h** demonstrate considerable promise for the catalytic hydrodechlorination and hydrodeoxygenation reactions.

The crystal structure of a bis­(NHC)­Ni^I^Br monochelate (**5**), is reported and this complex is an effective catalyst in both the reductive cleavage and SMC reactions of the model aryl sulfamate, thereby supporting the relevance of Ni­(I) species under catalytic conditions. Our group continues to explore the potential and diverse opportunities that well-defined bis­(NHC)Ni chelate complexes afford.

## Experimental Section

### General Considerations and Materials

All air- and moisture-sensitive procedures were conducted using standard Schlenk techniques or in a nitrogen-filled glovebox. 1-Benzyl-1*H*-benzo­[*d*]­imidazole (**1a**),^55,56^ 1-(cyclohexylmethyl)-1*H*-benzo­[*d*]­imidazole (**1b**),^57^ 1-(naphthalen-2-ylmethyl)-1*H*-benzo­[*d*]­imidazole (**1c**),^56^ 1-(2-fluorobenzyl)-1*H*-benzo­[*d*]­imidazole (**1d**), 1-naphthyl dimethylsulfamate, phenyl dimethylsulfamate, quinolin-6-yl dimethylsulfamate and 4-chloro-1-naphthyl dimethylsulfamate were prepared according to modified literature procedures.^23,43,58^ Precatalysts **3a–3d** were synthesized according to the procedure reported in our previous study.^23^ Nickel­(II) bromide, zinc powder, isopropanol, *i*PrOH-2*d*_1_, potassium phosphate tribasic, phenylboronic acid, 4-(trifluoromethyl)­phenylboronic acid, 4-methoxyphenylboronic acid, galvinoxyl and butylated hydroxytoluene (BHT) were obtained from Sigma-Aldrich. Potassium *tert*-butoxide and 2,2,6,6-tetramethyl-1-piperidinyloxy (TEMPO) were obtained from Oakwood Chemical. The solvent *i*PrOH-*d*_8_ (+99%D) was obtained from Thermoscientific. Dehydrated Ni­(OAc)_2_ was obtained by heating Ni­(OAc)_2_.4H_2_O sourced from Alfa Aesar in a round-bottom flask on a Schlenk line under dynamic vacuum at 125 °C for 8 h. Dry toluene was collected from a JC Meyer solvent purification system in a Straus flask and stored in the glovebox. All other chemicals including solvents were obtained commercially and used as received unless otherwise stated.

### Representative Procedure for the Synthesis of the Bisbenzimidazolium Salts **2e–2h**

All alkyl-bridged bisbenzimidazolium bromides were prepared from the direct reaction of the N-substituted benzimidazole and the corresponding dihaloalkane.^30^ A Schlenk flask equipped with a PTFE-coated stir bar was charged with the *N*-substituted benzimidazole (**1a–1d**) (2.0–2.1 equiv), 1,2-dibromoethane (1.0 equiv) and 1,4-dioxane. The reaction flask was sealed, then evacuated and backfilled with nitrogen for three cycles. A nitrogen balloon was inserted via the septum and the mixture was heated at 100 °C for 20–72 h. A white precipitate formed over time and at the end of the heating period, the reaction mixture was filtered via a Hirsch funnel and the product rinsed with THF followed by diethyl ether. All bisbenzimidazolium salts were isolated as white powdery solids.

### 1,1′-Dibenzyl-3,3′-(1,2-ethanediyl)­bisbenzimidazolium Dibromide (**2e**)

The compound was synthesized from **1a** (995 mg, 4.78 mmol, 2.1 equiv) and 1,2-dibromoethane (0.2 mL, 2.28 mmol, 1.0 equiv) and 1,4-dioxane (6 mL). The mixture was heated at reflux for 72 h. Yield 1.217 g, (88%). ^1^H NMR (500 MHz, DMSO-*d*_6_) δ: 10.00 (s, 2H), 7.93 (d, *J* = 8.4 Hz, 2H), 7.84 (d, *J* = 8.4 Hz, 2H), 7.60 (t, *J* = 8.3 Hz, 2H), 7.49–7.38 (m, 12H), 5.76 (s, 4H), 5.20 (s, 4H). ^13^C NMR (126 MHz, DMSO-*d*_6_) δ: 143.2, 133.6, 131.2, 130.7, 129.0, 128.8, 128.34, 126.9, 126.8, 114.0, 113.1, 50.0, 45.9. The NMR data are consistent with those reported in the literature.^31^

### 1,1′-Di­(cyclohexylmethyl)-3,3′-(1,2-ethanediyl)­bisbenzimidazolium Dibromide (**2f**)

The compound was synthesized from **1b** (827 mg, 3.86 mmol, 2.1 equiv) and 1,2-dibromoethane (0.16 mL, 1.84 mmol, 1.0 equiv) and 1,4-dioxane (6 mL). The mixture was heated at reflux for 72 h. Yield 1.069 g, (94%). ^1^H NMR (500 MHz, DMSO-*d*_6_) δ: 9.83 (s, 2H), 8.12 (d, *J* = 8.3 Hz, 2H), 7.92 (d, *J* = 8.3 Hz, 2H), 7.68 (m, 2H), 7.61 (m, 2H), 5.16 (s, 4H), 4.30 (d, *J* = 7.2 Hz, 4H), 1.81 (m, 2H), 1.65 (m, 6H), 1.46 (m, 4H), 1.11 (m, 6H), 1.00–0.83 (m, 4H). ^13^C NMR (126 MHz, DMSO-*d*_6_) δ: 143.0, 131.4, 130.9, 126.9, 126.8, 114.0, 113.1, 52.2, 45.7, 37.1, 29.4, 25.5, 25.0. Calcd for C_30_H_40_Br_2_N_4_.2.5H_2_O: C, 54.47; H, 6.86; N, 8.47. Found: C, 54.46; H, 6.78; N, 8.40.

### 1,1′-Di­(naphthalen-2-ylmethyl)-3,3′-(1,2-ethanediyl)­bisbenzimidazolium Dibromide (**2g**)

The compound was synthesized from **1c** (1.36 g, 5.25 mmol, 2.0 equiv) and 1,2-dibromoethane (0.22 mL, 2.54 mmol, 1.0 equiv) and 1,4-dioxane (8 mL). The mixture was heated at reflux for 48 h. Yield 1.35 g, (76%). ^1^H NMR (500 MHz, DMSO-*d*_6_) δ: 9.90 (s, 2H), 8.08 (s, 2H), 7.99–7.90 (m, 8H), 7.85 (d, *J* = 8.4 Hz, 2H), 7.62–7.53 (m, 6H), 7.46 (m, 4H), 5.88 (s, 4H), 5.17 (s, 4H). ^13^C NMR (126 MHz, DMSO-*d*_6_) δ: 143.3, 132.8, 132.7, 131.2, 131.0, 130.8, 128.8, 127.9, 127.8, 127.7, 126.90, 126.87, 126.81, 126.75, 125.6, 114.0, 113.1, 50.3, 45.9. Calcd for C_38_H_32_Br_2_N_4_.0.5H_2_O: C, 63.97; H, 4.66; N, 7.85. Found: C, 63.69; H, 4.61; N, 7.89.

### 1,1′-Di-(2-fluorobenzyl)-3,3′-(1,2-ethanediyl)­bisbenzimidazolium Dibromide (**2h**)

The compound was synthesized from **1d** (3.24g, 14.3 mmol, 2.0 equiv) and 1,2-dibromoethane (0.62 mL, 7.2 mmol, 1.0 equiv) and 1,4-dioxane (12 mL). The mixture was heated at reflux for 72 h. Yield 3.23 g, (73%). ^1^H NMR (500 MHz, DMSO-*d*_6_) δ: 10.00 (s, 2H), 7.99 (d, *J* = 8.4 Hz, 2H), 7.90 (d, *J* = 8.4 Hz, 2H), 7.65–7.61 (m, 4H), 7.54–7.46 (m, 4H), 7.33–7.25 (m, 4H), 5.83 (s, 4H), 5.18 (s, 4H). ^13^C NMR (126 MHz, DMSO-*d*_6_) δ: 160.5 (d, *J* = 247.1 Hz), 143.4, 131.5 (d, *J* = 8.2 Hz), 131.2 (d, *J* = 2.8 Hz), 131.1, 130.7, 127.1, 126.8, 125.0 (d, *J* = 3.5 Hz), 120.7 (d, *J* = 14.2 Hz), 116.0 (d, *J* = 20.5 Hz), 113.7, 113.2, 45.8, 44.6 (d, *J* = 3.6 Hz). ^19^F NMR (471 MHz, DMSO-*d*_6_): δ −116.47 (unreferenced). Calcd for C_30_H_26_Br_2_F_2_N_4_: C, 56.27; H, 4.09; N, 8.75. Found: C, 56.08; H, 3.97; N, 8.73.

### Representative Procedure for the Synthesis of Nickel Complexes **3e–3h**

The well-defined nickel complexes (**3e–3h**) were prepared by a solvent-free synthesis.^30^ To a Schlenk flask, equipped with a PTFE-coated stir bar was added dehydrated Ni­(OAc)_2_ (1.0–1.1 equiv), the bisbenzimidazolium salt (**2e–2h**) (1.0 equiv) and tetrabutylammonium bromide (4.3–8.0 equiv). The flask was sealed with a rubber septum, connected to the Schlenk line, and placed in a preheated oil bath. The mixture was initially heated at 90 °C with stirring under dynamic vacuum for 30–45 min. The temperature was raised to 130 °C and the mixture heated with stirring under dynamic vacuum at this temperature for 4–7 h. The resulting viscous yellow-green slurry was cooled. Water was then added to dislodge the hardened solid with agitation from an ultrasonic bath. The yellow powder was isolated by vacuum filtration using a Hirsch funnel and the residue rinsed with copious amounts of water. The complex was further purified by flash column chromatography on silica gel by gradient elution (EtOAc: DCM 1:1.2 to 1:1).

### Dibromido-1,1′-dibenzyl-3,3′-(1,2-ethanediyl)­dibenzimidazolin-2,2′-diylidenenickel­(II) (**3e**)

The compound was synthesized from **2e** (1.623 g, 2.69 mmol, 1.0 equiv) and Ni­(OAc)_2_ (0.513 g, 2.90 mmol, 1.1 equiv) and tetrabutylammonium bromide (5.30 g, 16.4 mmol, 6.1 equiv). The mixture was heated under vacuum for 5.5 h. Compound **3e** was an orange crystalline solid after column purification. Yield 816 mg, (46%). ^1^H NMR (500 MHz, DMSO-*d*_6_) δ: 7.76 (d, *J* = 8.2 Hz, 2H), 7.37–7.29 (m, 8H), 7.19 (t, *J* = 8.2 Hz, 2H), 7.11–7.06 (m, 6H), 6.38 (br s, 2H), 6.17 (d, *J* = 16.6 Hz, 2H), 5.55 (d, *J* = 15.6 Hz, 2H), 5.30 (d, *J* = 7.2 Hz, 2H). ^13^C NMR (126 MHz, DMSO-*d*_6_) δ: 135.9, 134.20, 134.15, 128.7, 127.9, 127.0, 123.6, 123.4, 111.1, 111.0, 51.0, 44.0. The Ni–C_carbene_ signal was not observed. Calcd for C_30_H_26_Br_2_N_4_Ni: C, 54.51; H, 3.96; N, 8.48. Found: C, 54.95; H, 4.13; N, 8.43. Combustion analysis was high in carbon despite multiple attempts. Crystals suitable for single crystal X-ray diffraction analysis were obtained from the vapor diffusion of diethyl ether into an acetonitrile solution at ambient temperature.

### Dibromido-1,1′-dicyclohexylmethyl-3,3′-(1,2-ethanediyl)­dibenzimidazolin-2,2′-diylidenenickel­(II) (**3f**)

The compound was synthesized from **2f** (986 mg, 1.60 mmol, 1.0 equiv) and Ni­(OAc)_2_ (283 mg, 1.60 mmol, 1.0 equiv) and tetrabutylammonium bromide (3.0 g, 9.3 mmol, 5.8 equiv). The mixture was heated under vacuum for 4 h. Compound **3f** was a yellow crystalline solid after column purification. Yield 292 mg, (27%). ^1^H NMR (500 MHz, DMSO-*d*_6_) δ: 7.71–7.67 (m, 4H), 7.33–7.27 (m, 4H), 6.44 (br s, 2H), 5.23 (br s, 2H), 4.74 (s, 4H), 2.44 (m, 2H), 1.67–1.47 (m, 10H), 1.23–1.18 (m, 10H). ^13^C NMR (126 MHz, DMSO-*d*_6_) δ: 188.1 (Ni–C), 135.0, 133.8, 123.4, 123.2, 111.4, 110.7, 54.0, 44.0, 38.1, 30.2, 25.7, 25.3. The Ni–C_carbene_ signal was not observed. Calcd for C_30_H_38_Br_2_N_4_Ni: C, 53.53; H, 5.69; N, 8.32. Found: C, 53.99; H, 5.66; N, 8.11. Combustion analysis was high in carbon despite multiple attempts. Crystals suitable for single crystal X-ray diffraction analysis were obtained from the vapor diffusion of diethyl ether into an acetonitrile solution at ambient temperature.

### Dibromido-1,1′-dinaphthalen-2-ylmethyl-3,3′-(1,2-ethanediyl)­dibenzimidazolin-2,2′-diylidenenickel­(II) (**3g**)

The compound was synthesized from **2g** (412.5 mg, 0.586 mmol, 1.0 equiv) and Ni­(OAc)_2_ (103.8 mg, 0.587 mmol, 1.0 equiv) and tetrabutylammonium bromide (1.5 g, 4.7 mmol, 8.0 equiv). The mixture was heated under vacuum for 6 h. Compound **3g** was a burnt orange crystalline solid after column purification. Yield 81 mg, (18%). ^1^H NMR (500 MHz, CDCl_3_) δ: 7.79 (d, *J* = 8.1 Hz, 2H), 7.62 (d, *J* = 8.4 Hz, 2H), 7.54–7.48 (m, 4H), 7.43–7.39 (m, 4H), 7.29 (s, 2H), 7.23–7.20 (m, 2H), 7.10 (d, *J* = 10.4 Hz, 2H), 7.07–7.02 (m, 2H), 6.81 (d, *J* = 8.4 Hz, 2H), 6.77 (m, 2H), 6.08 (d, *J* = 15.4 Hz, 2H), 5.43 (d, *J* = 15.9 Hz, 2H), 5.05 (m, 2H). ^13^C NMR (126 MHz, CDCl_3_) δ: 135.2, 134.6, 133.3, 133.1, 133.0, 128.8, 128.1, 127.9, 126.63, 126.61, 126.5, 125.4, 123.8, 123.6, 111.5, 109.3, 52.2, 44.1. The Ni–C_carbene_ signal was not observed. Calcd for C_38_H_30_Br_2_N_4_Ni: C, 59.96; H, 3.97; N, 7.36. Found: C, 60.07; H, 3.87; N, 7.27. Crystals suitable for single crystal X-ray diffraction analysis were obtained from liquid–liquid diffusion of diethyl ether into an acetonitrile solution at ambient temperature.

### Dibromido-1,1′-di-(2-fluorobenzyl)-3,3′-(1,2-ethanediyl)­dibenzimidazolin-2,2′-diylidenenickel­(II) (**3h**)

The compound was synthesized from **2h** (1.0260 g, 1.60 mmol, 1.0 equiv) and Ni­(OAc)_2_ (288.1 mg, 1.63 mmol, 1.0 equiv) and tetrabutylammonium bromide (2.2 g, 6.9 mmol, 4.3 equiv). The mixture was heated under vacuum for 4 h. Compound **3h** was a dark orange crystalline solid after column purification. Yield 546 mg, (49%). ^1^H NMR (500 MHz, DMSO-*d*_6_) δ: 7.79 (d, *J* = 8.2 Hz, 2H), 7.42–7.33 (m, 4H), 7.31–7.27 (m, 2H), 7.25–7.20 (m, 4H), 6.97–6.94 (m, 2H), 6.70–6.68 (m, 2H), 6.38 (s, 2H), 6.13–5.98 (m, 2H), 5.75 (m, 2H), 5.31 (m, 2H). ^13^C NMR (126 MHz, DMSO-*d*_6_) δ: 159.8 (d, *J* = 246.1 Hz), 134.1 (d, *J* = 2.7 Hz), 130.0 (d, *J* = 8.2 Hz), 128.8 (d, *J* = 3.6 Hz), 124.4 (d, *J* = 3.2 Hz), 123.8, 123.6, 122.9 (d, *J* = 13.6 Hz), 115.6 (d, *J* = 20.4 Hz), 111.1, 110.8, 45.1 (d, *J* = 5.0 Hz), 44.1. The Ni–C_carbene_ signal was not observed. ^19^F NMR (471 MHz, DMSO-*d*_6_) δ: −117.28 (unreferenced). Calcd for C_30_H_24_Br_2_F_2_N_4_Ni: C, 51.69; H, 3.47; N, 8.04. Found: C, 51.92; H, 3.37; N, 7.99. Crystals suitable for single crystal X-ray diffraction analysis were obtained by layering diethyl ether over a chloroform solution at ambient temperature.

### Bis­(NHC)­Ni^I^Br (**5**)

Inside a nitrogen-filled glovebox, an oven-dried 20 mL scintillation vial equipped with a Teflon coated stir bar was charged with **3b** (183.3 mg, 0.27 mmol), zinc powder (878.9 mg, 13.4 mmol, 50 equiv) and THF (6 mL). The mixture was allowed to stir vigorously at RT for 24 h. The resulting dark brown mixture with unreacted zinc was filtered using 0.45 μm syringe filter followed by a second filtration via a layer of Celite in a pipet plugged with cotton. The dark brown solution was concentrated in vacuo, after which pentane (6 mL) was added to precipitate the solid. The product was isolated by vacuum filtration via a sintered glass funnel and the solid rinsed with pentane. Recrystallization: The dark green solid sample was taken in THF (4 mL) in a scintillation vial and layered with Et_2_O (5 mL) then placed in the freezer at −30 °C for 48 h. The dark upper layer was decanted, using a pipet. A fresh batch of Et_2_O (5 mL) was added to the sample and decantation repeated. A bright orange-yellow crystalline solid was obtained. Residual Et_2_O was removed in vacuo using a needle via the septum in the vial. Yield of the orange crystals 40.2 mg, 25%. Crystals suitable for single crystal X-ray analysis were obtained by layering diethyl ether over a THF solution at room temperature inside an argon glovebox. ^1^H NMR (500 MHz, C_6_D_6_) δ: 12.15 (br s), 9.83 (br s), 8.54 (br s), 7.47 (br s), 4.22 (br s), 2.84 (br s), 2.40 (br s), 2.15 (br s), 1.97 (br s), −5.52 (br s). Broad overlapping signals in certain regions prevented integration. Elem. Anal.: Due to the sensitivity of complex **5**, satisfactory elemental analysis could not be obtained.

### Representative Procedure for the Suzuki–Miyaura Coupling Reactions

To an oven-dried 25 mL Schlenk tube equipped with a PTFE-coated stir bar was added powdered anhydrous K_3_PO_4_ (4.5 equiv). The tube was sealed with a rubber septum and the contents flame-dried under dynamic vacuum on a Schlenk line. All other solid reagents were weighed outside the glovebox and added to the cooled tube: aryl dimethylsulfamate (1.0 equiv), the Ni­(II) precatalyst (**3e–3h**) (2.5 mol % or 5 mol %), and the arylboronic acid (2.5 equiv). The Schlenk tube was transferred to a nitrogen-filled glovebox and dry toluene (2.5 mL) added (The liquid reagents 1-chloronaphthalene (1.0 equiv) and phenyl dimethylsulfamate were added inside the glovebox). The Schlenk tube was then sealed with a rubber septum then transferred outside the glovebox, where a nitrogen balloon was inserted via the septum. The reaction mixture was heated at 60–80 °C in an oil bath, with stirring for 1–24 h. The tube was then cooled and the crude reaction mixture filtered using a Hirsch funnel through a pad of Celite and silica gel. The residue and Schlenk tube were rinsed with CHCl_3_. The filtrate was then transferred to a 50 mL round-bottom flask and the solvent removed in vacuo. The internal standard 1,3,5-trimethoxybenzene was added and the mixture analyzed by ^1^H NMR spectroscopy in CDCl_3_. NMR yields are reported as the average of at least two independent trials.

### Representative Procedure for the Reductive Cleavage Reactions

To a flame-dried 4″ reaction tube equipped with a PTFE-coated stir bar was added the aryl dimethylsulfamate or 1-chloronaphthalene (0.250 mmol, 1.0 equiv), KO*t*Bu (0.500 mmol, 2.0 equiv), Ni­(II) precatalyst **3a–3h** (2.5 mol %), and *i*PrOH (2.5 mL). The tube was sealed with a rubber septum, and the mixture sparged with *N*_2(g)_ for 5 min. A nitrogen balloon was inserted via the septum and the mixture stirred at 80 °C for 1 h in an oil bath. The mixture was cooled, diluted with DCM, then filtered via a Hirsch funnel through a layer of Celite and silica gel. The filtrate was transferred to a 50 mL round-bottom flask and the solvent removed on a rotary evaporator. To the crude reaction product was added 1,3,5-trimethoxybenzene as an internal standard and the sample analyzed by ^1^H NMR spectroscopy in CDCl_3_. Reported NMR yields are the average of at least two independent trials.

## Supplementary Material







## Notes

Complete experimental procedures, compound characterization data, computational details, and results (PDF)
Crystallographic data for **3e** (CCDC 2475783), **3f** (CCDC 2475784), **3g** (CCDC 2475785), **3h** (CCDC 2475786), and **5** (CCDC 2472042) (CIF)
Cartesian coordinates for the DFT optimized structure used to obtain the SOMO plot for complex **5** is included as a separate file (CIF)
