# Supplementary material for: Comparison of Alkyl-Bridged Bis(N-Heterocyclic Carbene) Nickel Precatalysts: Structure and Catalytic Activity in the Reductive Cleavage and Suzuki–Miyaura Reactions
Source: ACS Omega. 2025 Nov 17;10(47):57354–64. doi: 10.1021/acsomega.5c07647 (PMC12676342; doi:10.1021/acsomega.5c07647)
Supplement: Supplementary file 1 [file ao5c07647_si_001.pdf]

Supporting Information

Comparison of Alkyl-bridged Bis(N-heterocyclic carbene) Nickel Precatalysts: Structure and Catalytic Activity in the Reductive Cleavage and Suzuki-Miyaura Reactions

Claudia S. Zhang,† Eleanor C. Beams,† Abigail L. Moffett,† Chuyi Luo,† Colin D. McMillen,§ Anthony R. Chianese,‡ Kerry-Ann Green*,†

†Department of Chemistry, Williams College, Williamstown, MA, 01267, USA
§Department of Chemistry, Clemson University, Clemson, South Carolina 29634, USA
‡Department of Chemistry, Colgate University, Hamilton, New York 13346, USA

Department of Chemistry Williams College,
Williamstown, MA 01267, United States

*E-mail: kg14@williams.edu

Table of Contents for the Supporting Information

1.0 General Considerations, Materials, Instrumentation and Software S3

1.1 General Considerations and Materials S3

1.2 Instrumentation and Software S3

1.3 Abbreviations

2.0 Synthesis & Characterization of Ligand Precursors, Ni(II) complexes and substrates

2.1 Synthesis & Characterization of bisbenzimidazolium salts (**2e-2h**) S4

2.2 Synthesis & Characterization of Nickel(II) Complexes (**3e-3h**, **5**) S5

2.3 Synthesis of Aryl sulfamate substrate S8

3.0 Catalytic Studies

3.1 Attempted Ni-catalyzed C-N coupling of aryl sulfamates S9

3.2 Reductive Cleavage Reactions S9

3.2.1 Reductive cleavage Products S11

3.2.2 Chemoselective Ni-catalyzed reductive cleavage S11

3.3 Suzuki-Miyaura Coupling Reactions S12

3.3.1 SMC cross-coupled products (**4a-4e**) S14

4.0 Mechanistic Studies:

4.1 NMR tube experiment – precatalyst activation S15

4.2 Deuterium labeling experiments S16

4.3 Radical Trapping Experiments S17

4.4 Reactivity of bis(NHC)Ni^{I}Br (**5**) in the reductive cleavage S18

4.5 Reactivity of bis(NHC)Ni^{I}Br (**5**) in the SMC S19

5.0 Determination of σ-donating properties of bis(NHCs) by estimating the $^{1}J_{C\text{-}H}$ S20

6.0 Percent Buried Volume (%V_{Bur}) S21

Bite Angles for the bis(NHCs) and Dihedral Angles of **3a–3h** S22

7.0 UV-Vis Spectra of complexes **3e-3h** S22

8.0. IR Spectra for bisbenzimidazolium salts (**2e-2h**) S23

9.0. NMR Spectra for reported compounds S25

10.0. Single Crystal X-ray Data (**3e-3h**, **5**) S47

11.0 DFT Calculations – Spin Density plot of **5** S53

12.0 References S54

1.1 General Considerations and Materials. All air- and moisture-sensitive procedures were conducted using standard Schlenk techniques or in a nitrogen-filled glovebox. 1-Benzyl-1*H*-benzo[d]imidazole (**1a**)[1,2], 1-(cyclohexylmethyl)-1*H*-benzo[*d*]imidazole (**1b**)[3], 1-(naphthalen-2-ylmethyl)-1*H*-benzo[*d*]imidazole (**1c**)[2], 1-(2-fluorobenzyl)-1*H*-benzo[*d*]imidazole (**1d**), 1-naphthyl dimethylsulfamate, phenyl dimethylsulfamate, quinolin-6-yl dimethylsulfamate and 4-chloro-1-naphthyl dimethylsulfamate were prepared according to modified literature procedures.[4,5,6] Precatalysts **3a**−**3d** were synthesized according to the procedure reported in our previous study.[4] Nickel(II) bromide, zinc powder, isopropanol, *i*PrOH-2d_1, potassium phosphate tribasic, phenylboronic acid, 4-(trifluoromethyl)phenylboronic acid, 4-methoxyphenylboronic acid, galvinoxyl and butylated hydroxytoluene (BHT) were obtained from Sigma Aldrich. Potassium *tert*-butoxide and 2,2,6,6-tetramethyl-1-piperidinyloxy (TEMPO) were obtained from Oakwood Chemical. The solvent *i*PrOH-d_8 (+99%D) was obtained from Thermoscientific. $Ni(OAc)_2$ was dehydrated by heating $Ni(OAc)_2.4H_2O$ sourced from Alfa Aesar in a round bottom flask on a Schlenk line under dynamic vacuum at 125 °C for 8 h. Dry toluene was collected from a JC Meyer solvent purification system in a Straus flask and stored in the glovebox. All other chemicals including solvents were obtained commercially and used as received unless otherwise stated.

1.2 Instrumentation and Software. All NMR spectra were recorded on a Bruker Avance 500 MHz NMR spectrometer with operating frequencies for ^{1}H, ^{13}C and ^{19}F at 500 MHz, 125.78 MHz, and 470.56 MHz respectively. Chemical shifts (δ) are reported in ppm relative to the residual proteo solvent signal ($CDCl_3$: ^{1}H: δ = 7.26 ppm, ^{13}C: 77.16 ppm; DMSO-d_6: ^{1}H: δ = 2.50 ppm, ^{13}C: 39.52 ppm; *i*PrOH-d_8 ^{1}H: δ = 1.21 ppm).[7,8] The ^{19}F NMR spectra are unreferenced. NMR data were processed and analyzed using MNova 14.3.0. IR spectra were acquired on a Perkin Elmer FT-IR spectrometer Spectrum Two (UATR Two) with Spectrum 100 software version 10.03.09. **UV-Vis data** were acquired in dichloromethane using a Jasco V730 spectrophotometer in quartz cuvettes (path length = 1 cm). Flash column chromatography was conducted using Silicycle SiliaFlash P60 silica gel using glass columns with PTFE stopcocks. **Elemental analyses** were performed by Atlantic Microlab, Inc., Norcross, GA. **Gas Chromatography** was performed on an Agilent Technologies 7820A GC system with a 5977B MSD and using MassHunter Data Acquisition. High resolution mass spectra (HRMS) were obtained using an Agilent 6230B ToF-MS with a 1260 Series HPLC system. **Single crystal X-ray crystallographic** data for complexes **3e-3h** were collected at 100 K using a Bruker D8 Venture Duo diffractometer equipped with a Photon 2 detector (**3e-3f**) and a Bruker D8 Quest diffractometer equipped with a Photon 3 detector (**3h**). Data were collected, integrated (SAINT), and corrected for absorption (SADABS) within the Apex 3 software suite.[9] Structure solution by intrinsic phasing (SHELXT) and refinement on F^2 using full matrix least squares techniques (SHELXL) were accomplished using the SHELXTL software package.[10,11] The structure determination for complex **5** was performed on an Oxford Diffraction Gemini-R diffractometer, using Cu-Kα radiation at 110 K. Complete experimental details of the crystallographic refinements are provided in Section 10.0.

1.3 Abbreviations

bis(*NHC)* = bis(N-heterocyclic carbene)*; SMC* = Suzuki-Miyaura coupling*; i*PrOH = isopropanol, KO*t*Bu = potassium tert-butoxide, %V_{Bur} = buried volume; NMR = nuclear magnetic resonance spectroscopy; HRMS ESI = high resolution mass spectrometry; PTFE = poly(tetrafluoroethylene); Coupling constants (*J*) are reported in Hz as; s = singlet, d = doublet; t = triplet; br s = broad singlet, m = multiplet.

2.0 Synthesis and Characterization of bisbenzimidazolium salts and Ni(II) complexes

2.1 General Procedure for the synthesis of the bisbenzimidazolium salts (2e-2h)

All alkyl-bridged bisbenzimidazolium bromides were prepared from the direct reaction of the N-substituted benzimidazole and the corresponding dihaloalkane.[12]

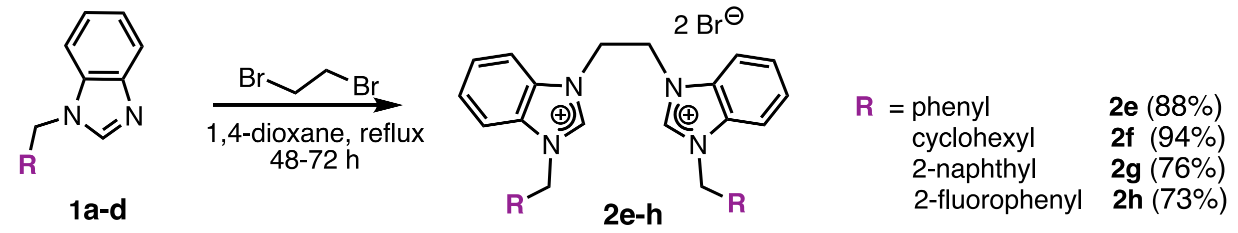

A Schlenk flask equipped with a PTFE-coated stir bar was charged with the *N*-substituted benzimidazole (**1a-1d**) (2.0-2.1 equiv.), 1,2-dibromoethane (1.0 equiv.) and 1,4-dioxane. The reaction flask was sealed, then evacuated and backfilled with nitrogen for three cycles. A nitrogen balloon was inserted via the septum and the mixture was heated at 100 °C for 20-72 h. A white precipitate formed over time and at the end of the heating period, the reaction mixture was filtered via a Hirsch funnel and the product rinsed with THF followed by diethyl ether. All bisbenzimidazolium salts were isolated as white powdery solids.

1,1′-Dibenzyl-3,3′-(1,2-ethanediyl)bisbenzimidazolium Dibromide (**2e**). The compound was synthesized from **1a** (995 mg, 4.78 mmol, 2.1 equiv.) and 1,2-dibromoethane (0.2 mL, 2.28 mmol, 1.0 equiv.) and 1,4-dioxane (6 mL). The mixture was heated at reflux for 72 h. Yield 1.217 g, (88%). **^{1}H NMR** (500 MHz, DMSO-d_6): δ 10.00 (s, 2H), 7.93 (d, J = 8.4 Hz, 2H), 7.85 (d, J = 8.4 Hz, 2H), 7.60 (t, J = 8.3 Hz, 2H), 7.49 – 7.38 (m, 12H), 5.76 (s, 4H), 5.20 (s, 4H). **^{13}C NMR** (126 MHz, DMSO-d_6): δ 143.2, 133.6, 131.2, 130.7, 129.0, 128.8, 128.34, 126.9, 126.8, 114.0, 113.1, 50.0, 45.9. The NMR data are consistent with those reported in the literature.[13]

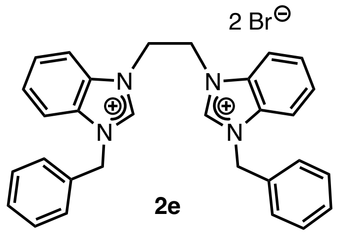

1,1′-Di(cyclohexylmethyl)-3,3′-(1,2-ethanediyl)bisbenzimidazolium Dibromide (**2f**). The compound was synthesized from **1b** (827 mg, 3.86 mmol, 2.1 equiv.) and 1,2-dibromoethane (0.16 mL, 1.84 mmol, 1.0 equiv.) and 1,4-dioxane (6 mL). The mixture was heated at reflux for 72 h. Yield 1.069 g, (94%). **^{1}H NMR** (500 MHz, DMSO-d_6): δ 9.83 (s, 2H), 8.12 (d, J = 8.3 Hz, 2H), 7.92 (d, J = 8.3 Hz, 2H), 7.68 (m, 2H), 7.61 (m, 2H), 5.16 (s, 4H), 4.30 (d, J = 7.2 Hz, 4H), 1.81 (m, 2H), 1.65 (m, 6H), 1.46 (m, 4H), 1.11 (m, 6H), 1.00 – 0.83 (m, 4H). **^{13}C NMR** (126 MHz, DMSO-d_6): δ 143.0, 131.4, 130.9, 126.9, 126.8, 114.0, 113.1, 52.2, 45.7, 37.1, 29.4, 25.5, 25.0. **Calcd** for $C_{30}H_{40}Br_2N_4.2.5H_2O$: C, 54.47; H, 6.86; N, 8.47. **Found**: C, 54.46; H, 6.78; N, 8.40.

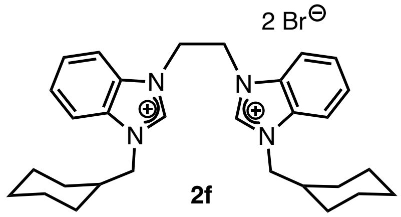

1,1′-Di(naphthalen-2-ylmethyl)-3,3′-(1,2-ethanediyl)bisbenzimidazolium Dibromide (2g). The compound was synthesized from **1c** (1.36 g, 5.25 mmol, 2.0 equiv.) and 1,2-dibromoethane (0.22 mL, 2.54 mmol, 1.0 equiv.) and 1,4-dioxane (8 mL). The mixture was heated at reflux for 48 h. Yield 1.35 g, (76%). **^{1}H NMR** (500 MHz, DMSO-d_6): δ 9.90 (s, 2H), 8.08 (s, 2H), 7.99 – 7.90 (m, 8H), 7.85 (d, J = 8.4 Hz, 2H), 7.62 – 7.53 (m, 6H), 7.46 (m, 4H), 5.88 (s, 4H), 5.17 (s, 4H). **^{13}C NMR** (126 MHz, DMSO-d_6): δ 143.3, 132.8, 132.7, 131.2, 131.0, 130.8, 128.8, 127.9, 127.8, 127.7, 126.90, 126.87, 126.81, 126.75, 125.6, 114.0, 113.1, 50.3, 45.9. **Calcd** for $C_{38}H_{32}Br_2N_4.0.5H_2O$: C, 63.97; H, 4.66; N, 7.85. **Found**: C, 63.69; H, 4.61; N, 7.89.

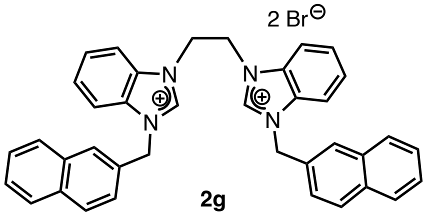

1,1′-Di-(2-fluorobenzyl)-3,3′-(1,2-ethanediyl)bisbenzimidazolium Dibromide (2h). The compound was synthesized from **1d** (3.24g, 14.3 mmol, 2.0 equiv.) and 1,2-dibromoethane (0.62 mL, 7.2 mmol, 1.0 equiv.) and 1,4-dioxane (12 mL). The mixture was heated at reflux for 72 h. Yield 3.23 g, (73%). **^{1}H NMR** (500 MHz, DMSO-d_6): δ 10.00 (s, 2H), 7.99 (d, J = 8.4 Hz, 2H), 7.90 (d, J = 8.4 Hz, 2H), 7.65-7.61 (m, 4H), 7.54 – 7.46 (m, 4H), 7.33 – 7.25 (m, 4H), 5.83 (s, 4H), 5.18 (s, 4H). **^{13}C NMR** (126 MHz, DMSO-d_6): δ 160.5 (d, J = 247.1 Hz), 143.4, 131.5 (d, J = 8.2 Hz), 131.2 (d, J = 2.8 Hz), 131.1, 130.7, 127.1, 126.8, 125.0 (d, J = 3.5 Hz), 120.7 (d, J = 14.2 Hz), 116.0 (d, J = 20.5 Hz), 113.7, 113.2, 45.8, 44.6 (d, J = 3.6 Hz). **^{19}F NMR** (471 MHz, DMSO-d_6): δ -116.47 (unreferenced). **Calcd** for $C_{30}H_{26}Br_2F_2N_4$: C, 56.27; H, 4.09; N, 8.75. **Found**: C, 56.08; H, 3.97; N, 8.73.

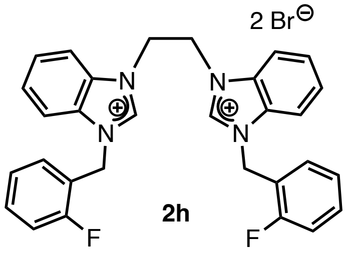

2.2 General Procedure for the synthesis of Nickel Complexes (3e-3h)

The well-defined nickel complexes (**3e-3h**) were prepared by a solvent-free synthesis.[12]

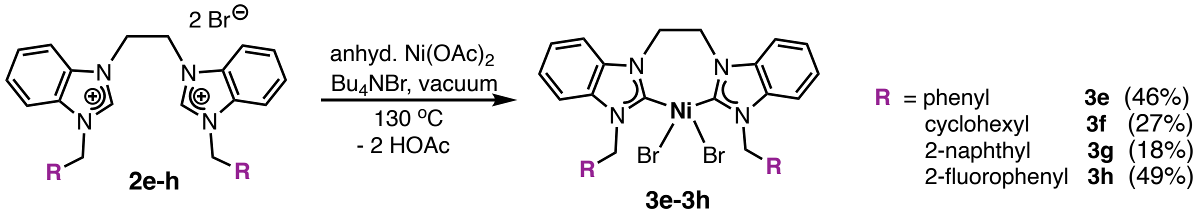

To a Schlenk flask, equipped with a PTFE-coated stir bar was added dehydrated $Ni(OAc)_2$ (1.0-1.1 equiv.), the bisbenzimidazolium salt (**2e-h**) (1.0 equiv.) and tetrabutylammonium bromide (4.3-8.0 equiv.). The flask was sealed with a rubber septum, connected to the Schlenk line, and placed in a preheated oil bath. The mixture was initially heated at 90 °C with stirring under dynamic vacuum for 30-45 min. The temperature was raised to 130 °C and the mixture heated with stirring under dynamic vacuum at this temperature for 4-7 h. The resulting viscous yellow-green slurry was cooled. Water was then added to dislodge the hardened solid with agitation from an ultrasonic bath. The yellow powder was isolated by vacuum filtration using a Hirsch funnel and the residue rinsed with copious amounts of water. The complex was further purified by flash column chromatography on silica gel by gradient elution (EtOAc: DCM 1:1.2 to 1:1).

Dibromido-1,1′-dibenzyl-3,3′-(1,2-ethanediyl)dibenzimidazolin-2,2′-diylidenenickel(II) (3e). The compound was synthesized from **2e** (1.623 g, 2.69 mmol, 1.0 equiv.) and $Ni(OAc)_2$ (0.513 g, 2.90 mmol, 1.1 equiv.) and tetrabutylammonium bromide (5.30 g, 16.4 mmol, 6.1 equiv.). The mixture was heated under vacuum for 5.5 h. Compound **3e** was an orange crystalline solid after column purification. Yield 816 mg, (46%). **^{1}H NMR** (500 MHz, DMSO-d_6) δ 7.76 (d, J = 8.2 Hz, 2H), 7.37 – 7.29 (m, 8H), 7.19 (t, J = 8.2 Hz, 2H), 7.11-7.06 (m, 6H), 6.38 (br s, 2H), 6.17 (d, J = 16.6 Hz, 2H), 5.55 (d, J = 15.6 Hz, 2H), 5.30 (d, J = 7.2 Hz, 2H). **^{13}C NMR** (126 MHz, DMSO-d_6) δ 135.9, 134.20, 134.15, 128.7, 127.9, 127.0, 123.6, 123.4, 111.1, 111.0, 51.0, 44.0. The Ni-$C_{carbene}$ signal was not observed. **Calcd** for $C_{30}H_{26}Br_2N_4Ni$: C, 54.51; H, 3.96; N, 8.48. **Found**: C, 54.95; H, 4.13; N, 8.43. Combustion analysis was high in carbon despite multiple attempts. Crystals suitable for single crystal X-ray diffraction analysis were obtained from the vapor diffusion of diethyl ether into an acetonitrile solution at ambient temperature.

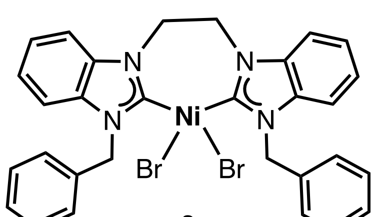

3e

Dibromido-1,1′-dicyclohexylmethyl-3,3′-(1,2-ethanediyl)dibenzimidazolin-2,2′-diylidenenickel(II) (3f). The compound was synthesized from **2f** (986 mg, 1.60 mmol, 1.0 equiv.) and $Ni(OAc)_2$ (283 mg, 1.60 mmol, 1.0 equiv.) and tetrabutylammonium bromide (3.0 g, 9.3 mmol, 5.8 equiv.). The mixture was heated under vacuum for 4 h. Compound **3f** was a yellow crystalline solid after column purification. Yield 292 mg, (27%). **^{1}H NMR** (500 MHz, DMSO-d_6): δ 7.71 – 7.67 (m, 4H), 7.33-7.27 (m, 4H), 6.44 (br s, 2H), 5.23 (br s, 2H), 4.74 (s, 4H), 2.44 (m, 2H), 1.67-1.47 (m, 10H), 1.23 – 1.18 (m, 10H). **^{13}C NMR** (126 MHz, DMSO-d_6): 135.0, 133.8, 123.4, 123.2, 111.4, 110.7, 54.0, 44.0, 38.1, 30.2, 25.7, 25.3. The Ni-$C_{carbene}$ signal was not observed. **Calcd** for $C_{30}H_{38}Br_2N_4Ni$: C, 53.53; H, 5.69; N, 8.32. **Found**: C, 53.99; H, 5.66; N, 8.11. Combustion analysis was high in carbon despite multiple attempts. Crystals suitable for single crystal X-ray diffraction analysis were obtained from the vapor diffusion of diethyl ether into an acetonitrile solution at ambient temperature.

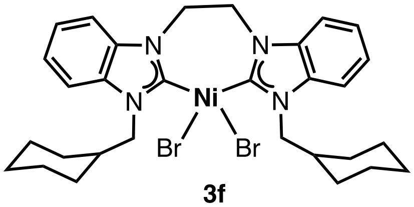

3f

Dibromido-1,1′-dinaphthalen-2-ylmethyl-3,3′-(1,2-ethanediyl)dibenzimidazolin-2,2′-diylidenenickel(II) (3g). The compound was synthesized from **2g** (412.5 mg, 0.586 mmol, 1.0 equiv.) and $Ni(OAc)_2$ (103.8 mg, 0.587 mmol, 1.0 equiv.) and tetrabutylammonium bromide (1.5 g, 4.7 mmol, 8.0 equiv.). The mixture was heated under vacuum for 6 h. Compound **3g** was a burnt orange crystalline solid after column purification. Yield 81 mg, (18%). **^{1}H NMR** (500 MHz, $CDCl_3$): δ 7.79 (d, J = 8.1 Hz, 2H), 7.62 (d, J = 8.4 Hz, 2H), 7.54 – 7.48 (m, 4H), 7.43 – 7.39 (m, 4H), 7.29 (s, 2H), 7.23 – 7.20 (m, 2H), 7.10 (d, J = 10.4 Hz, 2H), 7.07 – 7.02 (m, 2H), 6.81 (d, J = 8.4 Hz, 2H), 6.77 (m, 2H), 6.08 (d, J = 15.4 Hz, 2H), 5.43 (d, J = 15.9 Hz, 2H), 5.05 (m, 2H). **^{13}C NMR** (126 MHz, $CDCl_3$): δ 135.2, 134.6, 133.3, 133.1, 133.0, 128.8, 128.1, 127.9, 126.63, 126.61, 126.5, 125.4, 123.8, 123.6, 111.5, 109.3, 52.2, 44.1. The Ni-$C_{carbene}$ signal was not observed. **Calcd** for $C_{38}H_{30}Br_2N_4Ni$: C, 59.96; H, 3.97; N, 7.36. Found: C, 60.07; H, 3.87; N, 7.27. Crystals suitable for single crystal X-ray diffraction analysis were obtained from liquid-liquid diffusion of diethyl ether into an acetonitrile solution at ambient temperature.

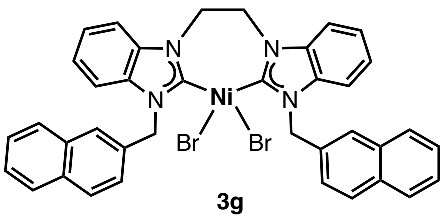

3g

Dibromido-1,1′-di-(2-fluorobenzyl)-3,3′-(1,2-ethanediyl)dibenzimidazolin-2,2′-diylidenenickel(II) (3h). The compound was synthesized from **2h** (1.0260 g, 1.60 mmol, 1.0 equiv.) and $Ni(OAc)_2$ (288.1 mg, 1.63 mmol, 1.0 equiv.) and tetrabutylammonium bromide (2.2 g, 6.9 mmol, 4.3 equiv.). The mixture was heated under vacuum for 4 h. Compound **3h** was a dark orange crystalline solid after column purification. Yield 546 mg, (49%). **^{1}H NMR** (500 MHz, DMSO-d_6) δ 7.79 (d, J = 8.2 Hz, 2H), 7.42 – 7.33 (m, 4H), 7.31-7.27 (m, 2H), 7.25 – 7.20 (m, 4H), 6.97-6.94 (m, 2H), 6.70-6.68 (m, 2H), 6.38 (s, 2H), 6.13 – 5.98 (m, 2H), 5.75 (m, 2H), 5.31 (m, 2H). **^{13}C NMR** (126 MHz, DMSO-d_6): δ 159.8 (d, J = 246.1 Hz), 134.1 (d, J = 2.7 Hz), 130.0 (d, J = 8.2 Hz), 128.8 (d, J = 3.6 Hz), 124.4 (d, J = 3.2 Hz), 123.8, 123.6, 122.9 (d, J = 13.6 Hz), 115.6 (d, J = 20.4 Hz), 111.1, 110.8, 45.1 (d, J = 5.0 Hz), 44.1. The Ni-$C_{carbene}$ signal was not observed. **^{19}F NMR** (471 MHz, DMSO-d_6): δ -117.28 (unreferenced). **Calcd** for $C_{30}H_{24}Br_2F_2N_4Ni$: C, 51.69; H, 3.47; N, 8.04. **Found**: C, 51.92; H, 3.37; N, 7.99. Crystals suitable for single crystal X-ray diffraction analysis were obtained by layering diethyl ether over a chloroform solution at ambient temperature.

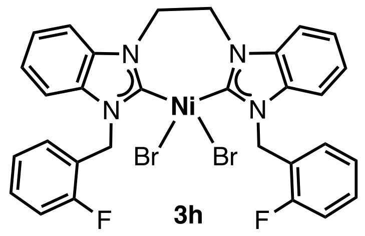

Synthesis of bis(NHC)Ni^{I}Br (5) by Zn reduction

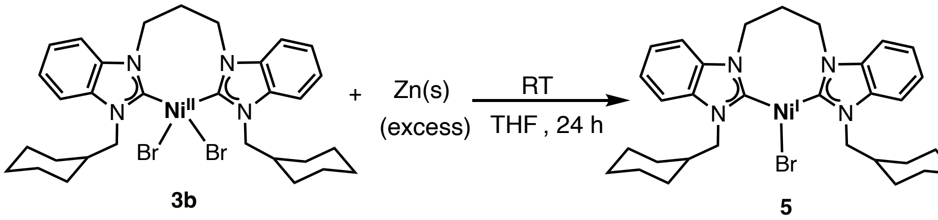

Inside a nitrogen-filled glovebox, an oven-dried 20 mL scintillation vial equipped with a Teflon coated stir bar was charged with **3b** (183.3 mg, 0.27 mmol), zinc powder (878.9 mg, 13.4 mmol, 50 equiv.) and THF (6 mL). The mixture was allowed to stir vigorously at RT for 24 h. The resulting dark brown mixture with unreacted zinc was filtered using 0.45 μm syringe filter followed by a second filtration via a layer of Celite in a pipet plugged with cotton. The dark brown solution was concentrated *in vacuo*, after which pentane (6 mL) was added to precipitate the solid. The product was isolated by vacuum filtration via a sintered glass funnel and the solid rinsed with pentane. Recrystallization: The dark green solid sample was taken in THF (4 mL) in a scintillation vial and layered with Et_2O (5 mL) then placed in the freezer at -30 °C for 48 h. The dark upper layer was decanted, using a pipet. A fresh batch of Et_2O (5 mL) was added to the sample and decantation repeated. A bright orange-yellow crystalline solid was obtained. Residual Et_2O was removed *in vacuo* using a needle via the septum in the vial. Yield of the orange crystals 40.2 mg, 25%. Crystals suitable for single crystal X-ray analysis were obtained by layering diethyl ether over a THF solution at room temperature inside an argon glovebox. **^{1}H NMR** (500 MHz, C_6D_6) δ 12.15 (br s), 9.83 (br s), 8.54 (br s), 7.47 (br s), 4.22 (br s), 2.84 (br s), 2.40 (br s), 2.15 (br s), 1.97 (br s), -5.52 (br s). Broad overlapping signals in certain regions prevented integration. **Elem. Anal.:** Due to the sensitivity of complex **5**, satisfactory elemental analysis could not be obtained.

2.3 Synthesis of 4-chloro-1-naphthyl dimethylsulfamate.[5]

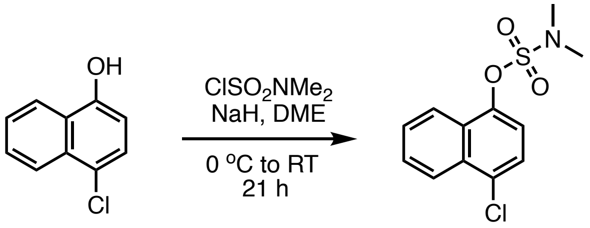

An oven-dried 100 mL round bottom flask equipped with a PTFE-coated stir bar was charged with NaH (0.5396 g, 13.5 mmol, 1.2 equiv., 60% dispersion in oil). The flask was sealed with a septum then flushed with nitrogen gas for about 5 min and cooled in an ice bath to 0 ºC. A solution of the 4-chloro-1-naphthol (2.0039 g, 11.2 mmol) in DME (15 mL) was slowly transferred to the cooled NaH in the reaction flask via cannula with stirring. The resulting mixture was warmed up to room temperature, then cooled back to 0 °C in an ice bath. A solution of N,N-dimethylsulfamoyl chloride (1.4 mL, 13.04 mmol) in DME (5 mL) was transferred via cannula to the mixture in the reaction flask with stirring. A nitrogen balloon was secured to the reaction flask, and the mixture stirred at room temperature for 21 h. The reaction was quenched with water (5 mL), and the organic solvent removed using a rotary evaporator. **Work-up**: The reaction mixture was transferred to a 125 mL separatory funnel with diethyl ether (50 mL) and water (15 mL), and the organic layer was extracted. The separated organic layer was washed with 1 M KOH (15 mL) followed by water (15 mL). The combined aqueous layers were transferred to the separatory funnel and extracted with diethyl ether (3 x 20 mL). The combined organic layers were washed with brine (15 mL) then dried over Na_2SO_4. The solvent was removed using a rotary evaporator and an orange-red liquid was obtained as the crude mixture. Purification by column chromatography with a solvent system of hexanes:EtOAc (6:1) yielded the desired compound as an off-white crystalline solid (1.94 g, 61%). **^{1}H NMR** (500 MHz, $CDCl_3$): δ 8.29 (d, J = 6.7 Hz, 1H), 8.20 (d, J = 6.6 Hz, 1H), 7.71 – 7.61 (m, 2H), 7.56 (d, J = 6.7 Hz, 1H), 7.48 (d, J = 8.2 Hz, 1H), 3.09 (s, 6H). **^{13}C NMR** (126 MHz, $CDCl_3$): δ 145.1, 131.9, 130.2, 128.3, 128.0, 127.7, 125.7, 125.0, 122.1, 39.06. **Calcd.** for $C_{12}H_{12}ClNO_3S$: C, 50.44; H, 4.23; 4.90. **Found**: C, 50.40; H, 4.18; N, 4.89. **HRMS (LC-TOF)**: Calculated for $C_{12}H_{12}ClNO_3SNa^+$ ([M + Na]$^+$): 308.0124; found 308.0134.

3.0 Catalytic Studies

3.1 Attempted Ni-catalyzed C-N Coupling of Aryl Sulfamates

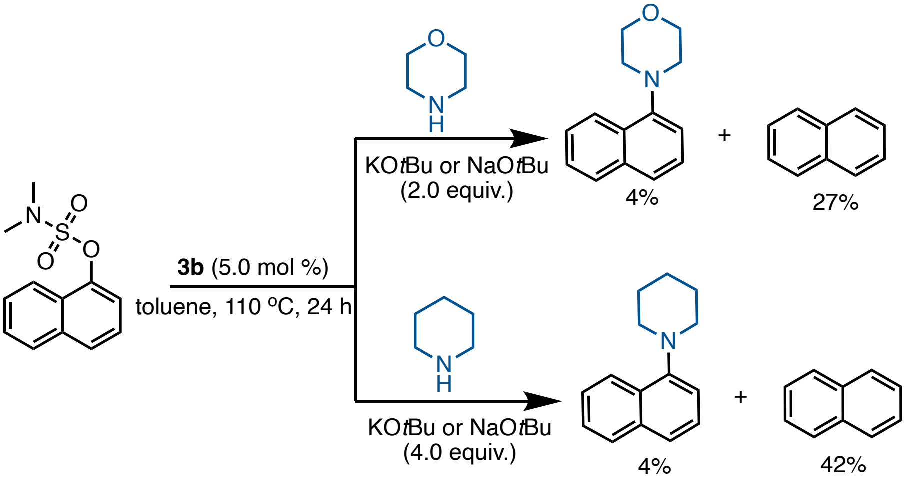

Representative Procedure for the Ni-catalyzed C-N Coupling. To a flame dried 4" reaction tube equipped with a PTFE-coated stir bar was added 1-naphthyl dimethylsulfamate (1.0 equiv.), amine (2.4-3.6 equiv.), base (2.0-4.0 equiv.), Ni(II) precatalyst (**3b**, 5 mol %), and dry toluene (2.5 mL). The tube was sealed with a rubber septum, and the contents sparged with N_2(g) for 5 minutes. A nitrogen balloon was then inserted via the septum and the mixture stirred at 110 °C for 24 h in an oil bath. The mixture was cooled, diluted with DCM, then filtered through two layers of filter paper, a layer of Celite, and a layer of silica gel. The tube and residue on the filter paper were rinsed with DCM, and the filtrate was transferred to a 50 mL round bottom flask. The solvent was then removed on a rotary evaporator. To the crude product was added 1,3,5-trimethoxybenzene as an internal standard. The sample was dissolved in $CDCl_3$ and analyzed by ^{1}H NMR spectroscopy and further for GC-MS analysis. **Note:** The product of reductive cleavage was only observed when tert-butoxide bases were employed. In the cases of K_3PO_4, TEA or DBU only trace quantities of the reduced product or no reduced product was observed.

3.2 Reductive Cleavage Reactions

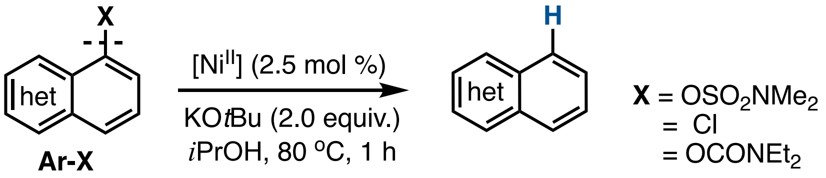

Representative Procedure for the Reductive Cleavage Reactions. To a flame dried 4" reaction tube equipped with a PTFE-coated stir bar was added the aryl dimethylsulfamate or 1-chloronaphthalene (0.250 mmol, 1.0 equiv.), KO*t*Bu (0.500 mmol, 2.0 equiv.), Ni(II) precatalyst **3a–3h** (2.5 mol %), and *i*PrOH (2.5 mL). The tube was sealed with a rubber septum, and the mixture sparged with N_2(g) for 5 min. A nitrogen balloon was inserted via the septum and the mixture stirred at 80 °C for 1 h in an oil bath. The mixture was cooled, diluted with DCM, then filtered via a Hirsch funnel through a layer of Celite and silica gel. The filtrate was transferred to a 50 mL round bottom flask and the solvent removed on a rotary evaporator. To the crude reaction product was added 1,3,5-trimethoxybenzene as an internal standard and the sample analyzed by ^{1}H NMR spectroscopy in $CDCl_3$. Reported NMR yields are the average of at least two independent trials.

Table S1. Optimization of the reductive cleavage reaction

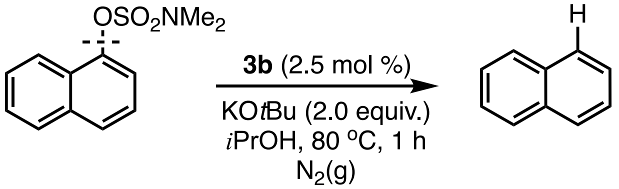

Entry	Deviation from standard conditions	Yield (%)[a]
1	none	>99
2	50 °C	87
3	room temp (25 °C)	0
4	without Ni precatalyst	0
5	$NiBr_2$ (2.5 mol %) instead of **3b**	0
6	**3b** (5.0 mol %), *i*PrOH: toluene (1:10), 24 h, 80 °C	90*
7	KO*t*Bu (1.0 equiv.)	89
8	without KO*t*Bu	0
9	in air	0

[a]Standard conditions: **3b** (2.5 mol %), 1-naphthyl dimethylsulfamate (1 equiv.), KO*t*Bu (2.0 equiv.), *i*PrOH (2.5 mL) under N_2(g) at 80 °C for 1 h. Yields were determined by ^{1}H NMR analysis with 1,3,5-trimethoxybenzene as an internal standard and are the average of two independent trials. *Yield determined from a single trial.

Table S2. Investigation of Ni-catalyzed Reductive Cleavage of Phenol-derived Substrates and 1-Chloronaphthalene with **3a–3h***[a]*

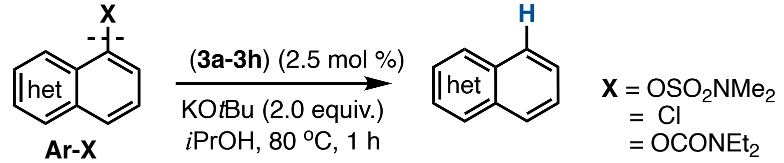

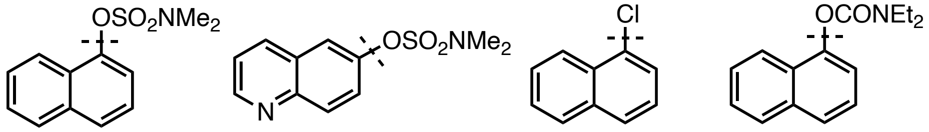

**3a**, 88%	**3a**, 95%	**3a**, 98%	**3a**, --
**3b**, >99%(98)	**3b**, 89%	**3b**, >99%	**3b**, 0%
**3c**, 93%	**3c**, >99%	**3c**, 90%	**3c**, --
**3d**, 87%	**3d**, 99%	**3d**, 97%	**3d**, 0%
**3e**, 94%	**3e**, 77%	**3e**, >99%	**3e**, --
**3f**, 68%	**3f**, 99%	**3f**, 98%	**3f**, 0%
**3g**, 65%	**3g**, 55%	**3g**, 97%	**3g**, --
**3h**, 89%	**3h**, >99%	**3h**, 98%	**3h**, 0%

[a]Reaction conditions: (**3a–3h**) (2.5 mol %), **Ar-X** (1 equiv.), KO*t*Bu (2.0 equiv.), *i*PrOH (2.5 mL), under $N_{2(g)}$ at 80 °C for 1 h. Yields listed below each starting material were determined by ^{1}H NMR analysis with 1,3,5-trimethoxybenzene as an internal standard and are the average of two independent trials. For **X** = $OCONEt_2$, naphthalene was not detected for **3b**, **3d**, **3f**, and **3h** and the 1-naphthol side product was observed in 0-36% yield. For **X** = $OCONEt_2$, table entries are shown as -- for **3a**, **3c**, **3e**, and **3g** as the reductive cleavage was not investigated for these precatalysts. Isolated yield in parentheses.

3.2.1 Reductive Cleavage Products

From 1-naphthyl dimethylsulfamate:

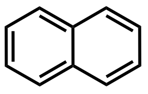

Naphthalene was prepared according to the general procedure with the representative quantities: 1-naphthyl dimethylsulfamate (64.5 mg, 0.257 mmol, 1.0 equiv.), KO*t*Bu (58.9 mg, 0.524 mmol, 2.0 equiv.), precatalysts **3a–3h** (0.00639 mmol, 2.5 mol %) and 1,3,5-trimethoxybenzene (7.5 mg, 0.0446 mmol). The average NMR yields were 88% (**3a**), >99% (**3b**), 93% (**3c**), 87% (**3d**) 94% (**3e**), 68% (**3f**), 65% (**3g**) and 89% (**3h**). **^{1}H NMR** (500 MHz, $CDCl_3$) δ 7.85 (m, 4H), 7.49 (m, 4H). GC-MS analysis confirmed the naphthalene product. The NMR data are consistent with those reported in the literature.[14,15]

From 1-chloronaphthalene:

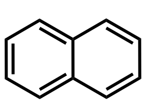

Naphthalene was prepared according to the general procedure with the representative quantities: 1-chloronaphthalene (40 µL, 0.295 mmol, 1.0 equiv.), KO*t*Bu (65.0 mg, 0.578 mmol, 2.0 equiv.), precatalysts **3a–3h** (0.00639 mmol, 2.2 mol %) and 1,3,5-trimethoxybenzene (7.5 mg, 0.0386 mmol). The average NMR yields were 98% (**3a**), >99% (**3b**), 90% (**3c**), 97% (**3d**) >99% (**3e**), 98% (**3f**), 97% (**3g**) and 98% (**3h**). GC-MS analysis confirmed the naphthalene product. The NMR data are consistent with those reported in the literature.[14,15]

From quinolin-6-yl dimethylsulfamate:

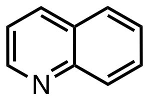

Quinoline was prepared according to the general procedure with the representative quantities: 6-quinolinyl dimethylsulfamate (63.3 mg, 0.251 mmol, 1.0 equiv.), KO*t*Bu (56.1 mg, 0.500 mmol, 2.0 equiv.), and precatalysts **3a–3h** (0.00610 mmol, 2.4 mol %) and 1,3,5-trimethoxybenzene (6.7 mg, 0.0386 mmol). The average NMR yields were 95% (**3a**), 89% (**3b**), >99% (**3c**), 99% (**3d**) 77% (**3e**), 99% (**3f**), 55% (**3g**) and >99% (**3h**). GC-MS analysis confirmed the quinoline product. The NMR data are consistent with those reported in the literature.[15]

3.2.2 Investigation of Chemoselective Ni-catalyzed Reductive Cleavage

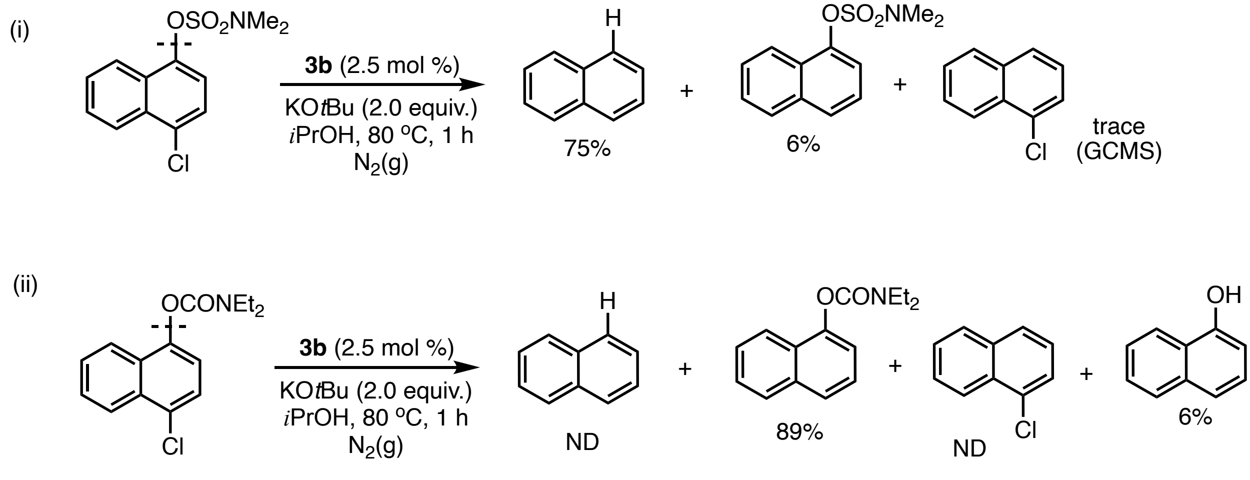

ND = not detected

Scheme S1. Investigation of the chemoselective Ni-catalyzed reductive cleavage using precatalyst **3b** with (i) 4-chloro-1-naphthyl dimethylsulfamate and (ii) 4-chloro-1-naphthyl *N*,*N*-diethylcarbamate.

3.3 Suzuki-Miyaura Coupling Reaction

Representative Procedure. To an oven-dried 25 mL Schlenk tube equipped with a PTFE-coated stir bar was added powdered anhydrous K_3PO_4 (4.5 equiv.). The tube was sealed with a rubber septum and the contents flame-dried under dynamic vacuum on a Schlenk line. All other solid reagents were weighed outside the glovebox and added to the cooled tube: aryl dimethylsulfamate (1.0 equiv.), the Ni(II) precatalyst (**3e–3h**) (2.5 mol % or 5 mol %), and the arylboronic acid (2.5 equiv.). The Schlenk tube was transferred to a nitrogen-filled glovebox and dry toluene (2.5 mL) added. (The liquid reagents 1-chloronaphthalene (1.0 equiv.) and phenyl dimethylsulfamate were added inside the glovebox). The Schlenk tube was then sealed with a rubber septum then transferred outside the glovebox, where a nitrogen balloon was inserted via the septum. The reaction mixture was heated at 60-80 °C in an oil bath, with stirring for 1-24 h. The tube was then cooled and the crude reaction mixture filtered using a Hirsch funnel through a pad of Celite and silica gel. The residue and Schlenk tube were rinsed with $CHCl_3$. The filtrate was then transferred to a 50 mL round bottom flask and the solvent removed in vacuo. The internal standard 1,3,5-trimethoxybenzene was added and the mixture analyzed by ^1H NMR spectroscopy in $CDCl_3$. NMR yields are reported as the average of at least two independent trials.

Table S3. NMR Yields (%) for the Suzuki-Miyaura Coupling of 1-naphthyl dimethylsulfamate with aryl boronic acids using bis(NHC)$NiBr_2$ precatalysts **3a–3h**[a]

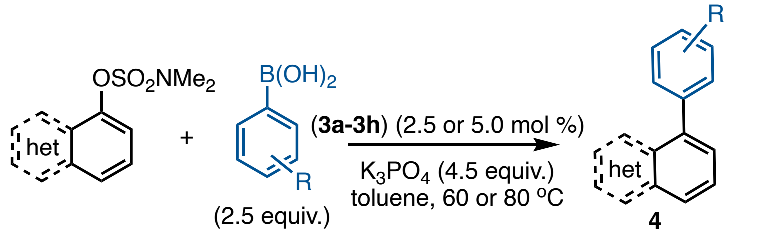

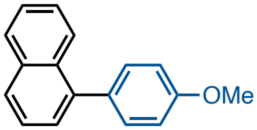

4a
3a, 72%[b]
3b, 93%[b]
3c, 76%[b]
3d, 80%[b]
3e, 26% (69%)[c]
3f, 85% (99%)[c]
3g, 49% (61%)[c]
3h, 41% (69%)[c]

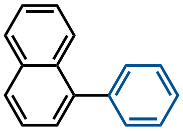

4b
3a, 41%[b]
3b, 84%[b]
3c, 38%[b]
3d, 58%[b]
3e, 0%
3f, 21%
3g, 0%
3h, 33%

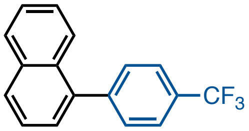

4c
3a, 50%[b]
3b, 64%[b]
3c, 36%[b]
3d, 31%[b]
3e, 0%
3f, 13%
3g, 0%
3h, 22%

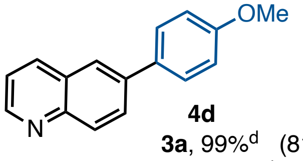

4d
3a, 99%[d] (81%)[b*]
3b, >99%[d] (99%)[b*]
3c, >99%[d] (46%)[b*]
3d, 99%[d] (89%)[b*]
3e, 51%[d]
3f, 97%[d]
3g, 20%[d]
3h, 99%[d] (89%)[*]

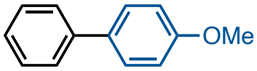

4e
3a, 43%[b,e]
3b, 59%[b,e]
3c, 36%[b,e]
3d, 31%[b,e]
3e, 12%[e]
3f, 16%[e]
3g, 39%[e]
3h, 21%[e]

[a]Reaction conditions: (**3a–3h**) (2.5 mol %), aryl sulfamate (1 equiv.), aryl boronic acid (2.5 equiv.), K_3PO_4 (4.5 equiv.), toluene (2.5 mL), 4 h at 60 °C. Yields listed below each starting material were determined by ^{1}H NMR analysis with 1,3,5-trimethoxybenzene as an internal standard and are the average of two independent trials. [b]Yields for **3a–3d** were reported in a prior study.[4] [*]time = 1 h. [c]**3e–3h** (5.0 mol %). [d]80 °C, 24 h. [e]80 °C, 16 h.

3.3.1 SMC Cross-coupled Products 4a–4e

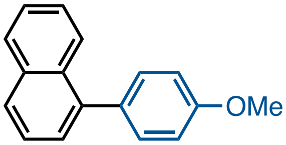

4a

Compound **4a** was prepared according to the general procedure with the representative quantities: K_3PO_4 (248.7 mg, 1.17 mmol, 4.5 equiv.), 1-naphthyl dimethylsulfamate (64.8 mg, 0.258 mmol, 1.0 equiv.), **3e–3h** (0.0065 mmol, 2.5 mol %), 4-methoxyphenylboronic acid (103.4 mg, 0.646 mmol, 2.5 equiv.), dry toluene (2.5 mL) and the reaction mixtures stirred at 60 °C for 4 h. The average NMR yields were 26% (**3e**), 85% (**3f**), 49% (**3g**) and 41% (**3h**). **^{1}H NMR** (500 MHz, $CDCl_3$): δ 7.95 – 7.87 (m, 2H), 7.84 (d, *J* = 8.2 Hz, 1H), 7.54 – 7.46 (m, 2H), 7.46 – 7.38 (m, 4H), 7.07 – 7.00 (m, 2H), 3.90 (s, 3H). The NMR data are consistent with those reported in the literature.[5,16]

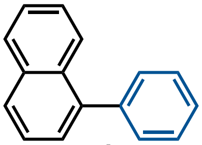

4b

Compound **4b** was prepared according to the general procedure with the representative quantities: K_3PO_4 (248.9 mg, 1.17 mmol, 4.5 equiv.), 1-naphthyl dimethylsulfamate (64.8 mg, 0.258 mmol, 1.0 equiv.), **3e–3h** (0.0065 mmol, 2.5 mol %), phenylboronic acid (82.6 mg, 0.657 mmol, 2.5 equiv.), dry toluene (2.5 mL) and the reaction mixtures stirred at 60 °C for 4 h. The average NMR yields were 0% (**3e**), 21% (**3f**), 0% (**3g**) and 33% (**3h**). **^{1}H NMR** (500 MHz, $CDCl_3$): δ 7.93 – 7.89 (m, 2H), 7.87 (d, *J* = 8.2 Hz, 1H), 7.56 – 7.47 (m, 6H), 7.47 – 7.41 (m, 3H). The NMR data are consistent with those reported in the literature.[17,18]

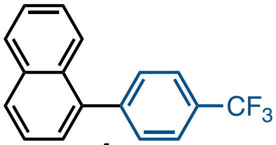

4c

Compound **4c** was prepared according to the general procedure with the representative quantities: K_3PO_4 (249.0 mg, 1.17 mmol, 4.5 equiv.), 1-naphthyl dimethylsulfamate (64.8 mg, 0.258 mmol, 1.0 equiv.), **3e–3h** (0.0065 mmol, 2.5 mol %), 4-trifluoromethyphenylboronic acid (128.6 mg, 0.643 mmol, 2.5 equiv.), dry toluene (2.5 mL) and the reaction mixture was stirred at 60 °C for 4 h. The average NMR yields were 0% (**3e**), 13% (**3f**), 0% (**3g**) and 22% (**3h**). **^{1}H NMR** (500 MHz, $CDCl_3$): δ 7.93 (dd, *J* = 12.1, 8.2 Hz, 2H), 7.82 (d, *J* = 8.4 Hz, 1H), 7.77 (m, 2H), 7.65 – 7.60 (d, *J* = 8.3 Hz, 2H), 7.58 – 7.50 (m, 2H), 7.47 (m, 1H), 7.43 (dd, *J* = 7.0, 1.2 Hz, 1H). The NMR data are consistent with those reported in the literature.[19,20]

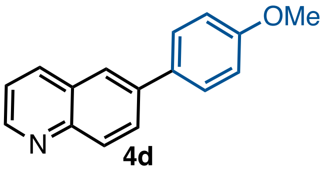

4d

Compound **4d** was prepared according to the general procedure with the representative quantities: K_3PO_4 (249.0 mg, 1.17 mmol, 4.5 equiv.), quinolin-6-yl dimethylsulfamate (65.1 mg, 0.258 mmol, 1.0 equiv.), **3e–3h** (0.0065 mmol, 2.5 mol %), 4-methoxyphenylboronic acid (103.4 mg, 0.646 mmol, 2.5 equiv.), dry toluene (2.5 mL) and the reaction mixtures were stirred at 80 °C for 24 h. The average NMR yields were 51% (**3e**), 97% (**3f**), 20% (**3g**) and 99% (**3h**). **^{1}H NMR** (500 MHz, $CDCl_3$): δ 8.90 (d, *J* = 3.8 Hz, 1H), 8.22 (d, *J* = 8.1 Hz, 1H), 8.18 (d, *J* = 8.7 Hz, 1H), 7.97 (d, *J* = 9.5 Hz, 2H), 7.67 (d, *J* = 4.3 Hz, 2H), 7.43 (dd, *J* = 8.4, 3.8 Hz, 1H), 7.04 (d, *J* = 6.6 Hz, 2H), 3.89 (s, 3H). The NMR data are consistent with those reported in the literature.[16,17]

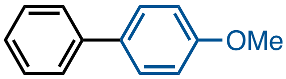

4e

Compound **4e** was prepared according to the general procedure with the quantities: K_3PO_4 (246.2 mg, 1.16 mmol, 4.3 equiv.), phenyl dimethylsulfamate (54.0 mg, 0.268 mmol, 1.0 equiv.), **3e–3h** (0.0064 mmol, 2.4 mol %), 4-methoxyphenylboronic acid (103.4 mg, 0.651 mmol, 2.4 equiv.), dry toluene (2.5 mL) and the reaction mixtures were stirred at 80 °C for 16 h. The average NMR yields were 12% (**3e**), 16% (**3f**), 39% (**3g**) and 21% (**3h**).**^{1}H NMR** (500 MHz, $CDCl_3$): δ 7.58 – 7.49 (m, 4H), 7.45 – 7.38 (m, 2H), 7.33 – 7.28 (m, 1H), 7.03 – 6.95 (m, 2H), 3.86 (s, 3H). The NMR data are consistent with those reported in the literature.[5,16,19]

4.0 Mechanistic Studies

4.1 Precatalyst Activation – NMR Tube Experiment

Precatalyst **3b** (2.4 mg, 3.49 x 10^{-3} mmol) and KO*t*Bu (29.8 mg, 0.266 mmol, 76.0 equiv.) were added to a J. Young NMR tube. The NMR tube was transferred to a nitrogen-filled glovebox and *i*PrOH-d_8 (0.6 mL) added. The pale yellow mixture was shaken vigorously and a ^{1}H NMR spectrum immediately acquired. The NMR tube was then heated at 80 °C for 1 h in an oil bath. Within ~4 min of heating the mixture became an intense brown color. After the 1 h had elapsed, another ^{1}H NMR spectrum was acquired.

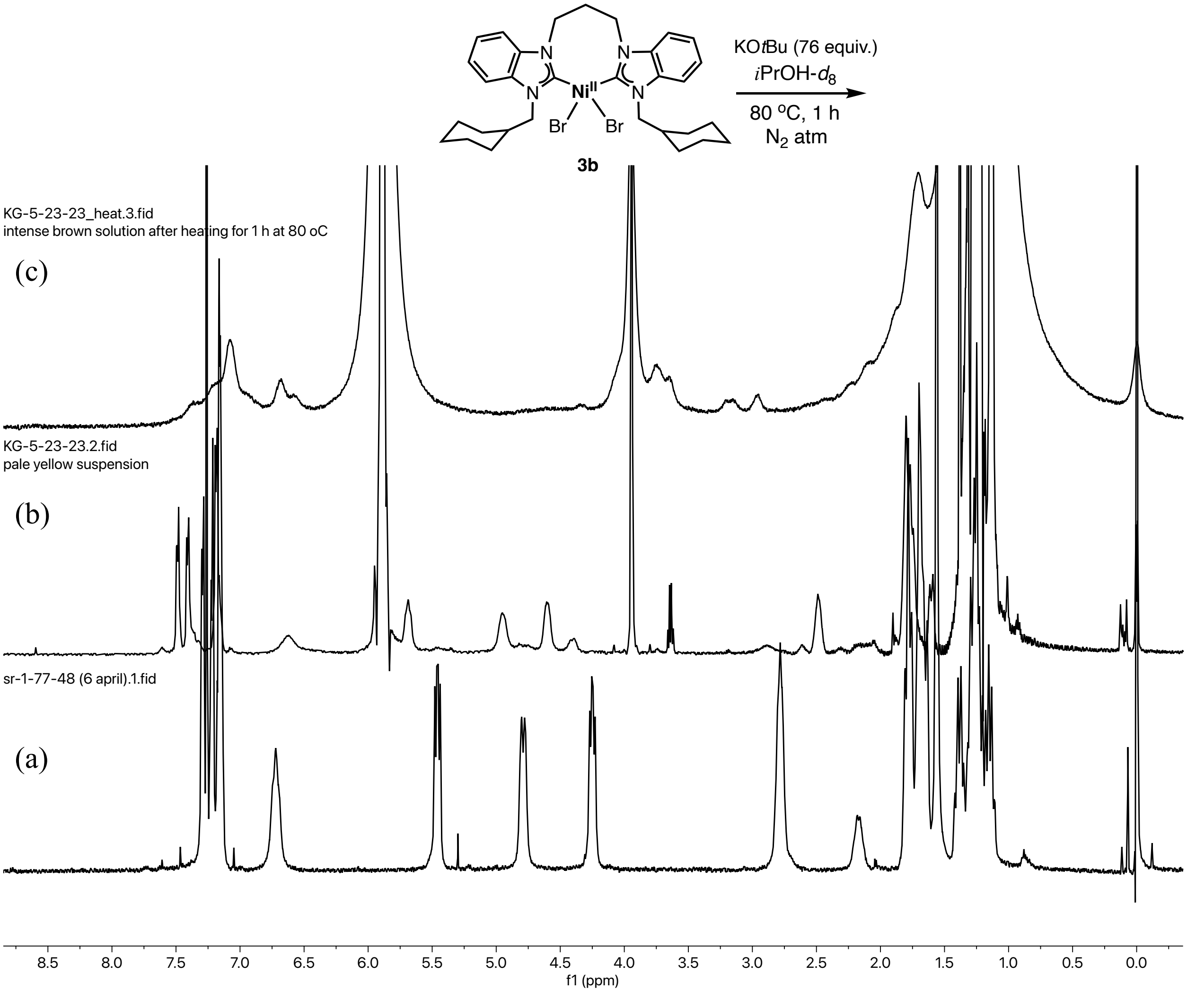

Figure S1: Stacked NMR plot (a) **3b** in $CDCl_3$ at RT, (b) pale yellow mixture of **3b**, KO*t*Bu in *i*PrOH-d_8 before heating, (c) intense brown mixture of **3b**, KO*t*Bu in *i*PrOH-d_8 after heating at 80 °C for 1 h.

4.2 Deuterium Labeling Experiments

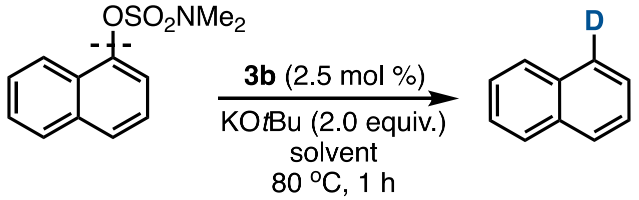

solvent	yield (%)	aryl sulfamate recovery (%)
*i*PrOH-2d_1	56	33
*i*PrOH-2d_1: toluene (1:10)	17	82

Representative Procedure. To a flame dried 4" reaction tube equipped with a PTFE-coated stir bar was added 1-naphthyl dimethylsulfamate (1.0 equiv.), KO*t*Bu (2.0 equiv.), **3b** (2.5 mol %), and solvent (1.25 – 2.75 mL). The tube was sealed with a rubber septum, and the mixture sparged with N_2(g) for 5 min. A nitrogen balloon was inserted via the septum and the mixture stirred at 80 °C for 1 h in an oil bath. The mixture was cooled, and the crude sample diluted with DCM, then filtered via a Hirsch funnel through a layer of Celite and silica gel. The filtrate was transferred to a 50 mL round bottom flask and the solvent removed on a rotary evaporator. To the crude reaction product was added 1,3,5-trimethoxybenzene (5.0-7.0 mg) as an internal standard and the sample analyzed by ^{1}H NMR spectroscopy in $CDCl_3$. The product of reductive cleavage and the recovered starting material were quantified by comparing the integration ratios of selected signals of the product and the starting material to that of the internal standard. Reported NMR yields are an average of two independent trials. The average NMR yield of naphthalene-1d in the case of neat *i*PrOH-2d_1 is 56% and the average recovery of the 1-naphthyl dimethylsulfamate was 33%. The average NMR yield of naphthalene-1d in the case of the *i*PrOH-2d_1: toluene (1:10) mixture was 17% and the average recovery of the 1-naphthyl dimethylsulfamate is 82%. An aliquot of the sample solution was also analyzed by GC-MS. The %D content was determined using the peak at δ 7.86-7.84 (including a deuterated site) and the peak at δ 7.49-7.47.[21]

4.3 Radical Trapping Experiments

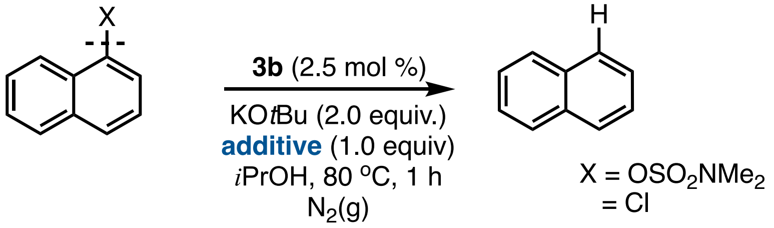

Entry	X	additive	yield (%)[a]	1-naphthyl dimethylsulfamate recovery(%)
1	OSO_2NMe_2	none	>99	--
2		TEMPO	0	>99
3		galvinoxyl	0	>99
4		BHT	0	>99
5	Cl	none	>99	--
6		TEMPO	5	94

[a]Reaction conditions: **3b** (2.5 mol %), 1-naphthyl dimethylsulfamate or 1-chloronaphthalene (1 equiv.), KO*t*Bu (2.0 equiv.), *i*PrOH (2.5 mL), additive (1.0 equiv.) under N_2(g) at 80 °C for 1 h. Yields were determined by ^{1}H NMR and are the average of two independent trials.

Representative Procedure. To a flame dried 4" reaction tube equipped with a PTFE-coated stir bar was added 1-naphthyl dimethylsulfamate or 1-chloronaphthalene (0.250 mmol, 1.0 equiv.), KO*t*Bu (2.0 equiv.), **3b** (2.5 mol %), additive (0.250 mmol, 1.0 equiv.), and *i*PrOH (1.25 – 2.75 mL). The tube was sealed with a rubber septum, and the mixture sparged with N_2(g) for 5 min. A nitrogen balloon was inserted via the septum and the mixture stirred at 80 °C for 1 h in an oil bath. The mixture was cooled, and the crude sample diluted with DCM, then filtered via a Hirsch funnel through a layer of Celite and silica gel. The filtrate was transferred to a 50 mL round bottom flask and the solvent removed on a rotary evaporator. To the crude reaction product was added 1,3,5-trimethoxybenzene (6.1-7.4 mg) as an internal standard and the sample analyzed by ^{1}H NMR spectroscopy in $CDCl_3$. The product of reductive cleavage and/or the recovered starting material were quantified by comparing the integration ratios of selected signals of the product and the starting material to that of the internal standard. Reported NMR yields are an average of two independent trials. An aliquot of the sample solution was also analyzed by GC-MS.

4.4 Reductive Cleavage of 1-Naphthyl Dimethylsulfamate with Bis(NHC)Ni^{I}Br (5)

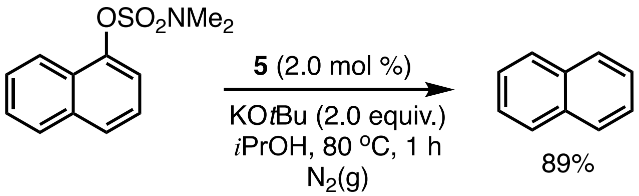

An oven-dried 4" reaction tube equipped with a PTFE-coated stir was charged with 1-naphthyl dimethylsulfamate (62.4 mg, 0.248 mmol, 1.0 equiv.) and KO*t*Bu (55.5 mg, 0.495 mmol, 2.0 equiv.). The tube was transferred to a nitrogen-filled glovebox, where bis(NHC)Ni^{I}Br (**5**) (3.0 mg, 4.94 x 10^{-3} mmol, 2.0 mol %) was added, and the flask septum sealed then secured with electrical tape before removing from the glovebox. A nitrogen gas balloon was inserted via the septum and isopropanol (2.5 mL) that was sparged for 30 min with nitrogen gas was transferred to the reaction tube via a syringe. The pale orange mixture was stirred at 80 °C for 1 h in an oil bath. The reaction tube was cooled and the crude mixture filtered via a Hirsch funnel through a layer of Celite and silica gel, and the tube and residue rinsed with DCM. The filtrate was transferred to a 50 mL round bottom flask and the solvent removed on a rotary evaporator and 1,3,5-trimethoxybenzene (5.5 mg) added as an internal standard followed by $CDCl_3$. The sample was analyzed by ^{1}H NMR spectroscopy. Quantification of the product was achieved by comparing the integration ratios of a selected product signal to that of the internal standard. Reported NMR yields are an average of three independent trials. The average NMR yield of the three independent trials is 89 ± 2.5 %. An aliquot of the sample solution was further analyzed by GC-MS and no additional products were detected.

4.41 Catalyst activation pathway for 3a–3h under reductive cleavage conditions

The pathway involves β-hydride elimination from an in situ generated Ni(II) diisopropoxide species with reductive-elimination of *i*PrOH, thereby reducing Ni(II) to Ni(0).[22-23]

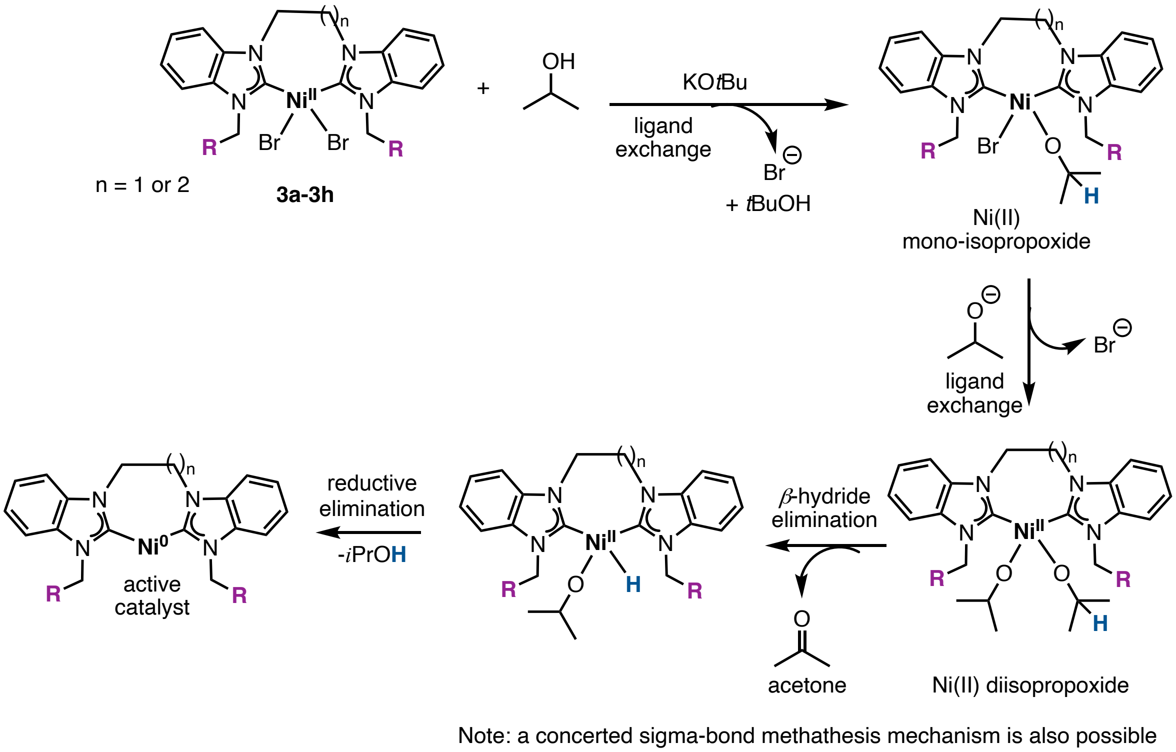

Scheme S2. Tentatively proposed activation pathway for **3a–3h** under reductive cleavage conditions

4.5 Suzuki-Miyaura Coupling Reaction with Bis(NHC)Ni^{I}Br (5)

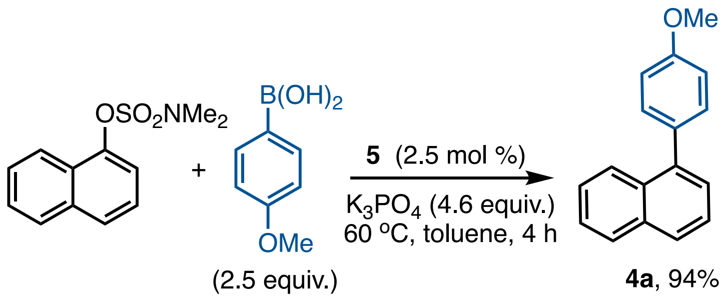

Compound **4a** was prepared according to the general procedure with the representative quantities: K_3PO_4 (248.7 mg, 1.17 mmol, 4.5 equiv.), 1-naphthyl dimethylsulfamate (64.1 mg, 0.255 mmol, 1.0 equiv.), **5** (3.9 mg, 0.0064 mmol, 2.5 mol %), 4-methoxyphenylboronic acid (103.7 mg, 0.648 mmol, 2.5 equiv.), dry toluene (2.5 mL) and the reaction mixtures stirred at 60 °C for 4 h. The average NMR yield of **4a** was 94% and the recovered 1-naphthyl dimethylsulfamate was 5% from two independent trials.

5.0 Determination of σ-Donating Properties of Bis(NHCs) by Estimating the $^{1}J_{C\text{-}H}$ Coupling Constant

The σ-donating properties of the bis(NHCs) were estimated from the ^{13}C satellites of the ^{1}H NMR measurements from the NC**H**N signal of the benzimidazolium bromides, **2a–2h** dissolved in DMSO-d_6 (0.60 mL). The determination is based on the empirical relationship between the $^{1}J_{CH}$ coupling and the hybridization (s-character) at the carbon atom involved.[24-26] Spectra were acquired at 25 °C over 128 scans, and processed using MNova 14.3.0.

Table S4. Ligand Parameter, $^{1}J_{CH}$ (Hz) for the bis(NHCs) of precatalysts 3a–3h

Entry	Catalyst	$^{1}J_{CH}$ (Hz)[a,*]
1	**3a**	219.85
2	**3b**	218.45
3	**3c**	220.25
4	**3d**	219.85
5	**3e**	221.20
6	**3f**	220.30
7	**3g**	221.82
8	**3h**	221.35

[a]NMR spectra for the benzimidazolium salts recorded in DMSO-d_6. *Determined from ^{13}C satellites in the ^{1}H NMR spectrum of the benzimidazolium bromides.[26]

6.0 Percent Buried Volume Calculations.

The $\%V_{Bur}$ values are calculated at a radius of 3.5 Å radius centered on nickel[27] using the SambVca 2.1 web app.[28] Crystallographic coordinates (.xyz) for each complex were used in the calculations and H-atoms were not included in the model. The topographic steric maps of the bis(NHC) ligands are shown below with the corresponding color bars.[29] For the bis(NHCs) of **3e–3h**, coordinates are such that the z-axis bisects the Br-Ni-Br angle and passes through the Ni center, steric maps are viewed in the xy plane. In the case of bis(NHC)Ni^{I}Br (**5**), the z-axis is parallel to the Ni-Br bond and passes through the carbene carbon.

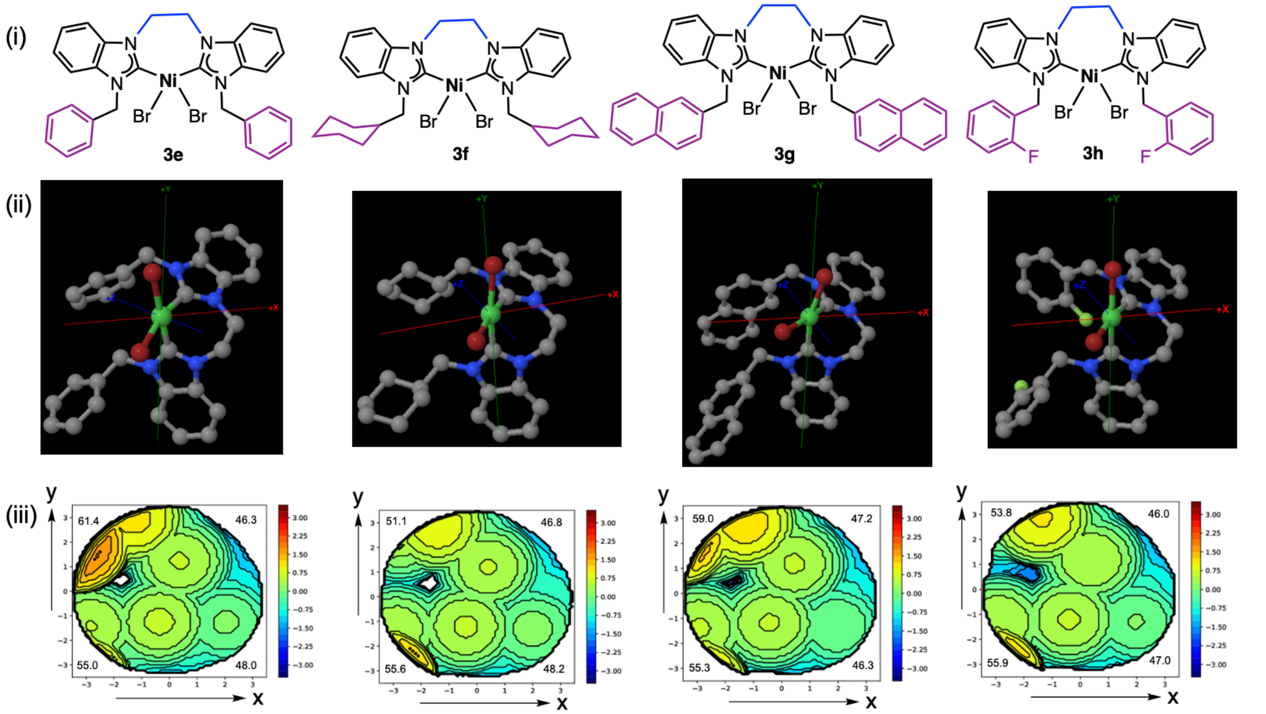

Figure S2. (i) Precatalysts **3e−3h** (ii) Preview of the 3D molecular representations of **3e−3h** as viewed down the z-axis using SambVca web app (iii) topographic steric maps for **3e−3h**.

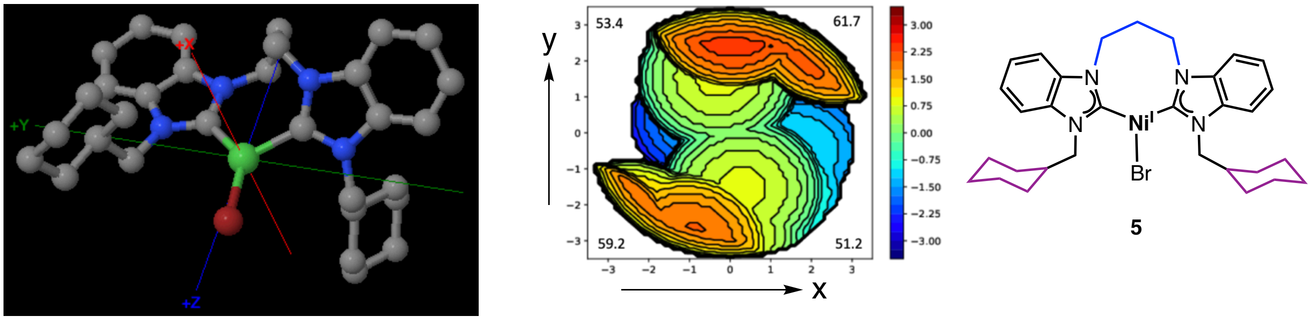

Figure S3. (i) Preview of the 3D molecular representation and topographic steric map for bis(NHC)Ni^{I}Br (**5**) as viewed down the z-axis using the SambVca web app.

Table S5. Bite Angles for the bis(NHCs) and Dihedral Angles of 3a–3h

Entry	Catalyst	Bite Angle (C_1-Ni-C_2, °)	%V_{Bur}	Dihedral Angles[b] (N-C_1-Ni-C_2, °), (C_1-Ni-C_2-N, °)	Average Dihedral Angle[b] (N-C_1-Ni-C_2, °)
1	**3a**	85.89(3)	51.8	83.58, 83.24	83.41
2	**3b**	88.78(9)	53.8	84.69, 81.50	83.10
3	**3c**	87.04(10)	52.3	76.75, 80.12	78.44
4	**3d**	87.06(8)	51.8	74.46, 83.72	79.09
5	**3e**	87.07(9)	52.6	59.49, 75.47	67.48
6	**3f**	86.08(9)	50.4	68.53, 71.17	69.85
7	**3g**	85.61(11)	52.0	68.35, 68.94	68.65
8	**3h**	84.92(14)	50.7	61.96, 71.12	66.54

%V_{Bur} values are calculated at the crystallographically-determined Ni-C distance, with sphere radius 3.5 Å, bond radii 1.17 Å, mesh spacing 0.1 Å with H atoms and solvent molecules excluded. %V_{Bur} and bite angles for **3a–3h** were reported in 2024.[4] [b]The dihedral angle is defined as the twist angle between the NiC_2Br_2 coordination plane and the plane through each NHC ring.

7.0 UV-Vis spectra of Ni(II) Complexes (3e-3h)

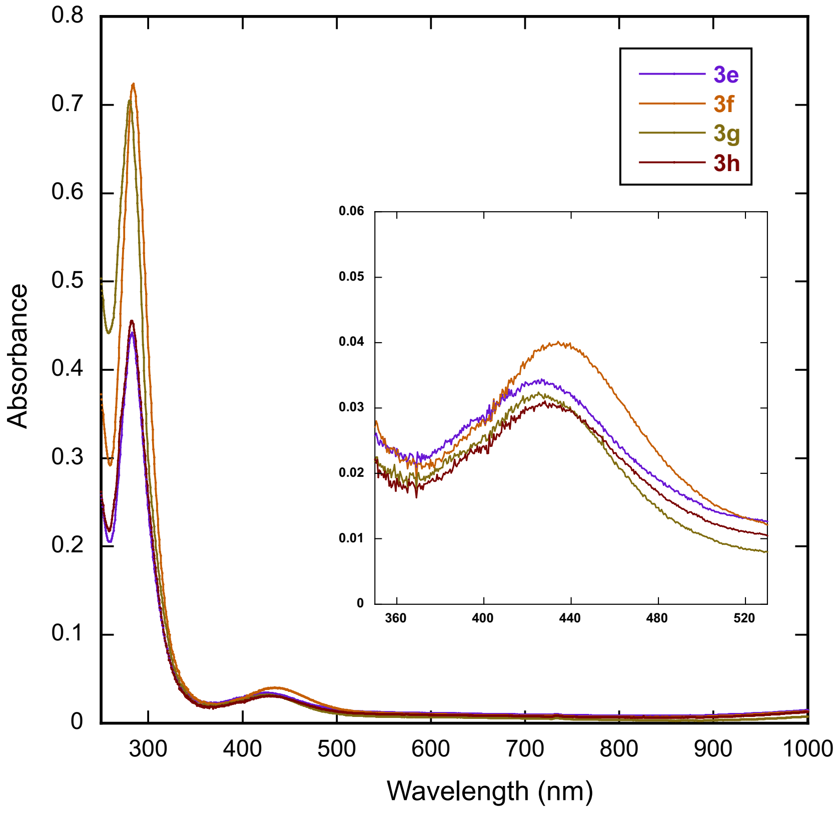

Figure S4. UV-Vis (DCM, 25 °C) spectra for **3e** (0.0163 mM), **3f** (0.0161 mM), **3g** (0.0161 mM), **3h** (0.0161 mM) at room temperature. Inset shows expansion from λ = 350-530 nm. The DCM solutions of the complexes are yellow to golden-yellow in color.

8.0 IR Spectra for bisbenzimidazolium salts (2e-2h)

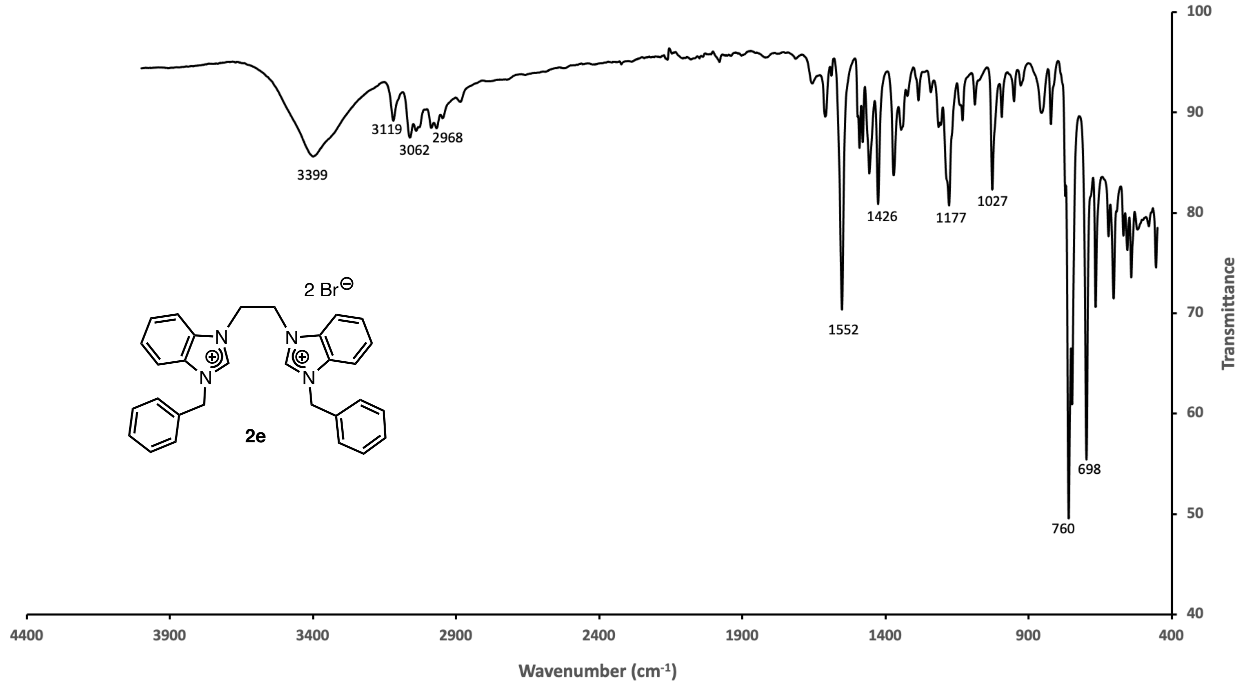

Figure S5: ATR IR spectrum of bisbenzimidazolium bromide **2e**.

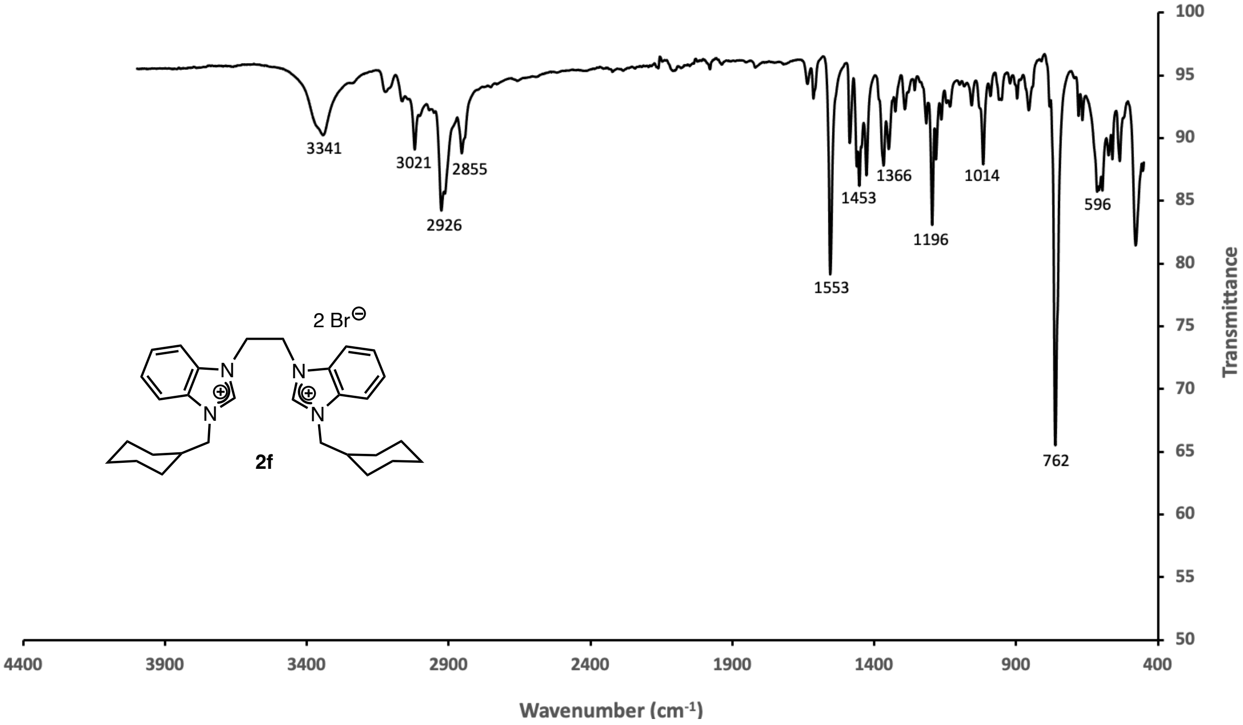

Figure S6: ATR IR spectrum of bisbenzimidazolium bromide **2f**.

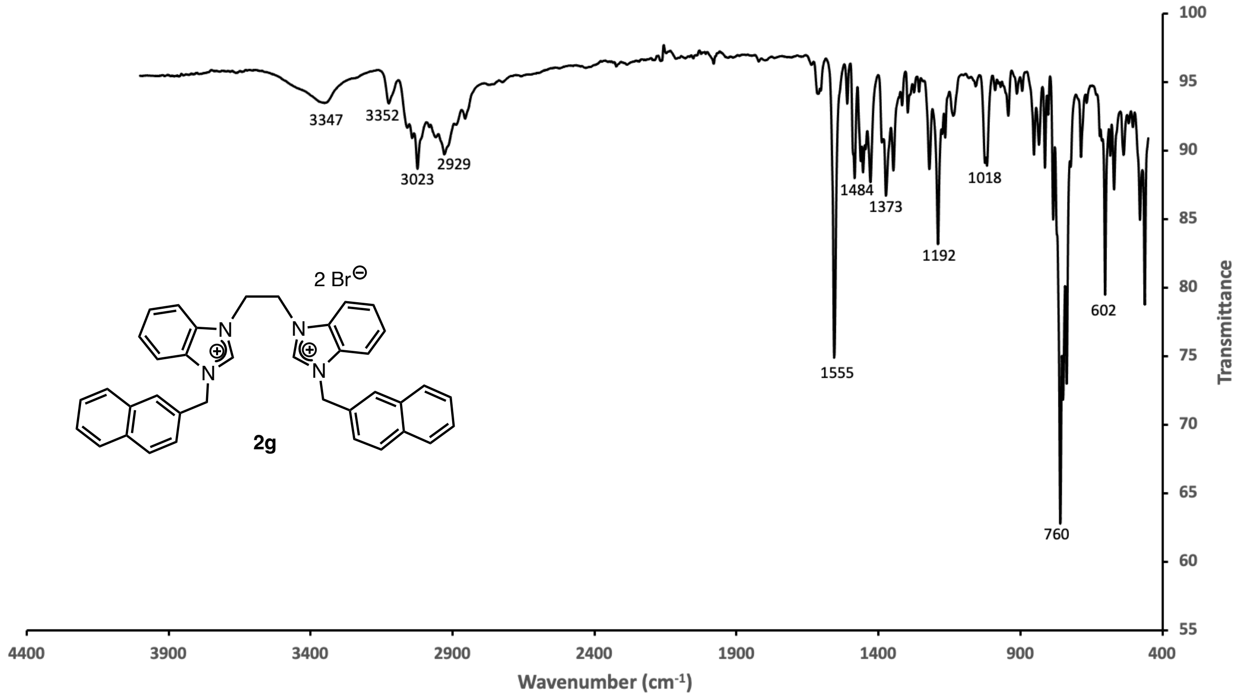

Figure S7: ATR IR spectrum of bisbenzimidazolium bromide **2g**.

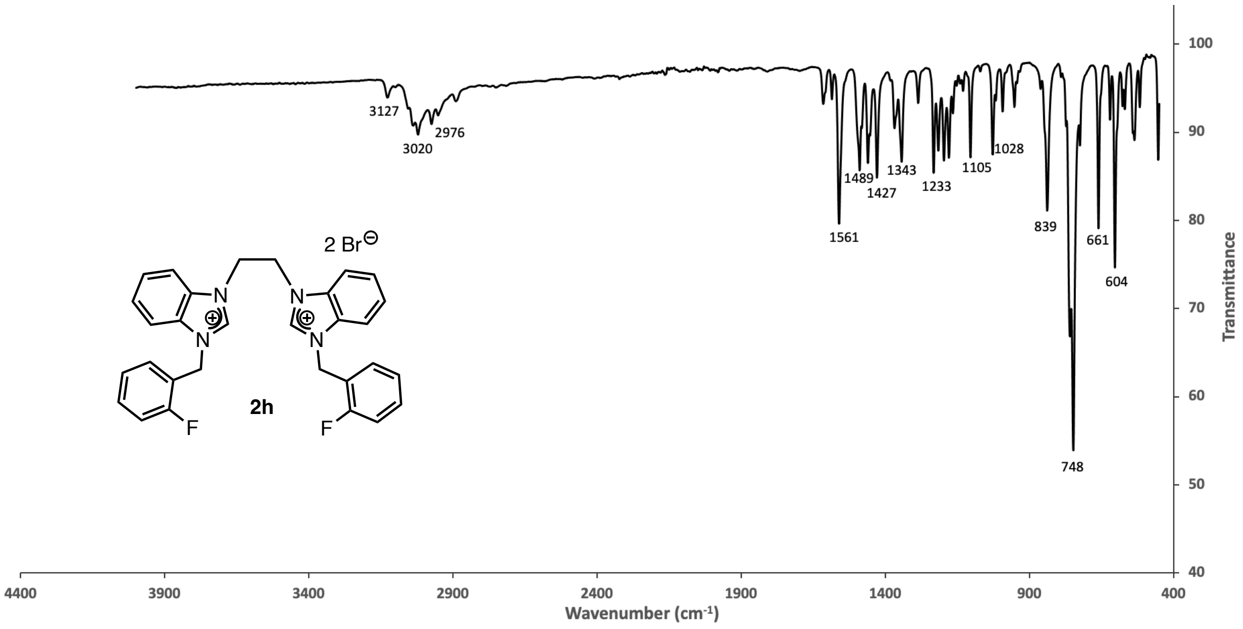

Figure S8: ATR IR spectrum of bisbenzimidazolium bromide **2h**.

9.0 NMR Spectra for reported compounds

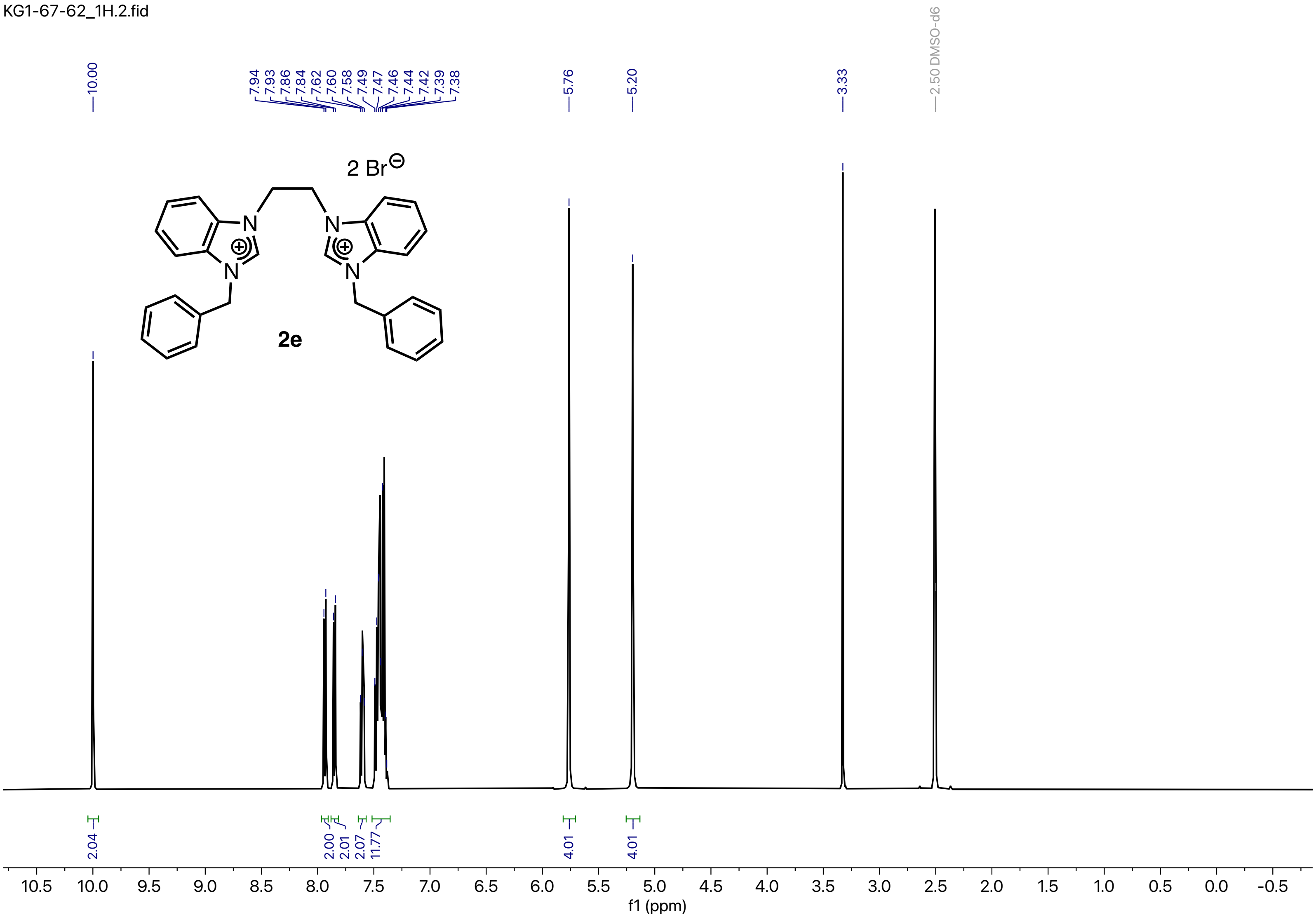

Figure S9. ^{1}**H NMR** spectrum (500 MHz, DMSO-d_6, 298 K) of **2e**.

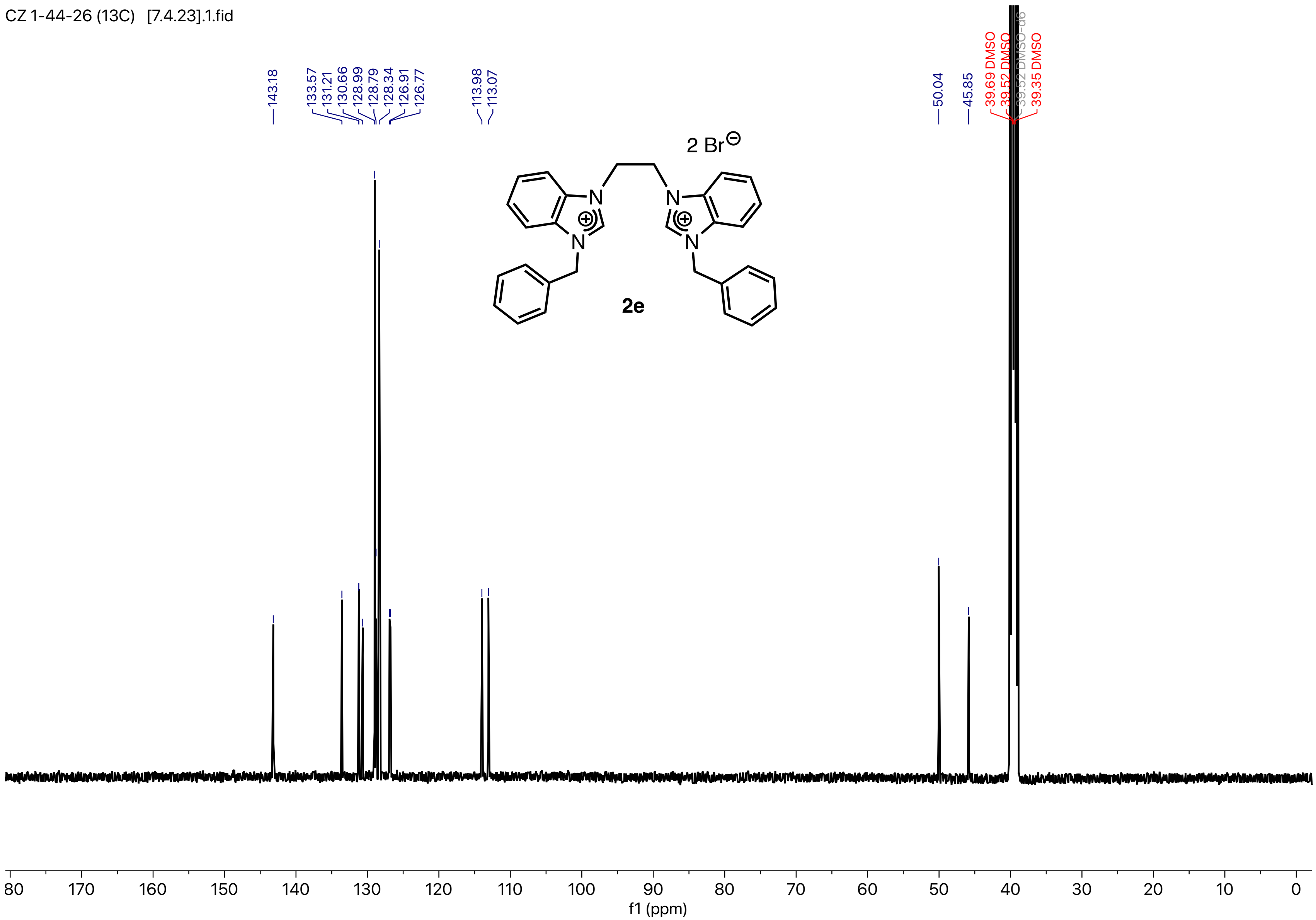

Figure S10. ^{13}**C NMR** spectrum (126 MHz, DMSO-d_6, 298 K) of **2e**.

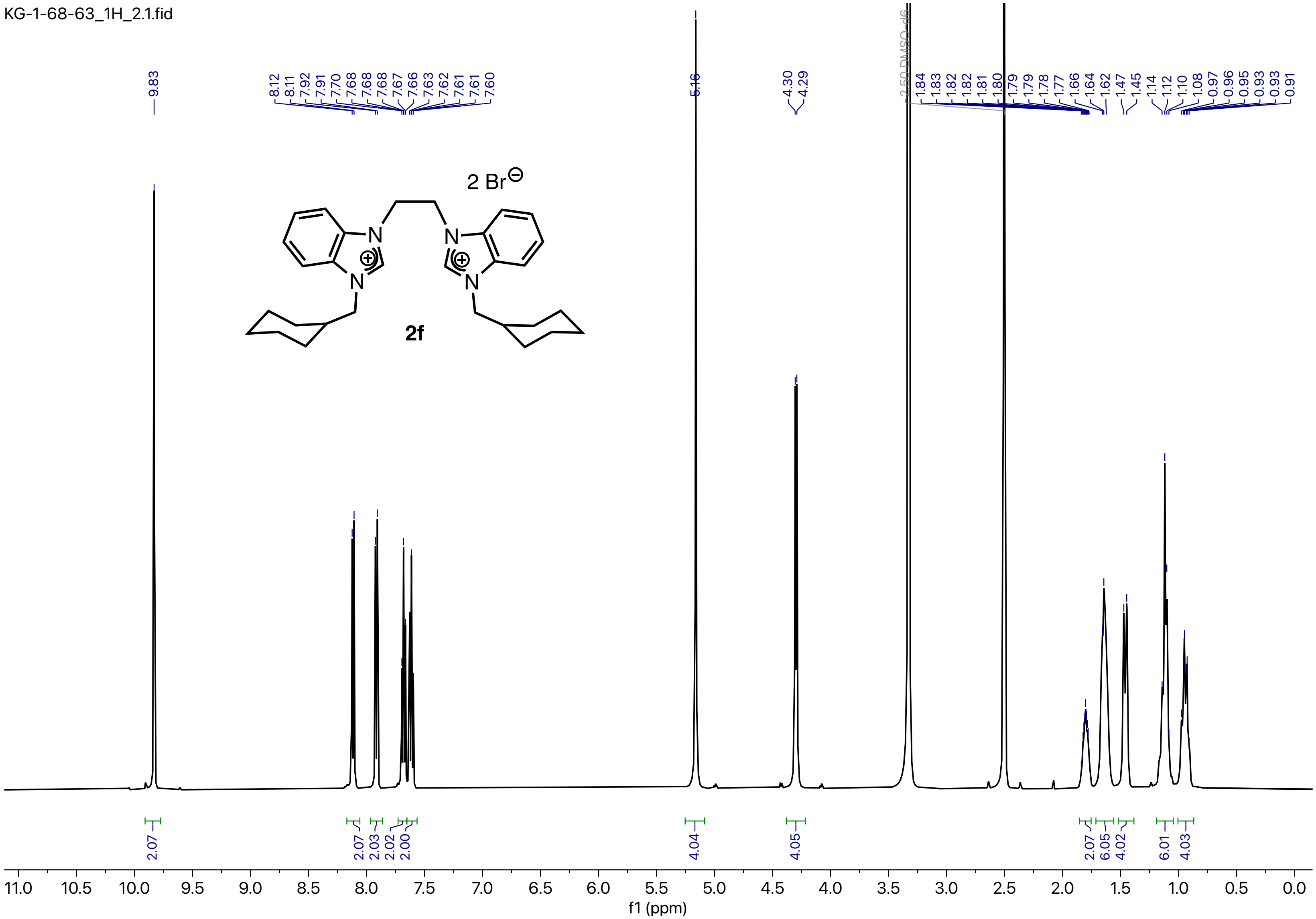

Figure S11. **^{1}H NMR** spectrum (500 MHz, DMSO-d_6, 298 K) of **2f**.

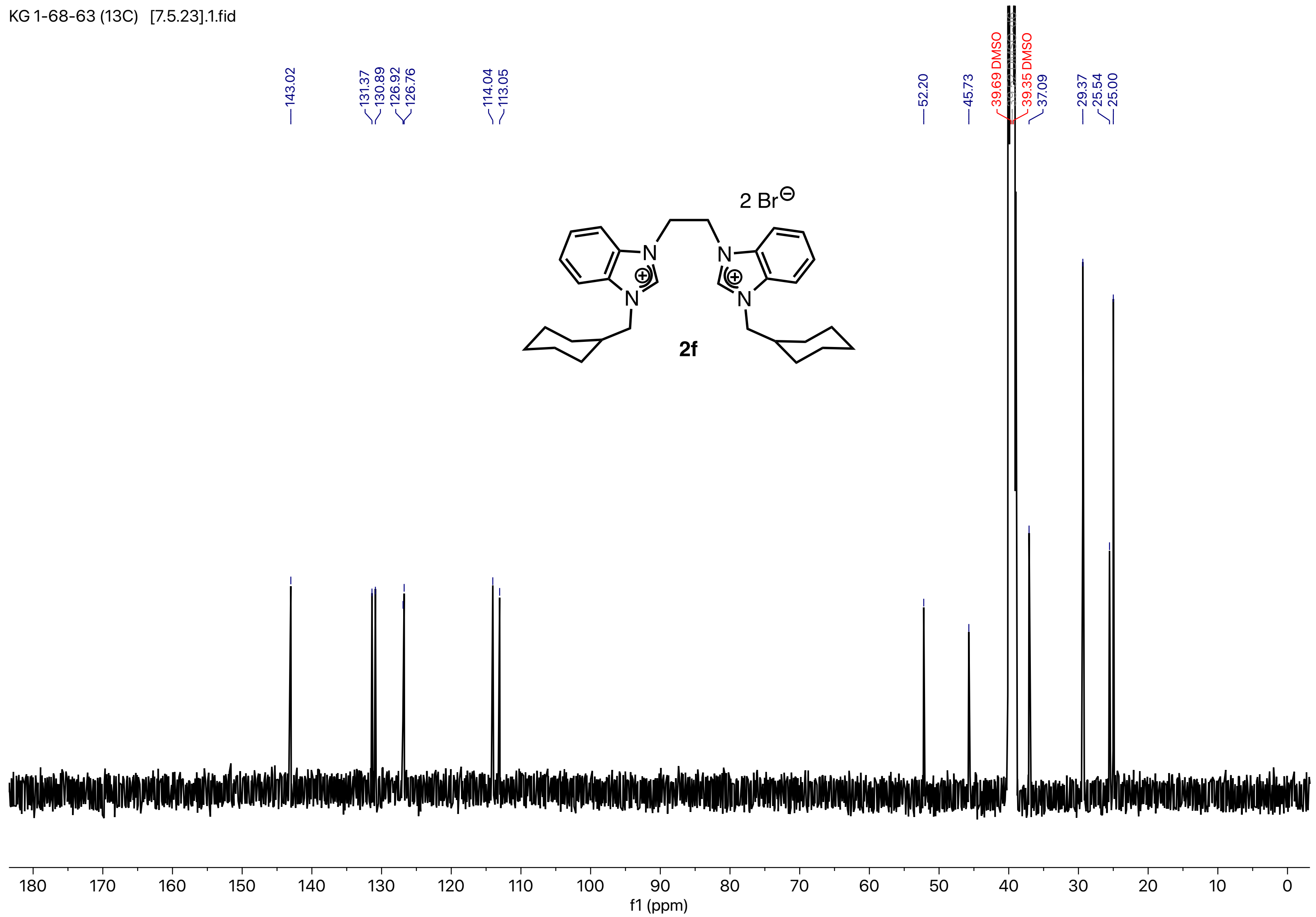

Figure S12. **^{13}C NMR** spectrum (126 MHz, DMSO-d_6, 298 K) of **2f**.

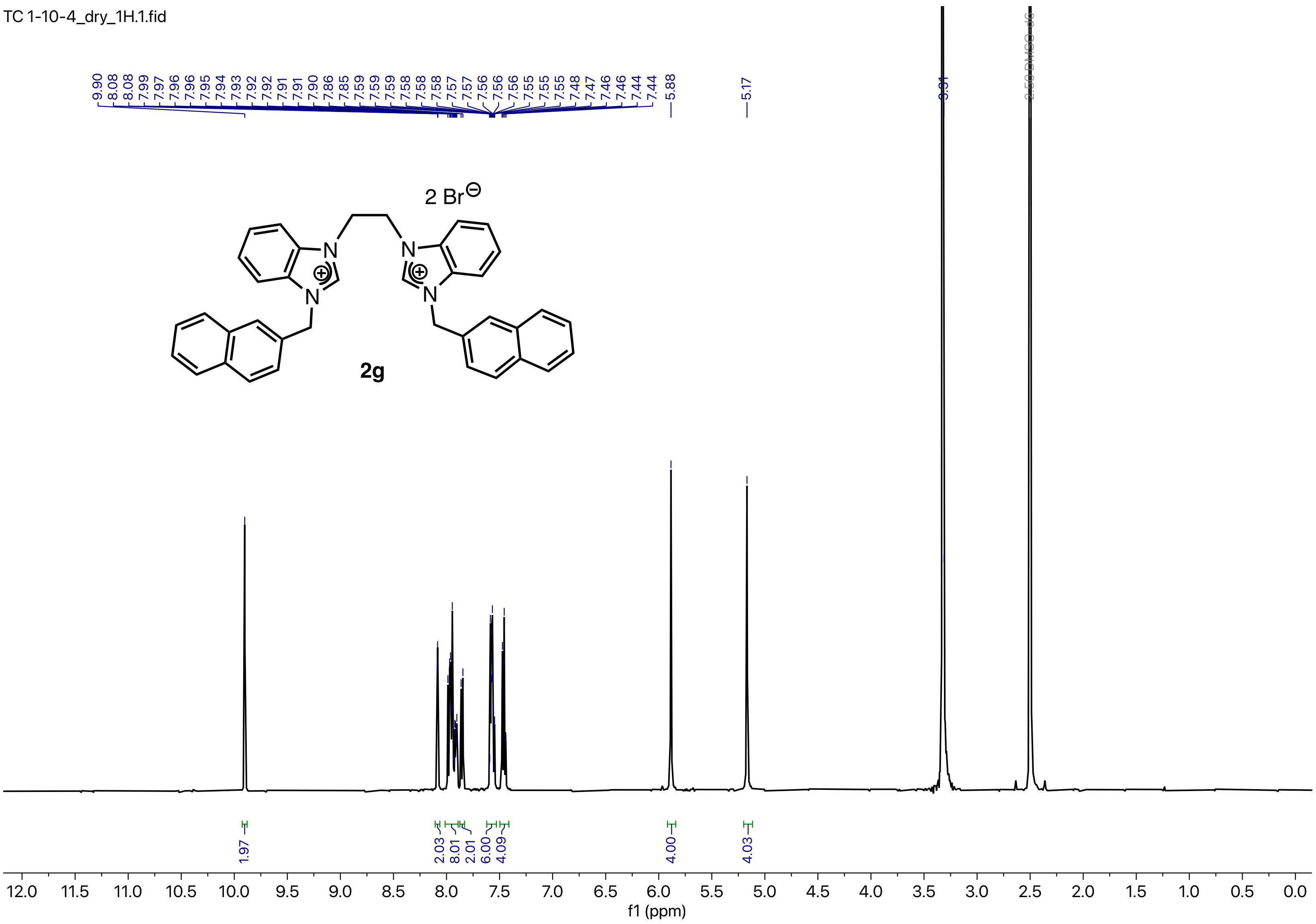

Figure S13. [1]**H NMR** spectrum (500 MHz, DMSO-d_6, 298 K) of **2g**.

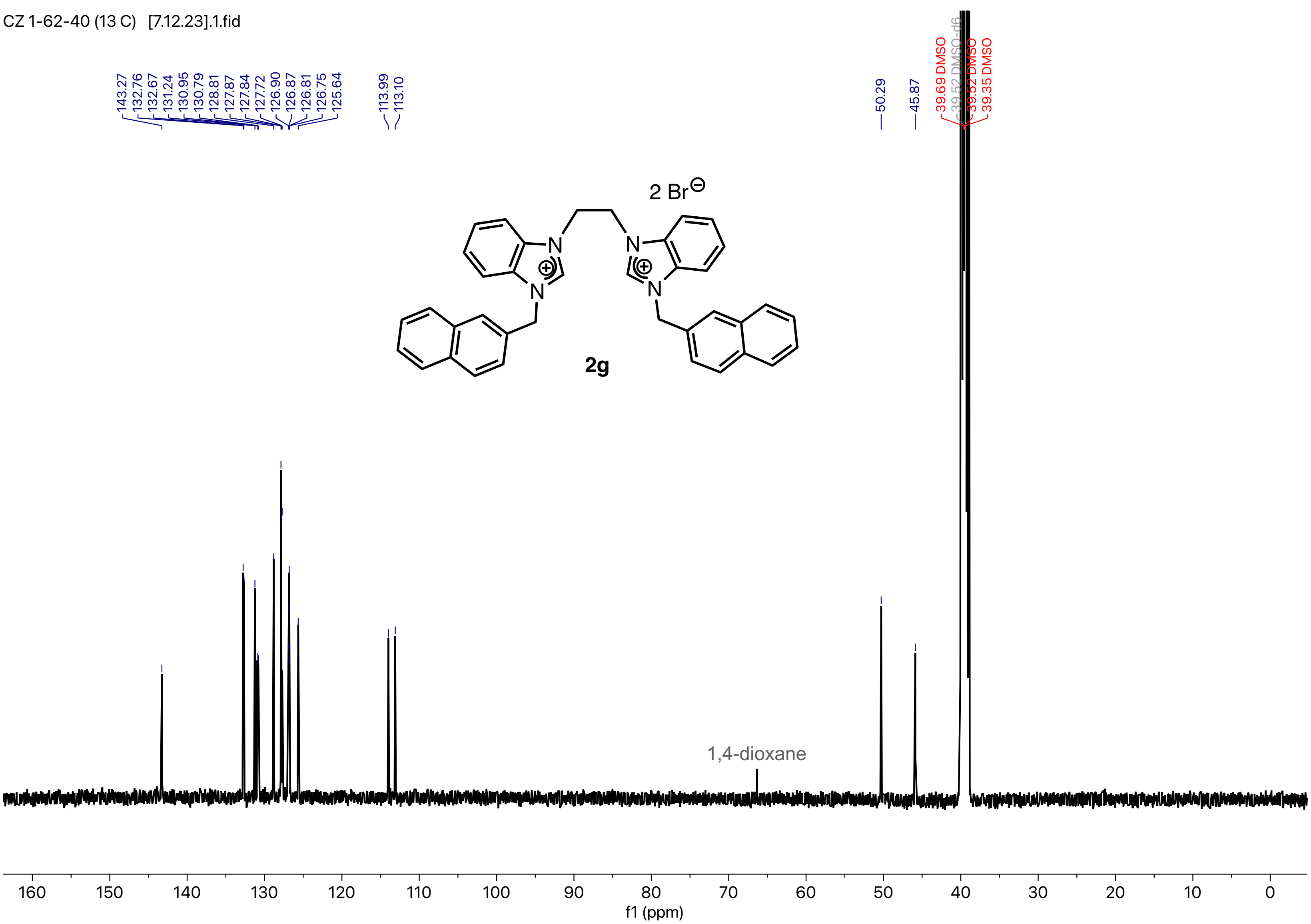

Figure S14. **^{13}C NMR** spectrum (126 MHz, DMSO-d_6, 298 K) of **2g**.

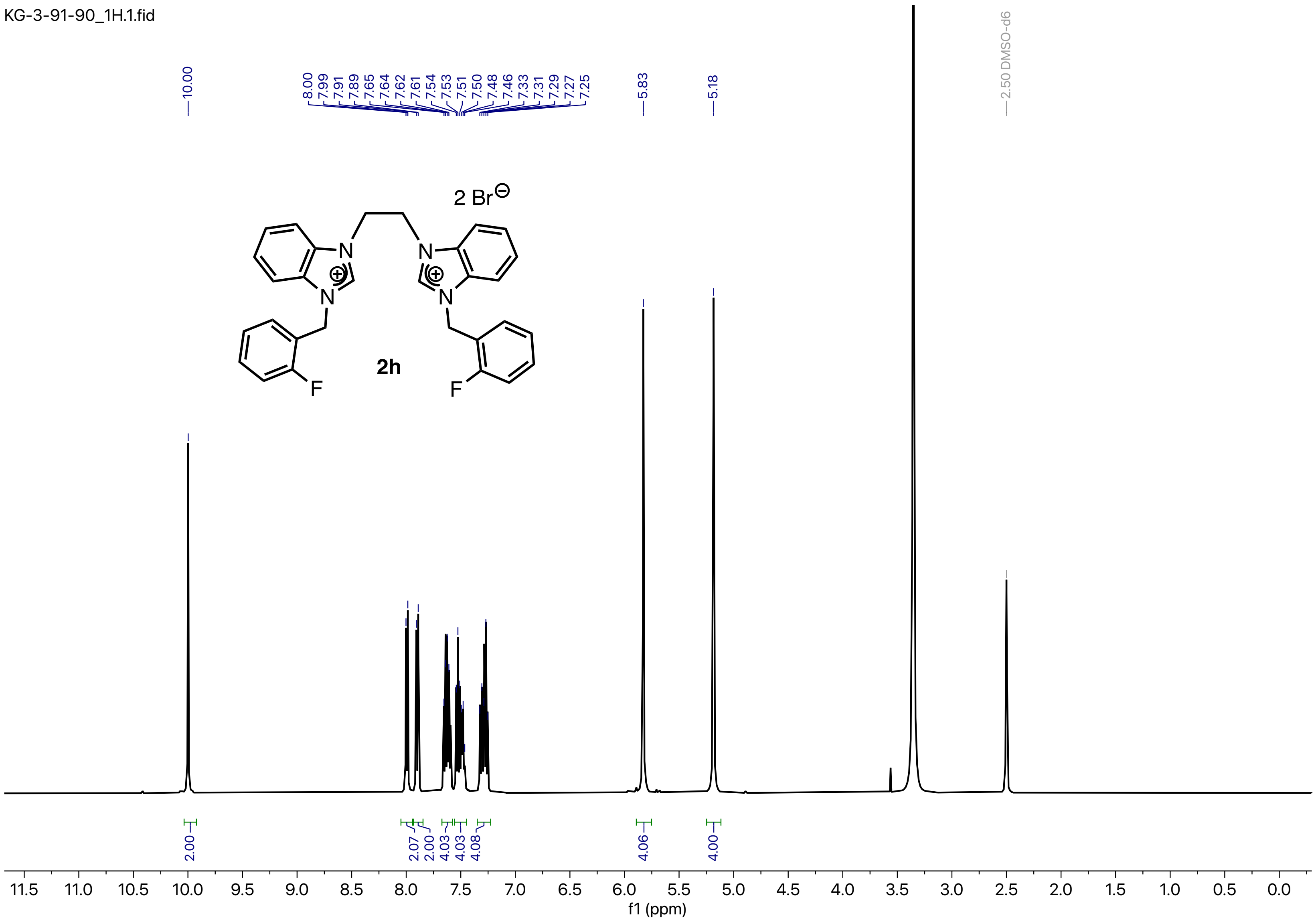

Figure S15. **^{1}H NMR** spectrum (500 MHz, DMSO-d_6, 298 K) of **2h**.

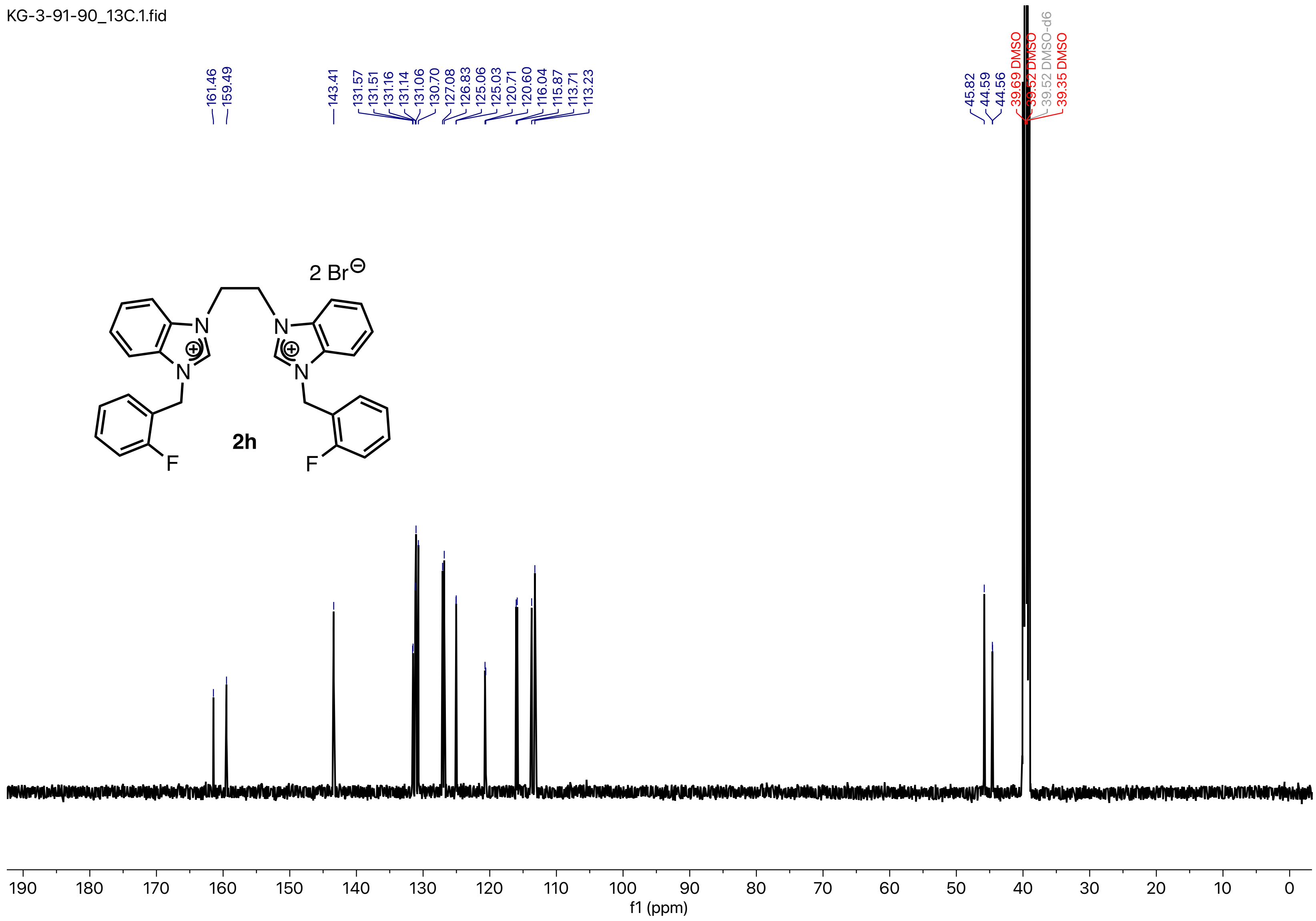

Figure S16. **^{13}C NMR** spectrum (126 MHz, DMSO-d_6, 298 K) of **2h**.

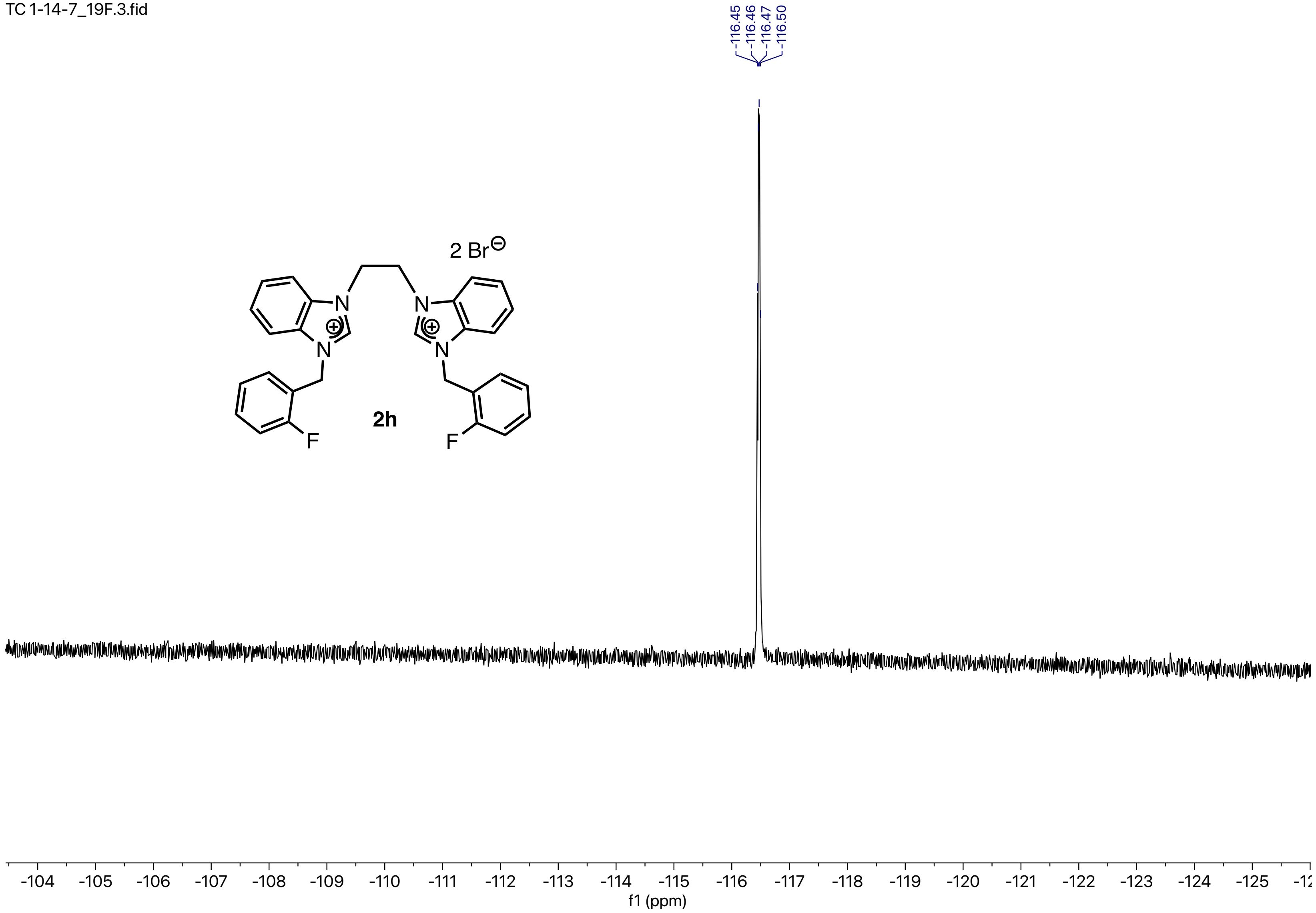

Figure S17. ^{19}**F NMR** spectrum (471 MHz, DMSO-d_6, 298 K) of **2h**.

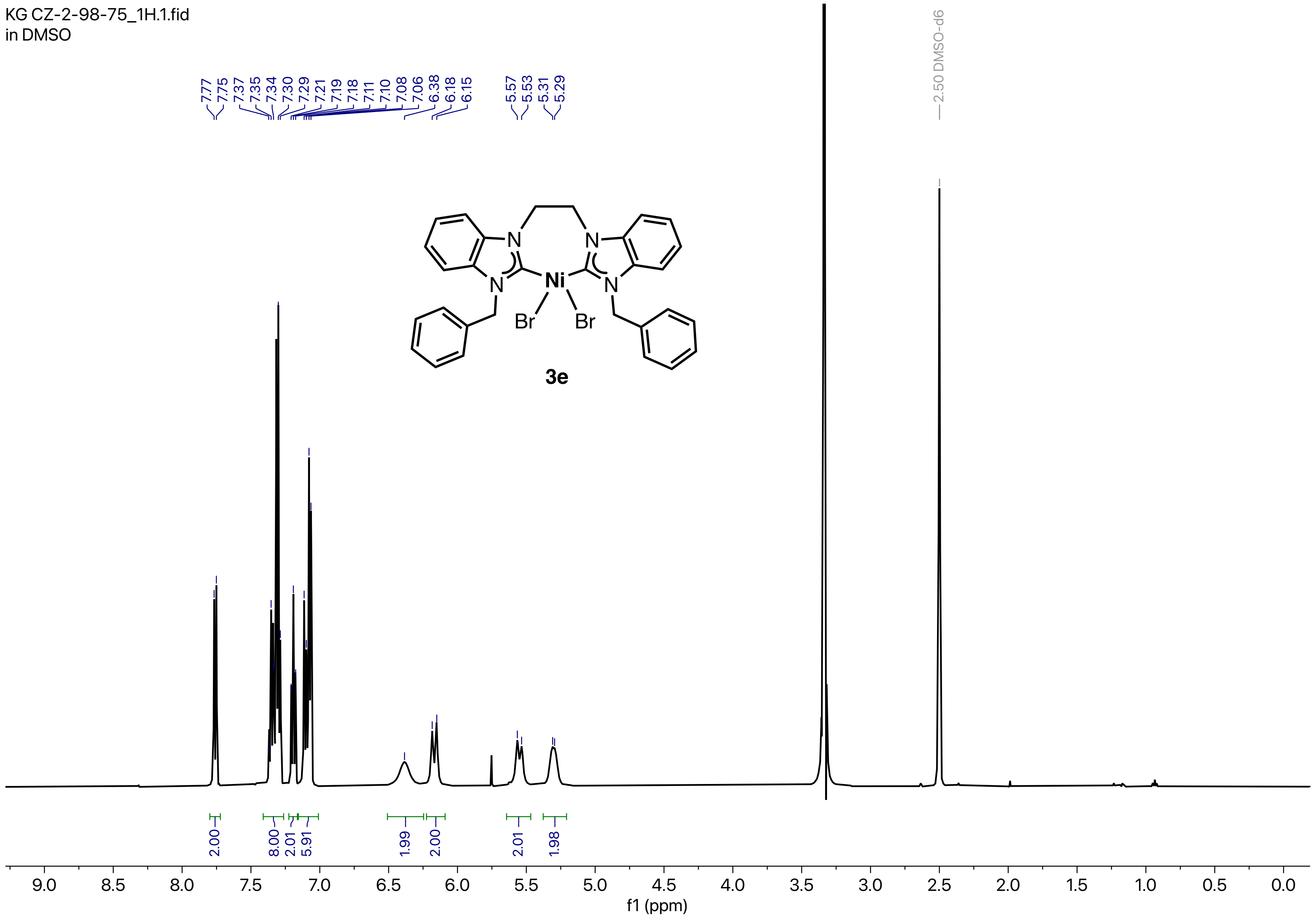

Figure S18. **^{1}H NMR** spectrum (500 MHz, DMSO-d_6, 298 K) of **3e**.

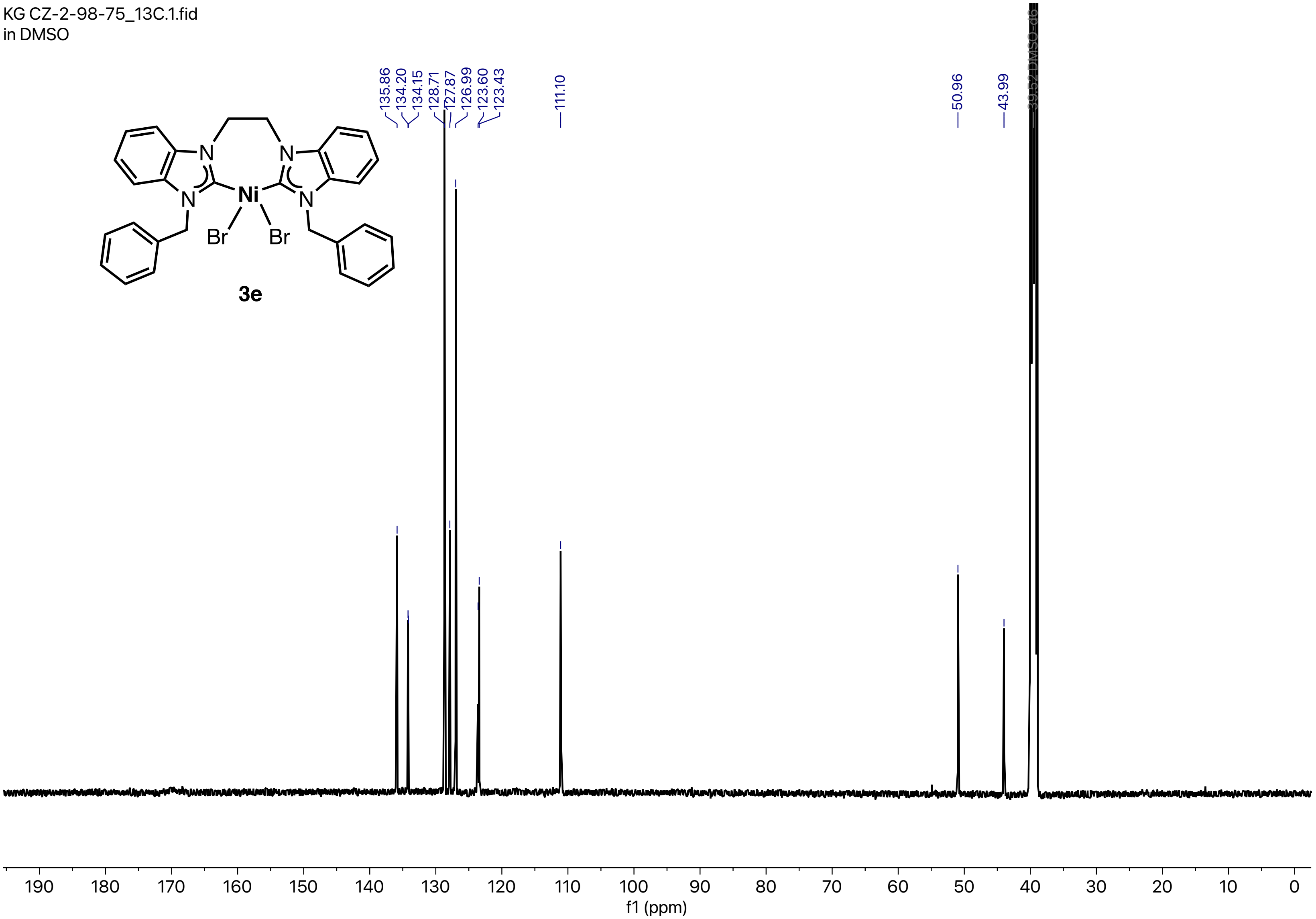

Figure S19. ^{13}**C NMR** spectrum (126 MHz, DMSO-d_6, 298 K) of **3e**.

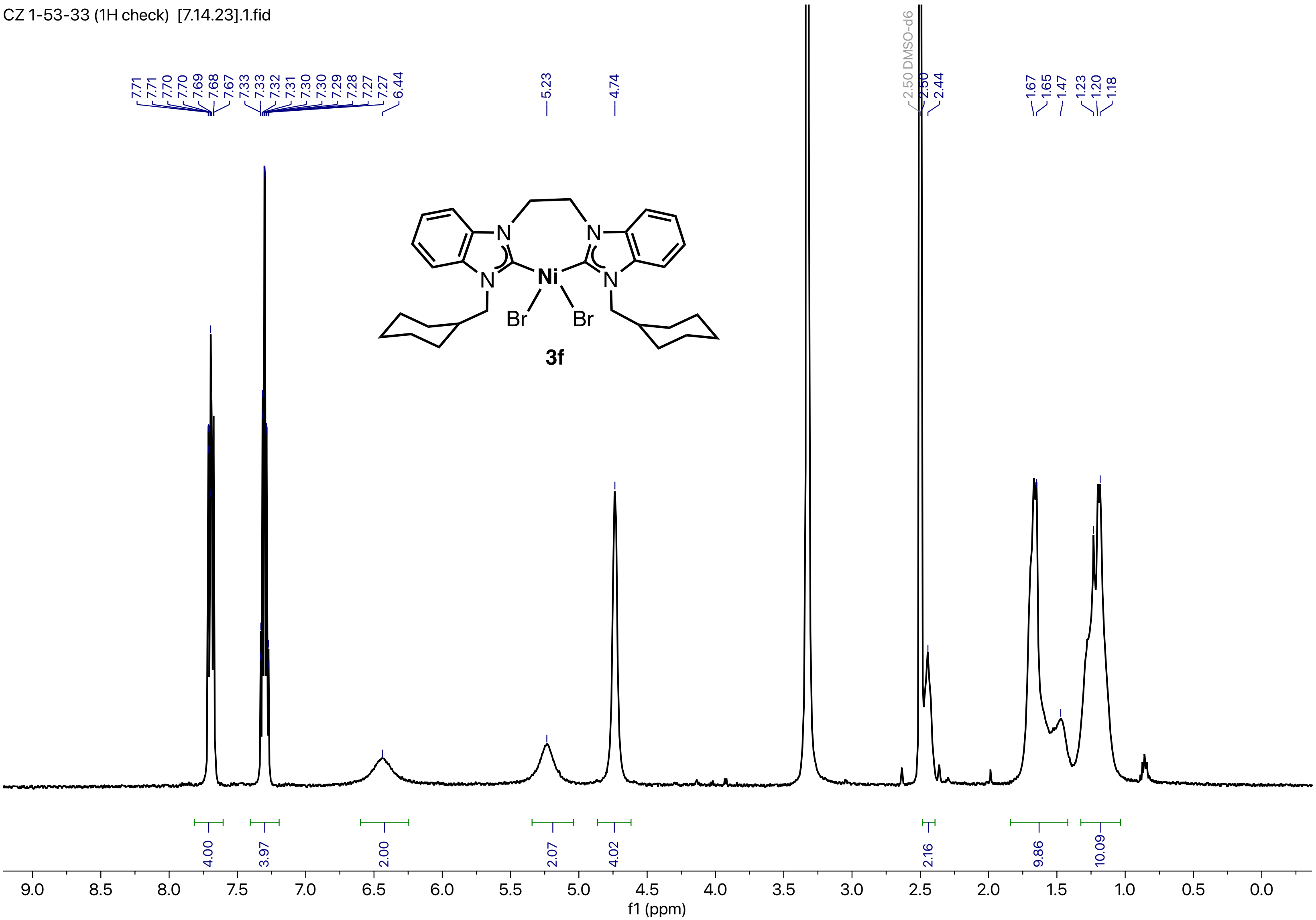

Figure S20. [1]**H NMR** spectrum (500 MHz, DMSO-d_6, 298 K) of **3f**.

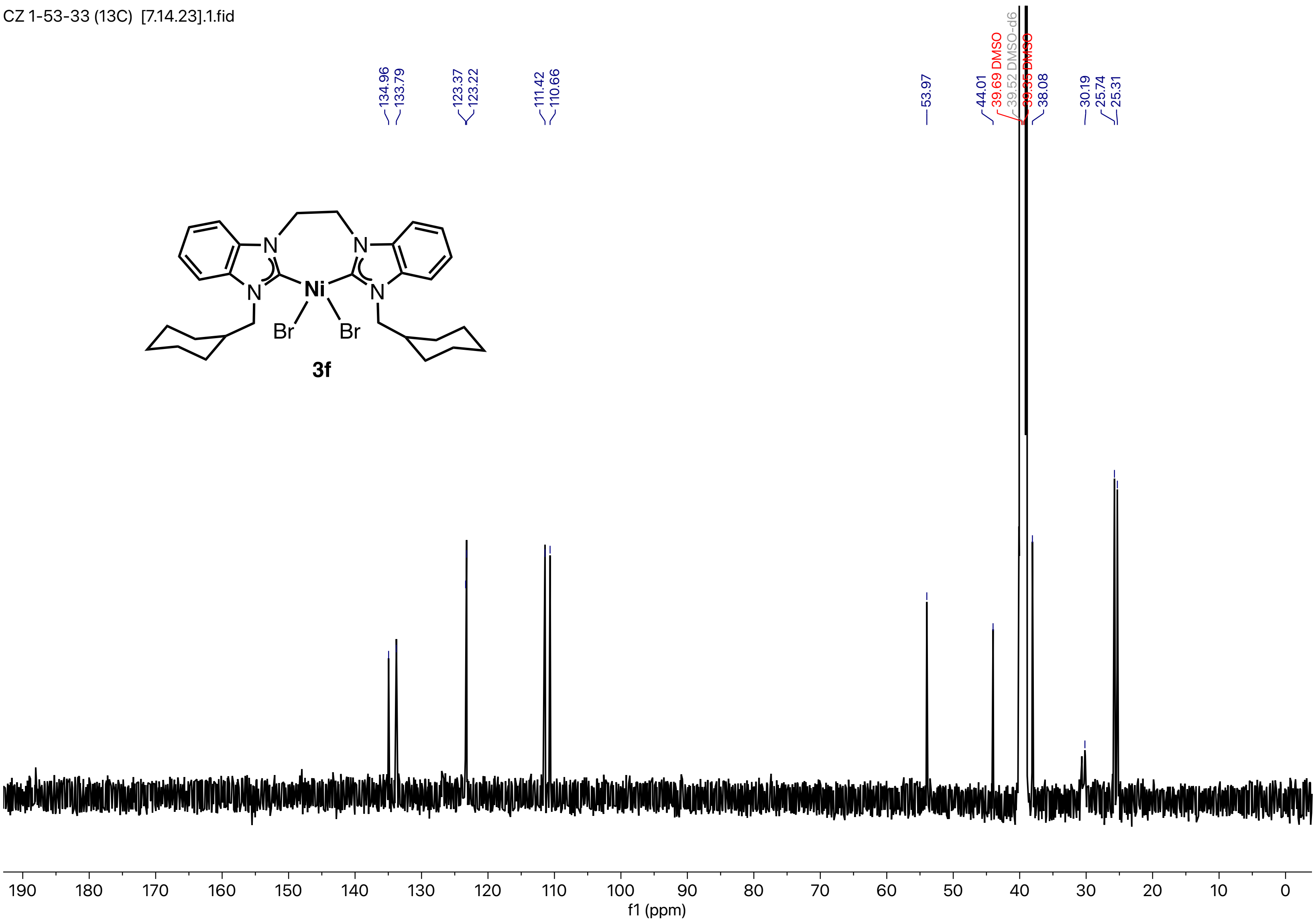

Figure S21. ^{13}**C NMR** spectrum (126 MHz, DMSO-d_6, 298 K) of **3f.**

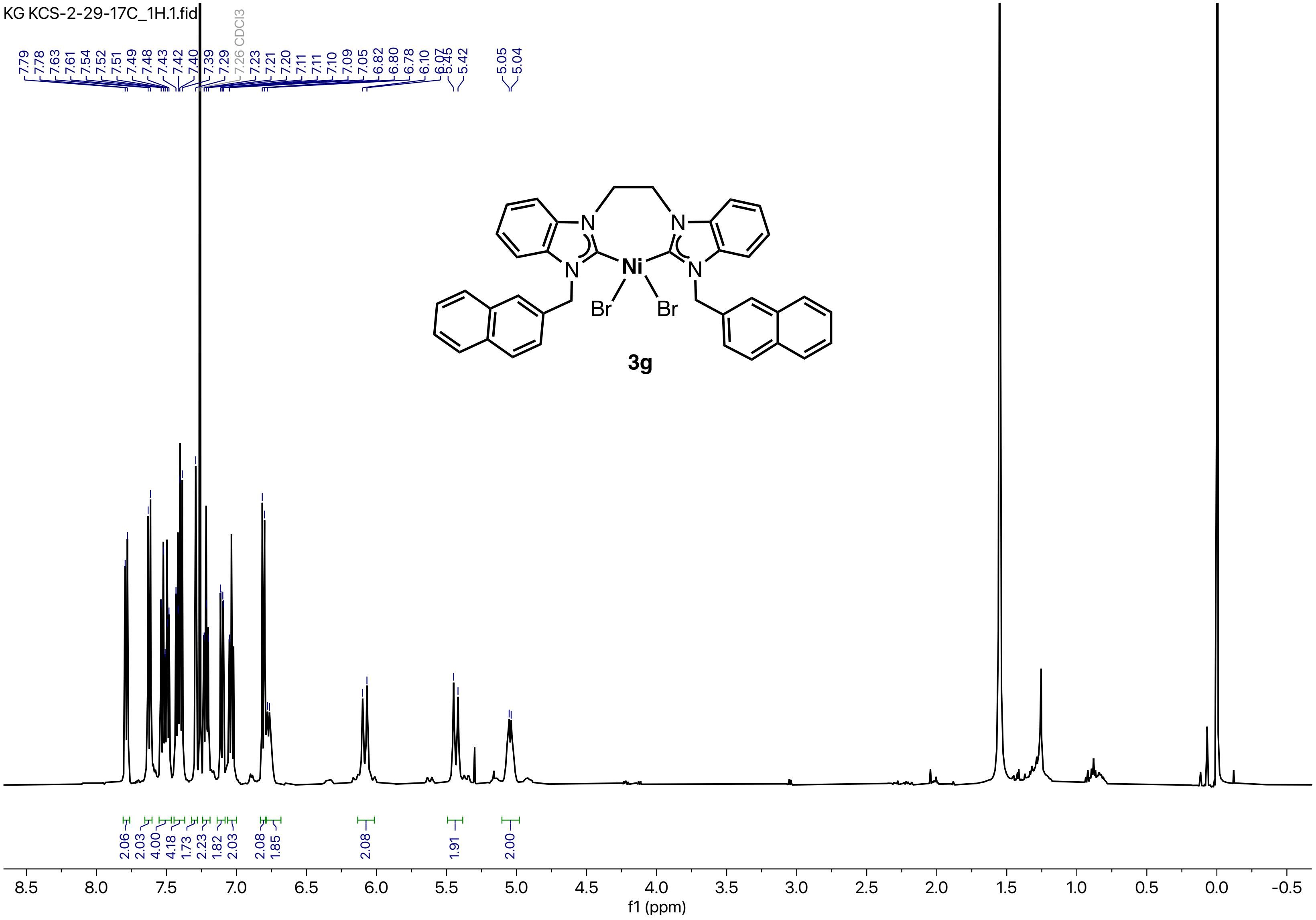

Figure S22. **¹H NMR** spectrum (500 MHz, $CDCl_3$, 298 K) of **3g**.

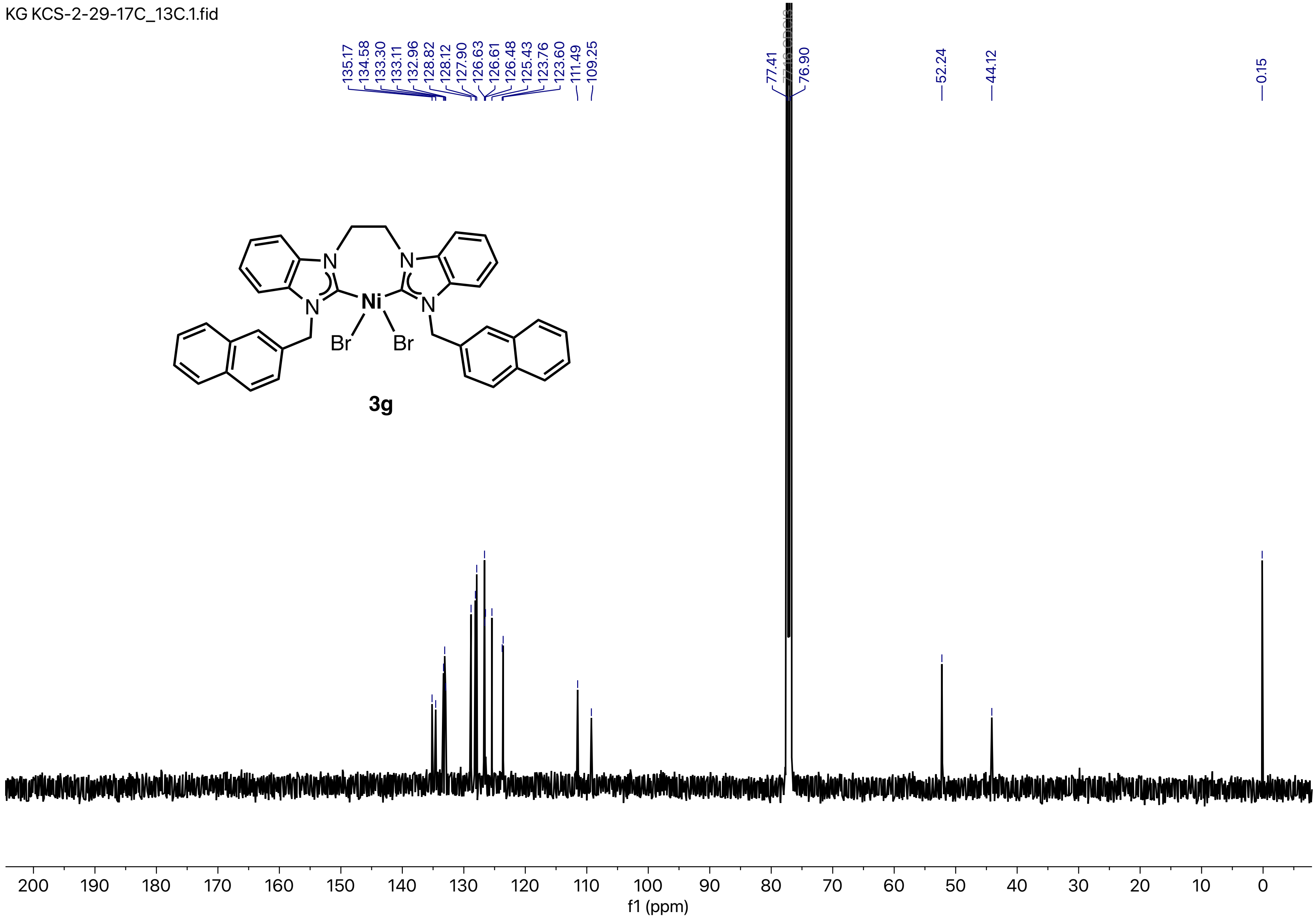

Figure S23. ^{13}**C NMR** spectrum (126 MHz, $CDCl_3$, 298 K) of **3g.**

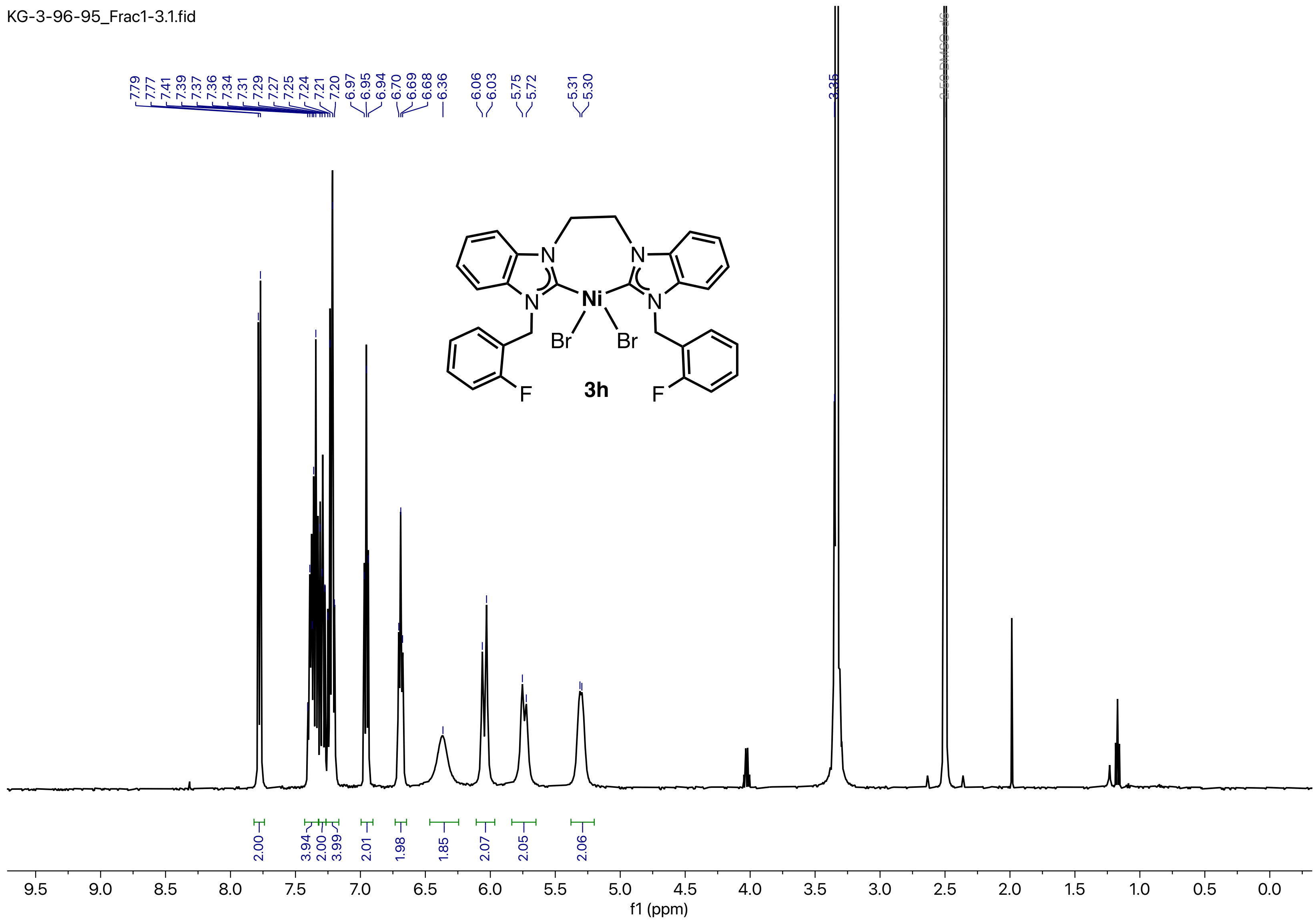

Figure S24. **^{1}H NMR** spectrum (500 MHz, DMSO-d_6, 298 K) of **3h**.

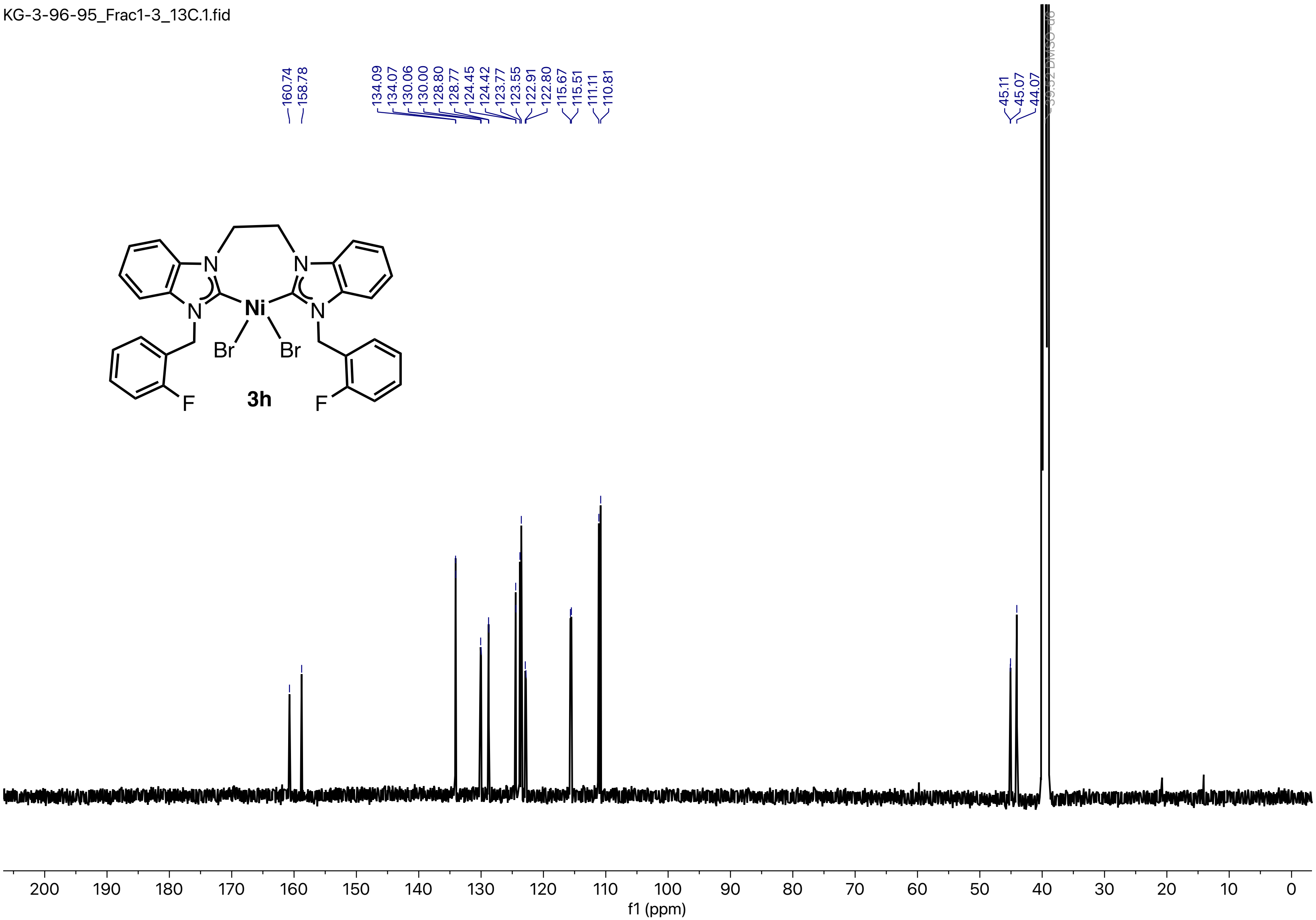

Figure S25. ^{13}**C NMR** spectrum (126 MHz, DMSO-d_6, 298 K) of **3h.**

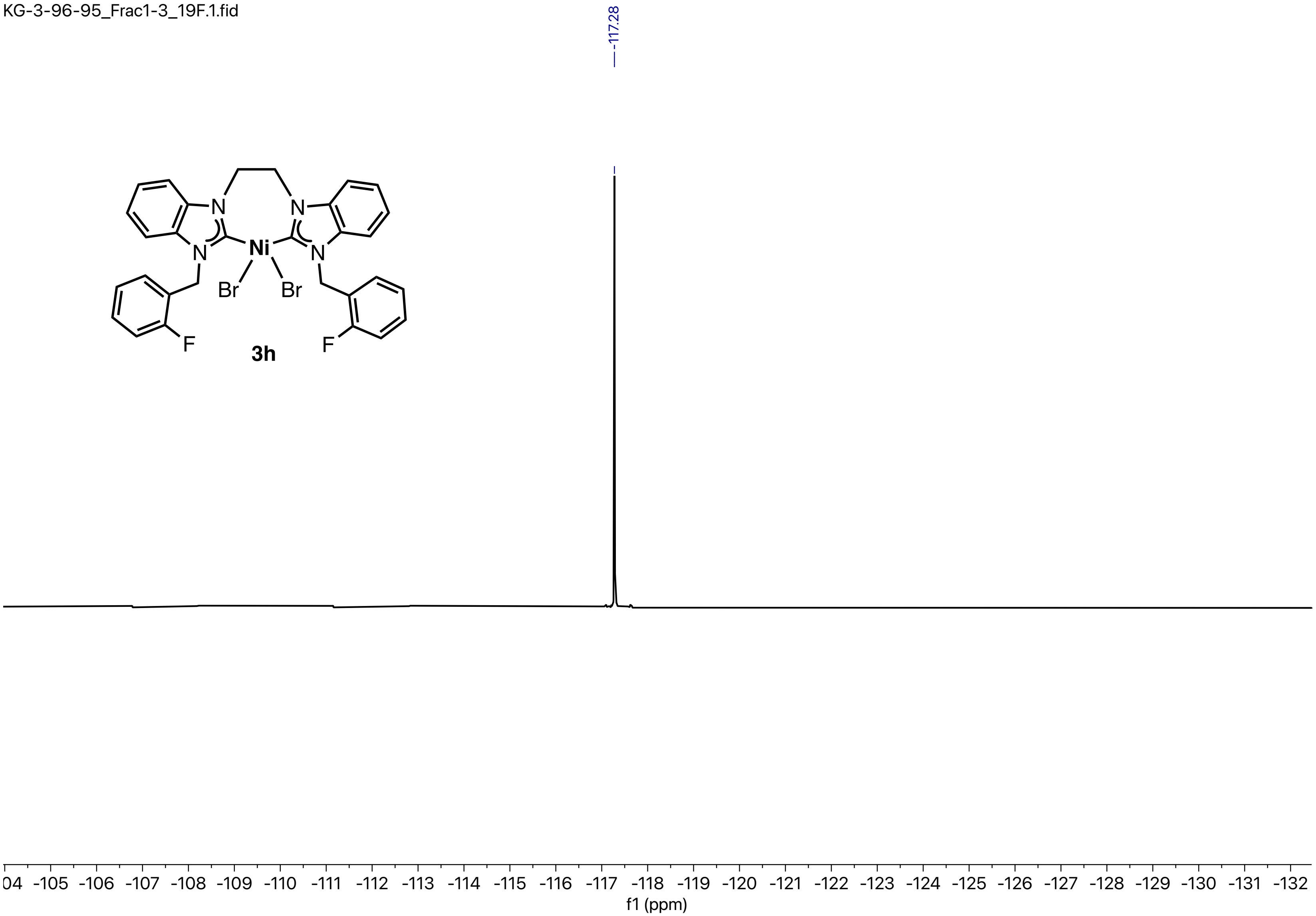

Figure S26. ^{19}F NMR spectrum (471 MHz, DMSO-d_6, 298 K) of **3h.**

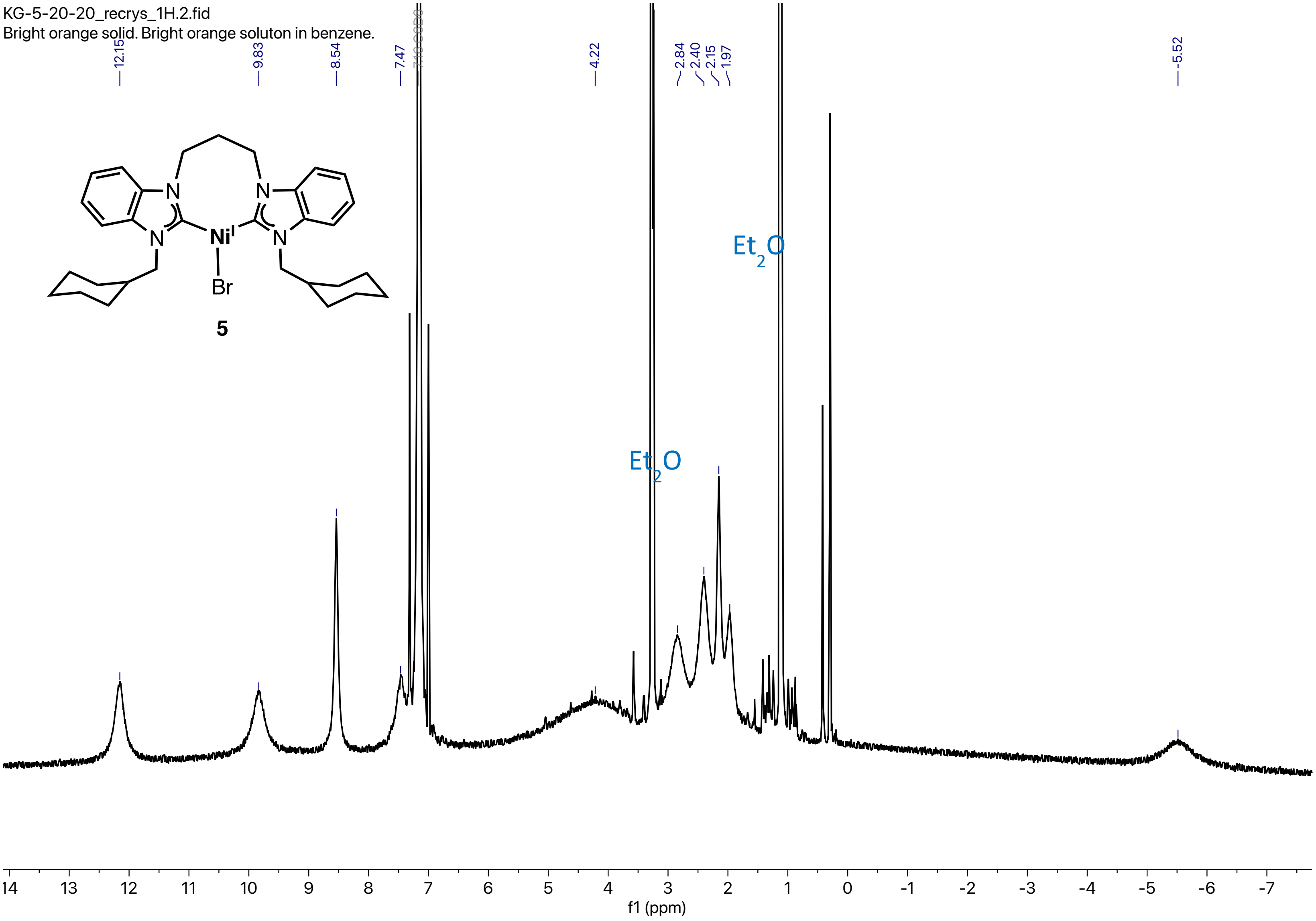

Figure S27. **^{1}H NMR** spectrum (500 MHz, C_6D_6, 298 K) of bis(NHC)Ni^{I}Br (**5**).

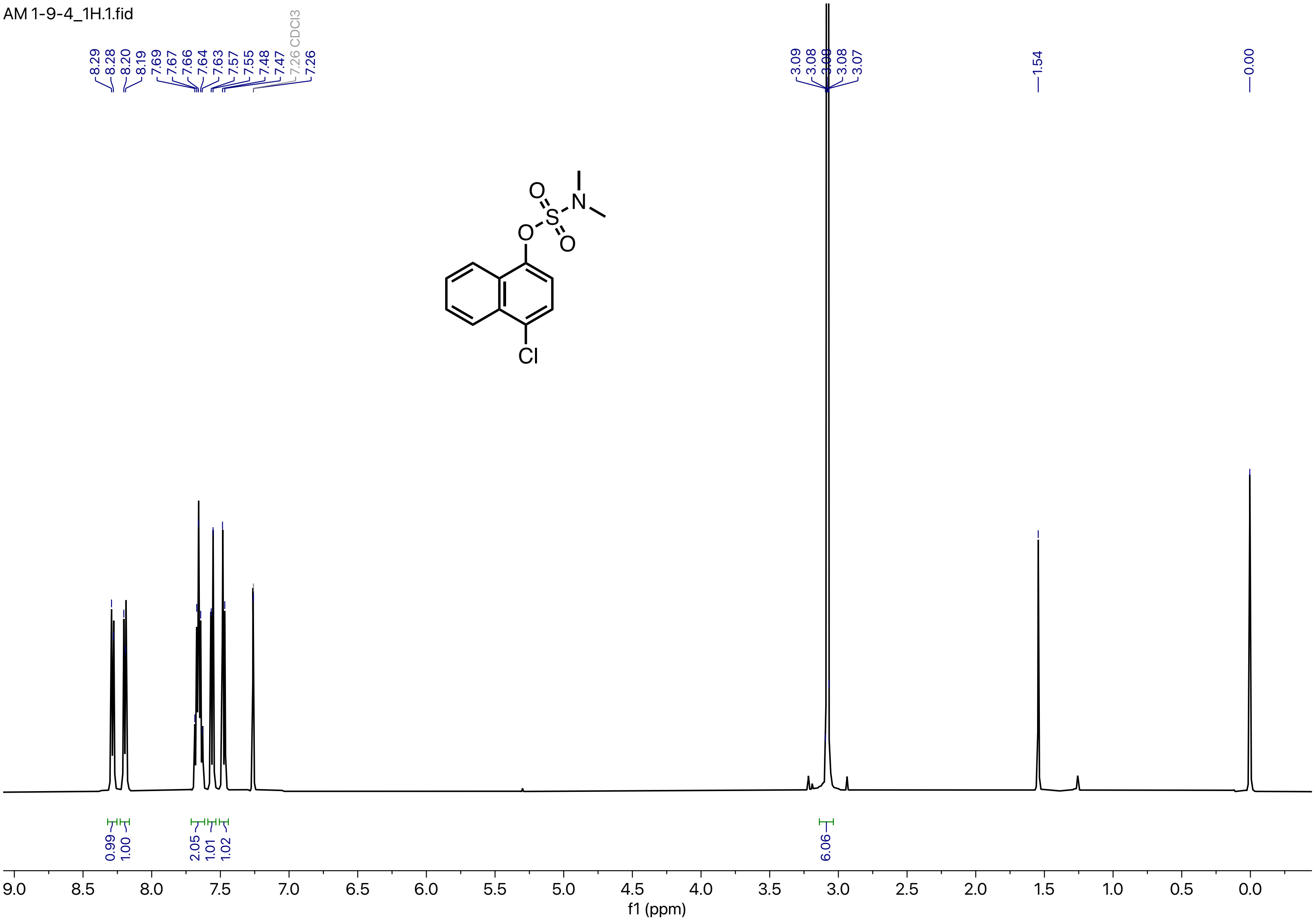

Figure S28. [1]**H NMR** spectrum (500 MHz, $CDCl_3$, 298 K) of 4-chloro-1-naphthyl dimethylsulfamate.

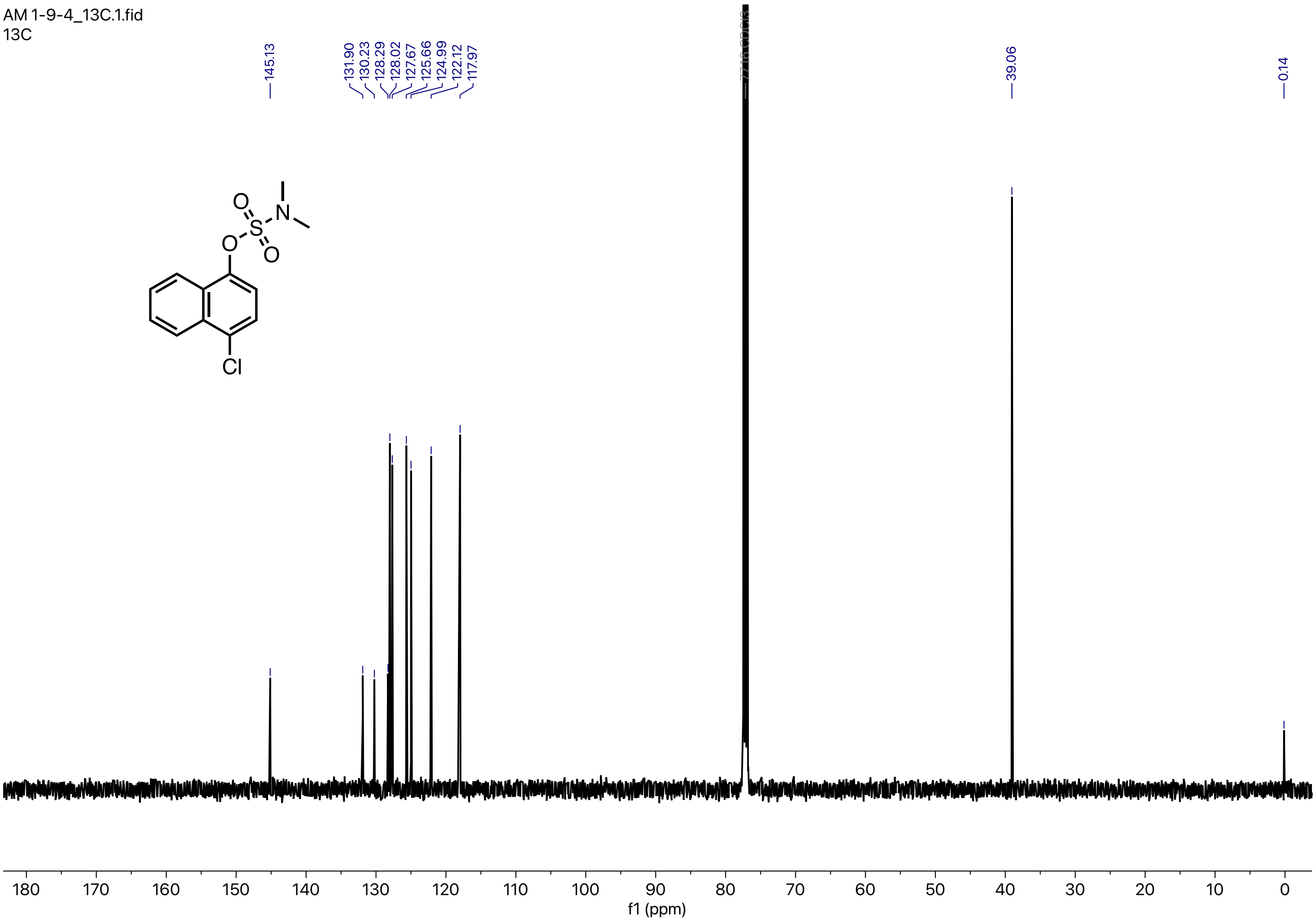

Figure S29. **^{13}C NMR** spectrum (126 MHz, $CDCl_3$, 298 K) of 4-chloro-1-naphthyl dimethylsulfamate.

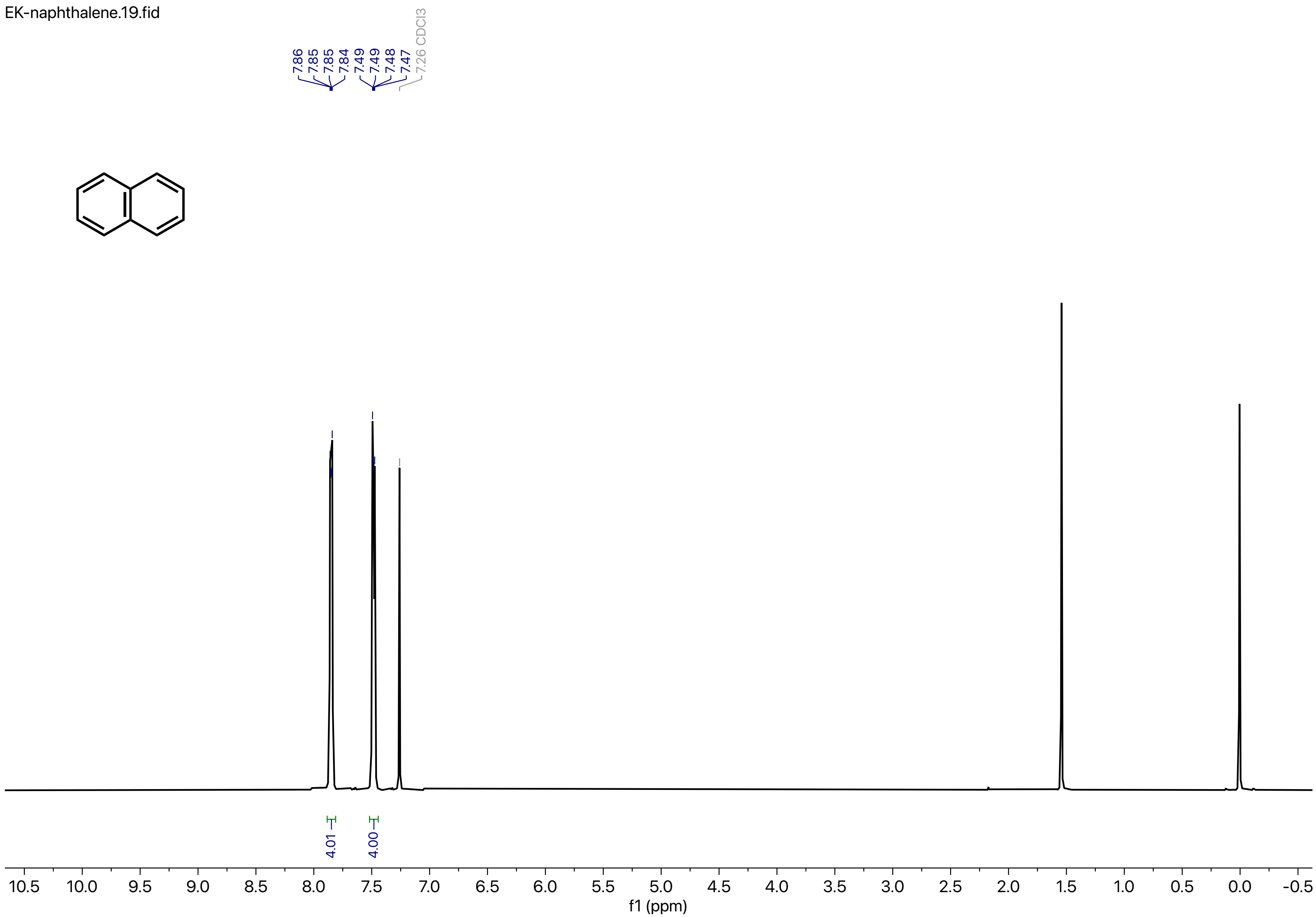

Figure S30. **^{1}H NMR** spectrum (500 MHz, $CDCl_3$, 298 K) of Naphthalene.

10.0 Single Crystal X-ray Data (3e-3h and 5)

10.1 X-ray crystallography, complexes (3e-3h)

Single crystal X-ray diffraction data were collected on well-formed crystals of complexes **3e-3h** using microfocus X-ray sources (Cu-Kα for **3f** and **3g**; Mo-Kα for **3e** and **3h**). Bruker D8 Venture (Photon 2 detector) and Bruker D8 Quest (Photon 3 detector) diffractometers were used to measure the data using phi and omega scans of 0.5° frame width. Data were collected, integrated (SAINT), and corrected for absorption (SADABS) within the Apex 3 software suite.[9] Structure solution by intrinsic phasing (SHELXT) and refinement on F^2 using full matrix least squares techniques (SHELXL) were accomplished using the SHELXTL software package.[10,11] Reflections obscured by the beamstop were omitted from the refinements. All non-hydrogen atoms were refined anisotropically. Hydrogen atoms were placed in calculated positions and refined isotropically using appropriate riding models. The crystals of **3e** and **3f** were found to be acetonitrile solvates, each with one acetonitrile per formula unit, and those solvent molecules were refined routinely in full occupancy. The crystal of **3h** was found to be the chloroform solvate, also with one chloroform molecule per formula unit routinely refined in full occupancy. Also in **3h**, one of the 2-fluorobenzyl ligands exhibited disorder of its fluorine atom. This fluorine/hydrogen disorder was refined in separate parts and the site occupancy of the major:minor disorder contribution was refined as a free variable in a ratio of 0.701(6):0.299(6). Similarity restraints were used to maintain chemically reasonable relationships between the anisotropic displacement parameters of the fluorine atoms in the disordered parts. Crystallographic data are available from the Cambridge Crystallographic Data Centre, deposition numbers 2475783-2475786.

Table S6. Crystallographic data for complexes **3e-3h**.

	**3e · MeCN**	**3f · MeCN**	**3g**	**3h · CHCl₃**
Empirical formula	$C_{32}H_{29}Br_2N_5Ni$	$C_{32}H_{41}Br_2N_5Ni$	$C_{38}H_{30}Br_2N_4Ni$	$C_{31}H_{25}Br_2Cl_3F_2N_4Ni$
Formula weight	702.13	714.23	761.19	816.43
Temperature (K)	100(2)	100(2)	100(2)	100(2)
Crystal system	monoclinic	triclinic	monoclinic	monoclinic
Space group	$P2_1/c$	P-1	$P2_1/c$	$P2_1/c$
a (Å)	19.1202(12)	10.5629(9)	11.8450(4)	17.7030(5)
b (Å)	8.4510(6)	12.5475(11)	17.6450(6)	13.6694(4)
c (Å)	18.4658(11)	13.1230(12)	15.0245(5)	13.1493(4)
α (°)	90	88.223(4)	90	90
β (°)	106.000(2)	73.887(3)	90.776(2)	101.7813(9)
γ (°)	90	67.181(3)	90	90
Volume ($Å^3$)	2868.2(3)	1534.4(2)	3139.91(18)	3114.96(16)
Z	4	2	4	4
ρ_{calc} (g/cm^3)	1.626	1.546	1.610	1.741
μ (mm^{-1})	3.494	4.188	4.139	3.487
F(000)	1416	732	1536	1624
Crystal size (mm)	0.11 × 0.23 × 0.29	0.07 × 0.17 × 0.19	0.11 × 0.16 × 0.19	0.04 × 0.05 × 0.24
Radiation	Mo Kα (λ = 0.71073 Å)	Cu Kα (λ = 1.54178 Å)	Cu Kα (λ = 1.54178 Å)	Mo Kα (λ = 0.71073 Å)
2Θ range for data collection (°)	5.306 to 56.710	9.486 to 141.086	8.992 to 140.334	5.910 to 54.224
Index ranges	-25 ≤ h ≤ 25, -11 ≤ k ≤ 11, -24 ≤ l ≤ 24	-12 ≤ h ≤ 12, -15 ≤ k ≤ 15, -15 ≤ l ≤ 15	-14 ≤ h ≤ 14, -21 ≤ k ≤ 21, -18 ≤ l ≤ 18	-22 ≤ h ≤ 22, -17 ≤ k ≤ 17, -16 ≤ l ≤ 16
Reflections collected	68305	33075	52622	81924
Independent reflns.	7150 [R_{int} = 0.0597, R_{sigma} = 0.0292]	5813 [R_{int} = 0.0527, R_{sigma} = 0.0335]	5957 [R_{int} = 0.0555, R_{sigma} = 0.0282]	6861 [R_{int} = 0.0906, R_{sigma} = 0.0493]
Data/restr./param.	7150 / 0 / 362	5813 / 0 / 362	5957 / 0 / 406	6861 / 12 / 398
Goodness-of-fit on F^2	1.031	1.033	1.051	1.027
R1, wR2 [I>=2σ (I)]	0.0296, 0.0767	0.0272, 0.0691	0.0343, 0.0927	0.0386, 0.0808
R1, wR2 [all data]	0.0360, 0.0802	0.0290, 0.0702	0.0395, 0.0966	0.0561, 0.0916
Largest diff. peak, hole ($eÅ^{-3}$)	0.947, -0.670	0.433, -0.778	0.870, -0.817	1.376, -0.967
CCDC Deposition No.	2475783	2475784	2475785	2475786

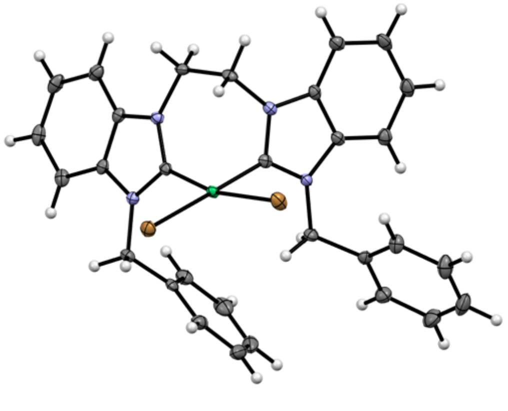

3e

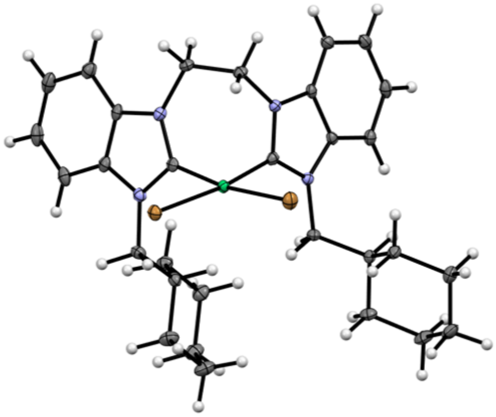

3f

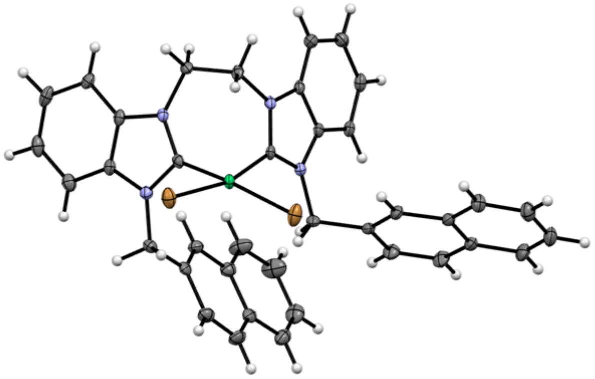

3g

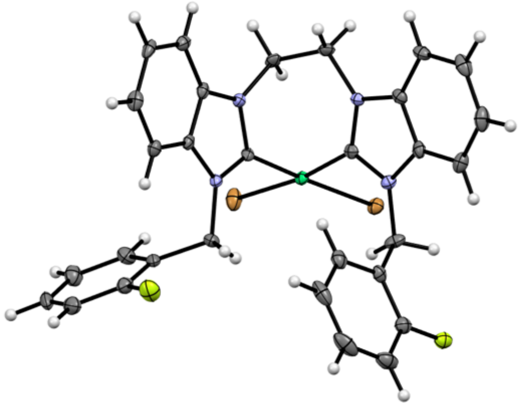

3h

Figure S31. Solid-state structures for **3e**-**3h**, shown as 50% probability ellipsoids (solvent molecules omitted for clarity). Ni atoms are green, Br atoms are orange, N atoms are purple, C atoms are gray, F atoms are lime green, and H atoms are white.

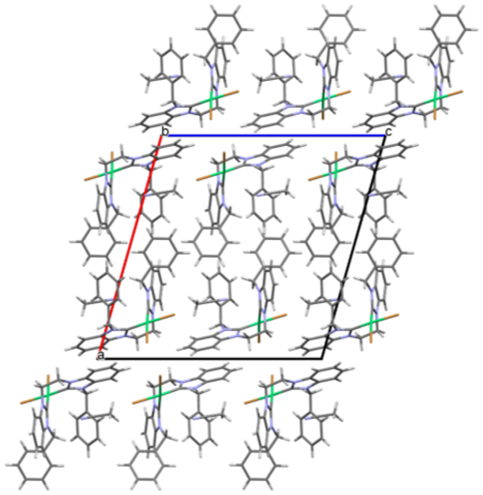

3e

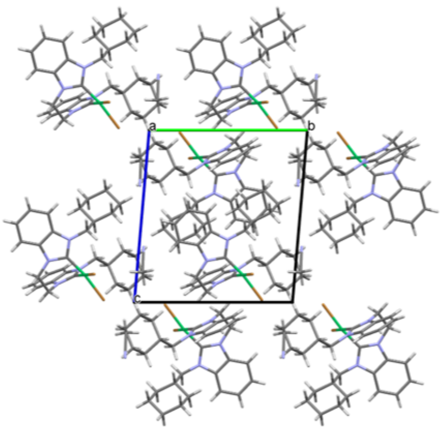

3f

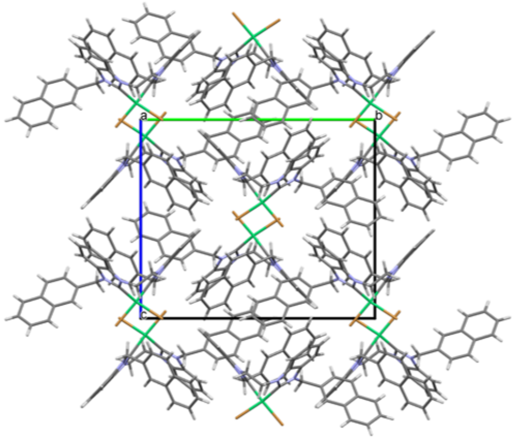

3g

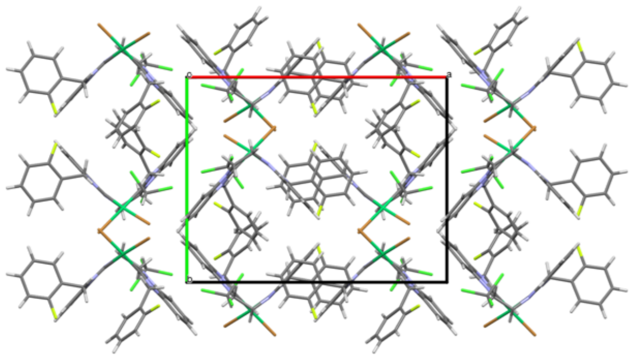

3h

Figure S32. Solid-state packing diagrams for **3e**-**3h**, viewed along the *b*-axis for **3e**, *a*-axis for **3f** and **3g**, and *c*-axis for **3h**. Ni atoms are green, Br atoms are orange, N atoms are purple, C atoms are gray, F atoms are lime green, and H atoms are white. Solvent molecules are included in the packing diagrams, with C atoms shown in gray, N atoms in purple, Cl atoms in light green, and H atoms in white.

Table S7. Selected interatomic distances (Å) and angles (°) in **3e-3h**.

	**3e**	**3f**	**3g**	**3h**
Ni1−Br1	2.3533(4)	2.3663(4)	2.3594(5)	2.3415(5)
Ni1−Br2	2.3673(3)	2.3431(4)	2.3374(5)	2.3692(5)
Ni1−C1	1.851(2)	1.873(2)	1.861(3)	1.856(3)
Ni1−C2	1.878(2)	1.856(2)	1.859(3)	1.880(3)
Br1−Ni1−Br2	92.71(1)	94.24(1)	95.80(2)	94.44(2)
Br1−Ni1−C1	84.49(6)	89.18(6)	165.36(8)	89.69(10)
Br1−Ni1−C2	167.22(6)	173.18(6)	86.92(8)	171.86(10)
Br2−Ni1−C1	176.54(6)	171.57(7)	93.07(8)	175.87(10)
Br2−Ni1−C2	95.34(6)	89.81(6)	173.09(8)	90.95(10)
C1−Ni1−C2	87.07(9)	86.08(9)	85.61(11)	84.92(14)

10.2 X-ray crystallography, bis(NHC)Ni^{I}Br (5)

Orange, X-ray quality crystals of complex **5** were obtained by layering diethyl ether over a solution in tetrahydrofuran. The structure determination was performed on an Oxford Diffraction Gemini-R diffractometer, using Cu-Kα radiation at 110 K. A crystal was mounted on a Hampton Research Cryoloop using Paratone-N oil. Unit cell determination, data collection and reduction, and analytical absorption correction was performed using the CrysAlisPro software package.[30] Structure solution (SHELXT)[10] and refinement (SHELXL)[11] was conducted using Olex2 1.5.[31] All non-hydrogen atoms were refined anisotropically. Hydrogen atoms were placed in calculated positions and refined using a riding model. Because the crystal was very small, an extended (four-day) data collection resulted in a signal-to-noise ratio of only 8.5. Crystallographic data may be obtained in CIF form from the Cambridge Crystallographic Data Centre, deposition number CCDC 2472042.

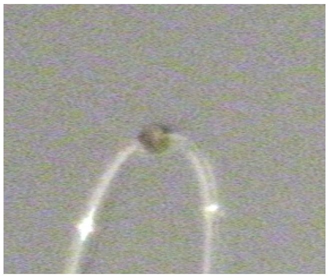

Table S8. Crystal data and structure refinement for complex **5**.

	**Complex 5**
Empirical formula	$C_{31}H_{40}BrN_4Ni$
Formula weight	607.29
Temperature (K)	120(16)
Crystal system	triclinic
Space group	*P-1*
a (Å)	10.8347(6)
b (Å)	13.2080(8)
c (Å)	20.0101(8)
α (°)	94.703(4)
β (°)	93.700(4)
γ (°)	96.485(5)
Volume (Å^3)	2827.8(3)
Z	4
ρ_{calc} (g/cm^3)	1.426
μ (mm^{-1})	2.838
F(000)	1268.0
Crystal size (mm^3)	0.053 × 0.023 × 0.02
Radiation	Cu Kα (λ = 1.54184 Å)
2Θ range for data collection (°)	7.752 to 135.432
Index ranges	-12 ≤ h ≤ 12, -15 ≤ k ≤ 15, -23 ≤ l ≤ 20
Reflections collected	50178
Independent reflections	10155 [R_{int} = 0.1735, R_{sigma} = 0.1178]
Data/restraints/parameters	10155/0/667
Goodness-of-fit on F^2	1.018
R1, wR2 [I>=2σ (I)]	0.0611, 0.1309
R1, wR2 [all data]	0.1247, 0.1716
Largest diff. peak, hole (eÅ^{-3})	2.00, -1.13
CCDC Deposition No.	2472042

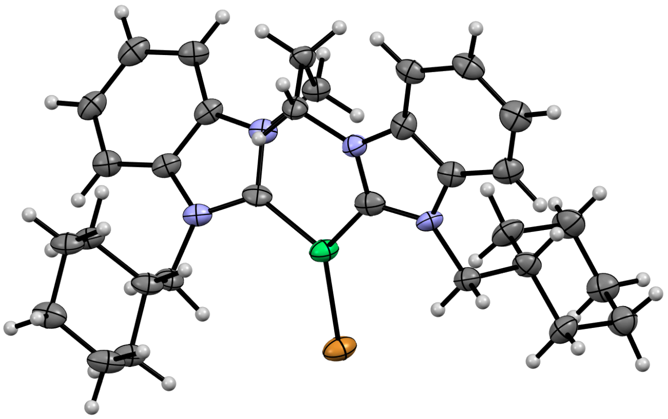

Figure S33. Solid-state structure for complex **5** shown as 50% probability ellipsoids. The Ni atom is green, Br atom is orange, N atoms are purple, C atoms are gray and H atoms are white. Selected bond lengths (Å): C-Ni = 1.924(7) and Ni-Br = 2.3600(12). Selected bond angles (°): C-Ni-C = 109.8(3) and C-Ni-Br = 124.7(2). Averaged values of the two molecules in the asymmetric unit are reported.

11.0 DFT Calculations

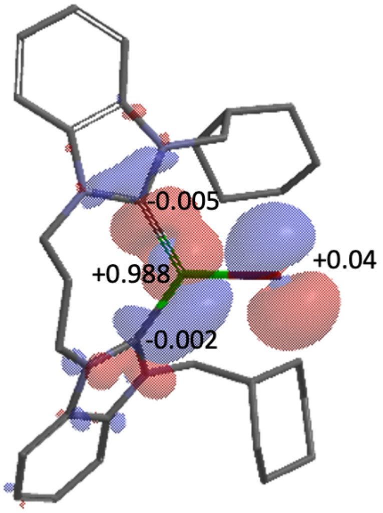

Figure S34. Spin density plot of **5**. Mulliken spin density population indicated for selected atoms. Geometry optimizations and single-point energy calculations were carried out at a fixed geometry of **5** given by the crystallographic coordinates at the B3LYP level of theory with the basis set 6-31G* using the Spartan 24 program.

12.0 References

(1) Bera, S.; Bera, A.; Banerjee, D. Nickel-Catalyzed Dehydrogenation of N-Heterocycles Using Molecular Oxygen. *Org. Lett.* **2020**, *22* (16), 6458-6463.
(2) Raja, D.; Philips, A.; Palani, P.; Lin, W.-Y.; Devikala, S.; Senadi, G. C. Metal-Free Synthesis of Benzimidazoles via Oxidative Cyclization of d-Glucose with o-Phenylenediamines in Water. *J. Org. Chem* **2020**, *85* (17), 11531-11540.
(3) Muñoz, S. B.; Foster, W. K.; Lin, H.-J.; Margarit, C. G.; Dickie, D. A.; Smith, J. M. Tris(carbene)borate Ligands Featuring Imidazole-2-ylidene, Benzimidazol-2-ylidene, and 1,3,4-Triazol-2-ylidene Donors. Evaluation of Donor Properties in Four-Coordinate {NiNO}10 Complexes. *Inorg. Chem.* **2012**, *51* (23), 12660-12668.
(4) Zhang, C. S.; Rajesh, S.; Moffett, A. L.; Chen, T. H.; McMillen, C. D.; Green, K.-A. Bis(benzimidazolin-2-ylidenes): Exploring the Impact of Ligand Properties in the Nickel-Catalyzed Suzuki–Miyaura Coupling. *Organometallics* **2024**, *43* (18), 1988-2001.
(5) Quasdorf, K. W.; Riener, M.; Petrova, K. V.; Garg, N. K. Suzuki−Miyaura Coupling of Aryl Carbamates, Carbonates, and Sulfamates. *J. Am. Chem. Soc.* **2009**, *131* (49), 17748-17749.
(6) Leowanawat, P.; Zhang, N.; Resmerita, A.-M.; Rosen, B. M.; Percec, V. $Ni(COD)_2/PCy_3$ Catalyzed Cross-Coupling of Aryl and Heteroaryl Neopentylglycolboronates with Aryl and Heteroaryl Mesylates and Sulfamates in THF at Room Temperature. *J. Org. Chem* **2011**, *76* (24), 9946-9955.
(7) Fulmer, G. R.; Miller, A. J. M.; Sherden, N. H.; Gottlieb, H. E.; Nudelman, A.; Stoltz, B. M.; Bercaw, J. E.; Goldberg, K. I. NMR Chemical Shifts of Trace Impurities: Common Laboratory Solvents, Organics, and Gases in Deuterated Solvents Relevant to the Organometallic Chemist. *Organometallics* **2010**, *29* (9), 2176-2179.
(8) Babij, N. R.; McCusker, E. O.; Whiteker, G. T.; Canturk, B.; Choy, N.; Creemer, L. C.; Amicis, C. V. D.; Hewlett, N. M.; Johnson, P. L.; Knobelsdorf, J. A.; et al. NMR Chemical Shifts of Trace Impurities: Industrially Preferred Solvents Used in Process and Green Chemistry. *Org. Process Res. Dev.* **2016**, *20* (3), 661-667.
(9) Apex3; Bruker AXS Inc. Madison, WI, USA, 2015.
(10) Sheldrick, G. SHELXT - Integrated space-group and crystal-structure determination. *Acta Crystallogr A* **2015**, *71* (1), 3-8.
(11) Sheldrick, G. Crystal structure refinement with SHELXL. *Acta Crystallographica Section C* **2015**, *71* (1), 3-8.
(12) Berding, J.; Lutz, M.; Spek, A. L.; Bouwman, E. Synthesis of Novel Chelating Benzimidazole-Based Carbenes and Their Nickel(II) Complexes: Activity in the Kumada Coupling Reaction. *Organometallics* **2009**, *28* (6), 1845-1854.
(13) Penney, A. A.; Sizov, V. V.; Grachova, E. V.; Krupenya, D. V.; Gurzhiy, V. V.; Starova, G. L.; Tunik, S. P. Aurophilicity in Action: Fine-Tuning the Gold(I)–Gold(I) Distance in the Excited State To Modulate the Emission in a Series of Dinuclear Homoleptic Gold(I)–NHC Complexes. *Inorg. Chem.* **2016**, *55* (10), 4720-4732.
(14) Tobisu, M.; Yamakawa, K.; Shimasaki, T.; Chatani, N. Nickel-catalyzed reductive cleavage of aryl–oxygen bonds in alkoxy- and pivaloxyarenes using hydrosilanes as a mild reducing agent. *Chem. Commun.* **2011**, *47* (10), 2946-2948.
(15) Tobisu, M.; Morioka, T.; Ohtsuki, A.; Chatani, N. Nickel-catalyzed reductive cleavage of aryl alkyl ethers to arenes in absence of external reductant. *Chem. Sci.* **2015**, *6* (6), 3410-3414.

(16) Ackermann, L.; Kapdi, A. R.; Fenner, S.; Kornhaaß, C.; Schulzke, C. Well-Defined Air-Stable Palladium HASPO Complexes for Efficient Kumada–Corriu Cross-Couplings of (Hetero)Aryl or Alkenyl Tosylates. *Chem. Eur. J.* **2011**, *17* (10), 2965-2971.
(17) Beromi, M. M.; Nova, A.; Balcells, D.; Brasacchio, A. M.; Brudvig, G. W.; Guard, L. M.; Hazari, N.; Vinyard, D. J. Mechanistic Study of an Improved Ni Precatalyst for Suzuki–Miyaura Reactions of Aryl Sulfamates: Understanding the Role of Ni(I) Species. *J. Am. Chem. Soc.* **2017**, *139* (2), 922-936.
(18) Shi, S.; Meng, G.; Szostak, M. Synthesis of Biaryls through Nickel-Catalyzed Suzuki–Miyaura Coupling of Amides by Carbon–Nitrogen Bond Cleavage. *Angew. Chem. Int. Ed.* **2016**, *55* (24), 6959-6963.
(19) Gurung, S. K.; Thapa, S.; Kafle, A.; Dickie, D. A.; Giri, R. Copper-Catalyzed Suzuki–Miyaura Coupling of Arylboronate Esters: Transmetalation with (PN)CuF and Identification of Intermediates. *Org. Lett.* **2014**, *16* (4), 1264-1267.
(20) Delaney, C. P.; Kassel, V. M.; Denmark, S. E. Potassium Trimethylsilanolate Enables Rapid, Homogeneous Suzuki−Miyaura Cross-Coupling of Boronic Esters. *ACS Catal.* **2020**, *10* (1), 73-80.
(21) Kuriyama, M.; Kujirada, S.; Tsukuda, K.; Onomura, O. Nickel-Catalyzed Deoxygenative Deuteration of Aryl Sulfamates. *Adv. Synth. Catal.* **2017**, *359* (6), 1043-1048.
(22) Sacco, A.; Mastrorilli, P. Reactivity of nickel (II) diphosphine complexes towards alkoxides: a new route to the synthesis of nickel(0) compounds through nickel(II) alkoxides. *J. Chem. Soc., Dalton Trans.* **1994**, (19), 2761-2764.
(23) Fantasia, S.; Nolan, S. P. A General Synthetic Route to Mixed NHC–Phosphane Palladium(0) Complexes (NHC=N-Heterocyclic Carbene). *Chem. Eur. J.* **2008**, *14* (23), 6987-6993.
(24) Bent, H. A. An Appraisal of Valence-bond Structures and Hybridization in Compounds of the First-row elements. *Chem. Rev.* **1961**, *61* (3), 275-311.
(25) Lim, M. Q.; Brick, K. J.; LeBlanc, J.; Garrard, C.; Keske, E. C. Preparation and Metalation of N-Heterocyclic Carbene Ligands with Sterically Tunable 2,6-dialkoxyphenyl Wingtip Groups. *Organometallics* **2025**, *44* (1), 82-93.
(26) Meng, G.; Kakalis, L.; Nolan, S. P.; Szostak, M. A simple 1H NMR method for determining the σ-donor properties of N-heterocyclic carbenes. *Tetrahedron Lett.* **2019**, *60* (4), 378-381.
(27) Poater, A.; Ragone, F.; Giudice, S.; Costabile, C.; Dorta, R.; Nolan, S. P.; Cavallo, L. Thermodynamics of N-Heterocyclic Carbene Dimerization: The Balance of Sterics and Electronics. *Organometallics* **2008**, *27* (12), 2679-2681.
(28) Falivene, L.; Cao, Z.; Petta, A.; Serra, L.; Poater, A.; Oliva, R.; Scarano, V.; Cavallo, L. Towards the online computer-aided design of catalytic pockets. *Nat. Chem.* **2019**, *11* (10), 872-879.
(29) Poater, A.; Ragone, F.; Mariz, R.; Dorta, R.; Cavallo, L. Comparing the Enantioselective Power of Steric and Electrostatic Effects in Transition-Metal-Catalyzed Asymmetric Synthesis. *Chem. Eur. J.* **2010**, *16* (48), 14348-14353.
(30) *Oxford Diffraction (2007). Oxford Diffraction Ltd., Xcalibur CCD system, CrysAlisPro Software system, Version 1.171.32.*
(31) Dolomanov, O. V.; Bourhis, L. J.; Gildea, R. J.; Howard, J. A. K.; Puschmann, H. OLEX2: a complete structure solution, refinement and analysis program. *J. Appl. Cryst.* **2009**, *42* (2), 339-341.
